# Absence of DEATH kinesin is fatal for *Leishmania mexicana* amastigotes

**DOI:** 10.1038/s41598-022-07412-z

**Published:** 2022-02-28

**Authors:** Suad Gazi Jaafer Husaine Al Kufi, Josiah Emmerson, Heidi Rosenqvist, Catarina Mateus Moreira Garcia, Diana Onodelia Rios-Szwed, Martin Wiese

**Affiliations:** 1 Strathclyde Institute of Pharmacy and Biomedical Sciences, University of Strathclyde, Glasgow, UK; 2 Present Address: Department of Laboratory Investigations, Faculty of Science, University of Kufa, Kufa, Iraq; 3 Present Address: MRC Human Genetics Unit, Institute of Genetics and Cancer, University of Edinburgh, Edinburgh, UK

**Keywords:** Proteins, Phosphorylation

## Abstract

Kinesins are motor proteins present in organisms from protists to mammals playing important roles in cell division, intracellular organisation and flagellum formation and maintenance. *Leishmania mexicana* is a protozoan parasite of the order Kinetoplastida causing human cutaneous leishmaniasis. Kinetoplastida genome sequence analyses revealed a large number of kinesins showing sequence and structure homology to eukaryotic kinesins. Here, we investigate the *L. mexicana* kinesin LmxKIN29 (LmxM.29.0350), also called DEATH kinesin. The activated MAP kinase LmxMPK3, a kinase affecting flagellum length in *Leishmania*, is able to phosphorylate recombinant full length LmxKIN29 at serine 554. Insect promastigote LmxKIN29 *Leishmania* null mutants showed no obvious phenotype. However, in mouse infection experiments, the null mutants were unable to cause the disease, whereas LmxKIN29 add-backs and single allele knockouts caused footpad lesions. Localisation using promastigotes expressing GFP-tagged LmxKIN29 revealed that the kinesin is predominantly found in between the nucleus and the flagellar pocket, while in dividing cells the GFP-fusion protein was found at the anterior and posterior ends of the cells indicating a role in cytokinesis. The inability to cause lesions in infected animals and the amino acid sequence divergence from mammalian kinesins suggests that LmxKIN29 is a potential drug target against leishmaniasis.

## Introduction

Kinesins constitute a superfamily of molecular motor proteins that convert the energy from ATP hydrolysis into mechanical work to transport a variety of cargo along microtubules, e.g. transport vesicles, organelles, and chromosomes^1^. Kinesins also play an important role in cell division by regulating the mitotic spindle formation, polymerising and depolymerising spindle microtubules, movement of chromosomes, and antiparallel spindle movements during mitosis and meiosis^2^. Defects in kinesin function can lead to neurodegeneration, cancer, developmental defects and ciliopathies^3^. Uncontrolled cell division is a hallmark of cancer cells and kinesins along with microtubules are at the heart of this process. Hence, kinesins are potential therapeutic targets and a lot of effort has gone into the identification of specific small molecule inhibitors for mitotic kinesins^4^. Kinesins possess a highly conserved globular motor domain of ~ 340 amino acids, which contains an ATP-binding and a microtubule-binding domain^1^. Kinesins are classified based on the position of the motor domain within the molecule. N-kinesins have a motor domain in the NH_2_-terminal region, M-kinesins have the motor in the middle region, and C-kinesins have a COOH-terminal motor domain^2^. Unlike the motor domain, the tail domains of most kinesins are highly divergent^3^. A standardised kinesin nomenclature originally defined 14 families (kinesin-1–14), as well as some ‘orphan’ kinesins but was later extended to 17 families^5,6^. Kinetoplastida genome sequences have shown the presence of a large number of kinesin motor proteins, with for instance 41 kinesin proteins in *Trypanosoma brucei*^7^. Kinesins have been shown to undergo phosphorylation by a variety of protein kinases, like mitogen-activated protein kinases, Ca^2+^/calmodulin-dependent protein kinase II (CAMK-II), cyclin-dependent protein kinase 1 (CDK1)—cyclin B, glycogen synthase kinase β, Aurora kinases, NIMA kinase NEK6, and Polo-like kinase (PLK), which can be involved in relief of autoinhibition, loading and unloading of cargo, and regulation of kinesin activity^3,8,9^. Very little is known about kinesins of the protozoan parasite *Leishmania mexicana* the causative agent of cutaneous leishmaniasis. An estimated 700,000 to 1 million new cases of leishmaniasis occur annually^10^. There is currently no vaccine and successful treatment requires an immunocompetent individual to control persisting parasites. Hence, new drug targets and more efficient drugs are required to eliminate the disease. Here, we aimed to functionally characterise the *L. mexicana* orphan kinesin LmxKIN29, which is encoded on chromosome 29 (LmxM.29.0350). We predictively called it DEATH kinesin, because of the presence of the amino acids D, E, A, T, H in this order in its sequence. We could show by deletion analysis that it is essential for survival of the parasite in the infected host.

## Results and discussion

### Characterisation of the kinesin LmxKIN29 (LmxM.29.0350)

Very little was known about the putative kinesin of *L. mexicana* with the accession number LmxM.29.0350, which is encoded on chromosome 29 (TriTrypDB). mRNA has been found in all *L. mexicana* life stages, with amastigotes in in vitro infected macrophages showing the highest level compared to promastigotes and axenic amastigotes^11^. Phosphoproteomics of promastigotes and amastigotes of *L. mexicana* were carried out as described before^12^ and showed that the kinesin is phosphorylated on serine 548, serine 551 and serine 554 (Supplementary File 1). In promastigotes only peptides revealing a single phosphorylation on serine 551 or serine 554 could be detected. By contrast, in in vitro differentiated axenic amastigotes 72 h post differentiation initiation by temperature and pH shift single phosphorylation was detected on all three serine residues and dual phosphorylation was found for serine 548 and 551, and serine 551 and 554, but not serine 548 and 554. The presence of LmxKIN29 in amastigotes isolated from pooled lesion material of infected Balb/c mice could also be shown by identification of five tryptic peptides, but no phosphorylated peptides were detected in these samples (Supplementary File 1). Phosphorylation of LmxKIN29 might occur in a specific stage during the cell cycle, which might not be as abundant in lesion-derived amastigotes as it is in axenic amastigotes and promastigotes. Deuterium labelling of deoxyribose and incorporation into DNA showed that *L. mexicana* amastigotes in mouse lesions have a slow doubling time of 12 days, compared to 4.2 days in axenic amastigotes measured at 96 h post induction of differentiation, and 9 h in logarithmically growing promastigotes^13^. Slowing down proliferation in a lesion is likely to have an influence on the abundance of certain proteins and their phosphorylation state. Phosphorylation at various and multiple residues might affect the function, interaction with binding partners/cargo and/or localisation of LmxKIN29. Each phosphorylation site might be phosphorylated by a specific kinase leading to a possible integration of different signals. With a proline following each of the three serine residues, all identified phosphorylation sites resemble a phosphorylation site used by mitogen-activated protein (MAP) kinases. Therefore, the recombinantly expressed MAP kinase, LmxMPK3, activated by co-expression with LmxMKK^14^ was tested in a radiometric kinase assay on glutathione-S-transferase fused to a peptide carrying the LmxKIN29 phosphorylation sites, resulting in a strong phosphorylation signal of the GST-peptide fusion (Fig. S1). Given that loss of LmxMPK3 MAP kinase has been previously associated with shorter promastigote flagella^14^, it was speculated that LmxKIN29 might also be involved in flagellum length regulation. mRNA of LmxMPK3 was absent in lesion-derived amastigotes^15^ and no protein was detectable by immunoblot analysis^14^. Moreover, protein levels of LmxMPK3 fell below the level of detection by immunoblot analysis 48 h post induction of differentiation to axenic amastigotes^14^. These observations agree with the absence of phosphorylation in lesion-derived amastigotes. Whether the LmxKIN29 phosphorylations detected in axenic amastigotes are caused by different kinases or have been caused by LmxMPK3 before the kinase was removed during differentiation is unclear and further investigations will have to look into LmxKIN29 turnover and its phosphorylations.

**Figure 1:**
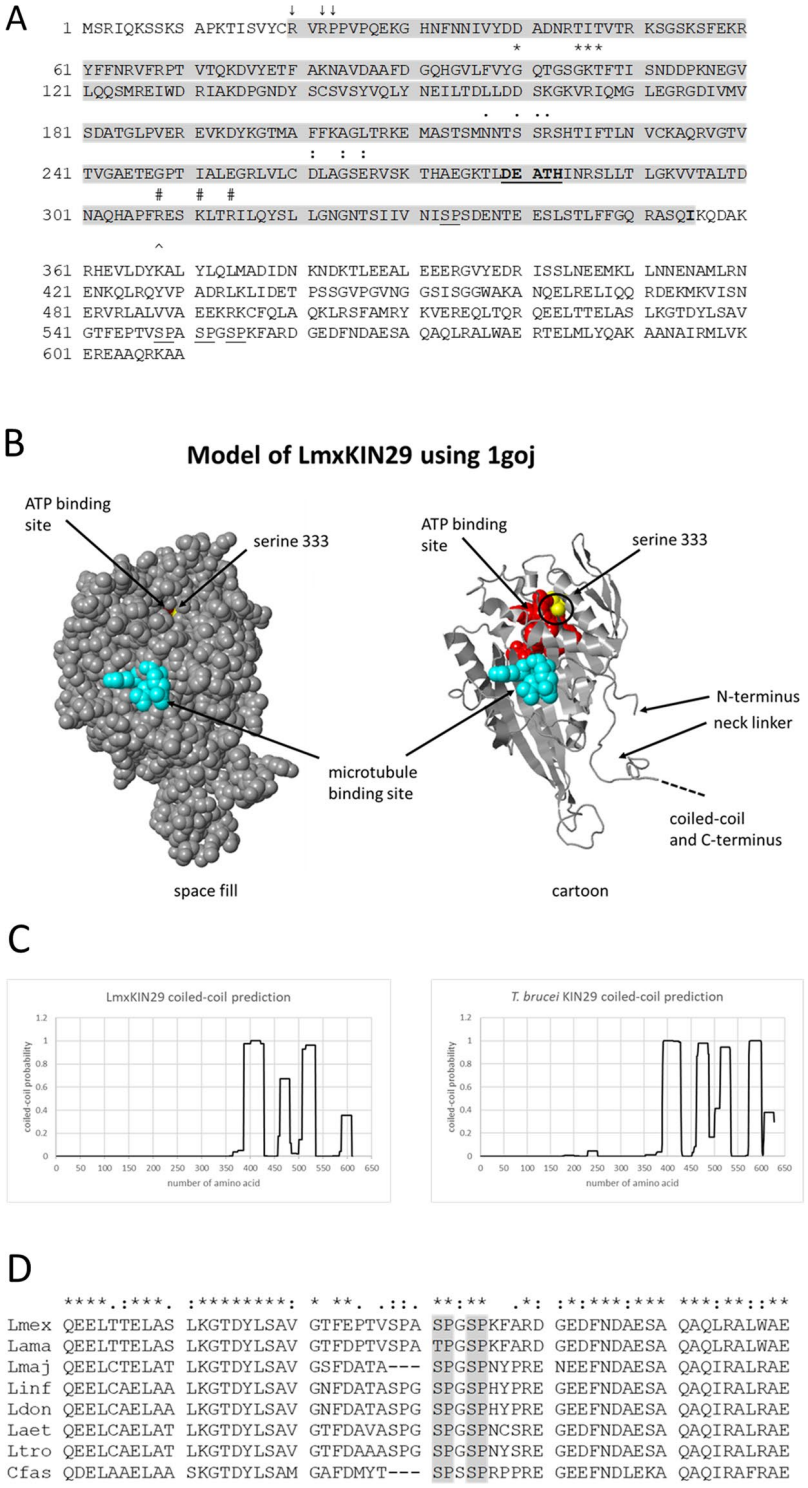
Features of LmxKIN29. (**A**) amino acid sequence of LmxKIN29 with putative MAP kinase phosphorylation sites underlined (serine 333, threonine 440, serine 548, serine 551, and serine 554), the motor domain (R20-I355) is shown in grey (pfam00225), the DEATH motif (D279–H283) is bold and underlined. R20, R22, P23 (↓), G100-F108 (*, Walker A motif GxxxxGKT/S), the switch-1 motif (NxxSSR)(.) and D261 of the switch-2 motif (DxxGxE)(:) are involved in ATP-binding; R308, K311, and R314 are residues of the microtubule-binding site (#); I355 in bold is involved in neck linker binding; K368 marks the start of the coiled-coil dimerisation domain (^). (**B**) 3D model of LmxKIN29 using 1goj in SWISS-MODEL and displayed by Jmol FirstGlance 3.0 (http://firstglance.jmol.org) showing the localisation of the neck linker, of the ATP-binding and microtubule-binding sites, and of serine 333. (**C**) coiled-coil prediction for LmxKIN29 (left) and its *T. brucei* orthologue (right) using COILS version 2.2 and a window of 21 amino acids. (**D**) partial amino acid sequence alignment of LmxKIN29 from *L. mexicana* with the amino acid sequences of various kinetoplastid kinesins. Lmex, LmxM.29.0350 *Leishmania mexicana* MHOM/GT/2001/U1103; Lama, LAMA_000589400 *Leishmania amazonensis* MHOM/BR/71973/M2269; Lmaj, LmjF.30.0350 *Leishmania major* strain Friedlin; Linf, LINF_300008500-T1 *Leishmania infantum* JPCM5; Ldon, LdBPK_300350.1.1 *Leishmania donovani* BPK282A1; Laet, LAEL147_000550000 *Leishmania aethiopica* L147; Ltro, LTRL590_300009000 *Leishmania tropica* L590; Cfas, CFAC1_260021700 *Crithidia fasciculata* strain Cf-Cl. The asterisk (*) indicates conserved amino acid residues in all orthologues (alignment by Clustal Omega modified).

The open reading frame (ORF) of LmxKIN29 comprises 1,833 bp encoding a protein of 610 amino acids (Fig. 1A) with a calculated molecular mass of 68.3 kDa and an isoelectric point of 8.17^16^. The primary structure contains typical kinesin domains; an N-terminal motor domain, a coiled-coil sequence with a neck linker and a C-terminal domain presumably involved in cargo binding (Fig. 1A). The eponymous DEATH motif is from aspartate 279 to histidine 283 and is conserved in *Leishmania* and *Trypanosoma* species. Whether it is involved in the function of this kinesin has not yet been investigated. Conserved residues involved in ATP-binding are R20, R22, P23, the residues forming the Walker A motif (GxxxxGKT/S) G100, G105, K106 and T107, and D261 of the switch-2 motif (DxxGxE). G264 and E266 complete the switch-2 motif in LmxKIN29. Moreover, the switch-1 motif (NxxSSR) is also present with N217, S220, S221, R222. The conformation of the switch-1 and switch-2 motifs is dependent on the presence of the ATP γ-phosphate^2^. The ATP β-phosphate is bound by the phosphate loop (P-loop) residues of the Walker A motif. I355 at the end of the kinesin motor domain likely corresponds to I327 in rat conventional kinesin, which binds into a pocket in the motor core to induce zippering the neck linker onto the motor core^17^. In addition to the three serine residues described above (S548, S551, S554) the primary sequence revealed another serine and one threonine residue each followed by a proline constituting potential MAP kinase phosphorylation sites (Fig. 1A) with serine 333 located within the motor domain. Modelling the structure of the LmxKIN29 motor domain using SWISS-MODEL and the best matching structure of the *Neurospora crassa* kinesin (NcKin) (PDB 1goj) revealed that this serine is unlikely to be accessible for phosphorylation by a protein kinase and hence was excluded from mutational analysis^18,19^ (Fig. 1B; Fig. S2 showing different orientations). The structure of the model also fits very well with the structure of rat conventional kinesin showing corresponding secondary structure elements, with only the regions in the periphery around loops L2 and L8 showing some differences^20^ (Fig. S3, alignment of LmxKIN29 with rat and *Neurospora* kinesins with secondary structure elements as in model). The model shows the position of the ATP-binding site accessible from the opposite side of the molecule than the microtubule-binding site. The region containing the neck linker is also shown. COILS version 2.2 was used to predict coiled-coil structures in LmxKIN29 and its *T. brucei* orthologue TbKIN29 (Tb927.6.1770) (Fig. 1C)^21^. Threonine 440 is localised in a gap between coiled-coil elements and might therefore be accessible for phosphorylation (Fig. 1C). Serines 548, 551, and 554 are localised after the main coiled-coil structure of LmxKIN29 starting with glutamine 357 and ending with aspartate 535 (Fig. 1A) suggesting that they are accessible for phosphorylation by a protein kinase. Serine 553 and serine 556 of TbKIN29 were shown to be phosphorylated in bloodstream form trypanosomes^22^ and are also localised after the main coiled-coil region (Fig. 1C). A sequence alignment of the region containing serines 548, 551 and 554 of LmxKIN29 homologues from different Kinetoplastida species shows that serine 551 and serine 554 are highly conserved (Fig. 1D) and their phosphorylation most likely constitutes a mechanism to regulate this kinesin’s function.

LmxKIN29, like its orthologue in *T. brucei* Tb927.6.1770 and *L. major* LmjF.30.0350, is an orphan kinesin^6,23^. Overall, LmxKIN29 shows high levels of amino acid identity with LmxKIN29 orthologues in other *Leishmania* species such as, 99% with *L. amazonensis*, 87% with *L. donovani* and *L. infantum*, 86% with *L. aethiopica* and *L. tropica*, 84% with *L. major,* 78% with *L. tarentolae* and 75% with *L. braziliensis* (Supplementary File 2). The closest human kinesins showed low similarities of their motor domains to LmxKIN29, e.g. KIF11 showed 36% and KIF3A 38% amino acid identity. The low percentage of identical amino acids between the motor domain of LmxKIN29 and the motor domains of kinesins in higher eukaryotes suggests that this protein could be used as a target for *Leishmania* kinesin-specific inhibitors, which can be developed into new drugs against the pathogen, provided that LmxKIN29 is essential in the mammalian amastigote stage. Closer analysis revealed that the ATP-binding pocket residues and the residues involved in microtubule binding are highly conserved and hence inhibitors would be best targeted at sites preventing dimerisation, phosphorylation or cargo binding. LmxKIN29 did not show any significant sequence identity with the two known types of flagellar kinesins in higher eukaryotes, the flagellar heterotrimeric Kinesin-II and the homodimeric kinesin OSM3. However, whether LmxKIN29 is a kinesin specific for flagellar function in *L. mexicana* still needed testing.

### Purification of recombinant LmxKIN29

**Figure 2:**
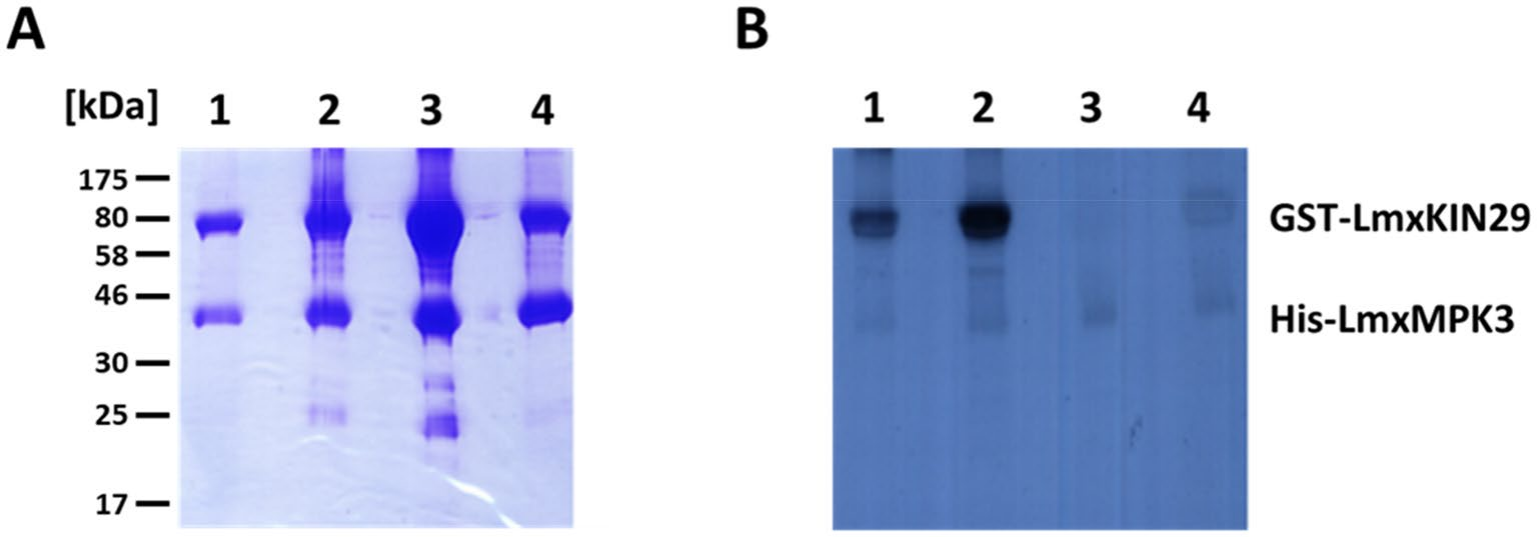
Determination of the LmxMPK3 LmxKIN29 phosphorylation site. (**A**) radiometric kinase assay of activated His-LmxMPK3 with different GST-LmxKIN29 proteins resolved on 14% SDS-PAGE and Coomassie-stained**;** (**B**) autoradiograph after 24 h of exposure. Lane 1, His-LmxMPK3 + GST-LmxKIN29; lane 2, His-LmxMPK3 + GST-LmxKIN29S551A; lane 3, His-LmxMPK3 + GST-LmxKIN29A2; lane 4, His-LmxMPK3 + GST-LmxKIN29S554A; M, marker in kDa.

In order to determine the phosphorylation site(s) used by LmxMPK3, full-length GST-LmxKIN29 fusion proteins of wild type LmxKIN29 and three phosphorylation site mutants were generated replacing serine by alanine. The corresponding expression plasmids were pGEX-KGSPKin29, pGEX-KGSPKin29S551A, pGEX-KGSPKin29A2 (S551A/S554A), and pGEX-KGSPKin29S554A all leading to expression of a 95.5 kDa fusion protein (27.3 kDa GST + 68.2 kDa LmxKIN29) in *E. coli* (Fig. 2). In addition, the hexahistidine-tagged, activated MAP kinase His-LmxMPK3 was expressed and purified from *E. coli*. The predicted size of His-LmxMPK3 is 45.8 kDa (2.1 kDa hexahistidine tag + 43.7 kDa LmxMPK3; Fig. 2). The purified proteins were used in protein kinase assays to determine the ability of activated His-LmxMPK3 to phosphorylate GST-LmxKIN29 proteins. GST-LmxKIN29 showed a strong phosphorylation signal with His-LmxMPK3 (Fig. 2, lane 1). His-LmxMPK3 also phosphorylated LmxKIN29S551A indicating that serine 551 is not used as a single phosphorylation site by His-LmxMPK3 (Fig. 2, lane 2). However, LmxKIN29A2 was not phoshorylated (Fig. 2, lane 3). GST-LmxKIN29A2 is devoid in serine 551 and serine 554 suggesting that threonine 440 and serine 548 are not phosphorylated by His-LmxMPK3, leaving serine 551 and 554 as phosphorylation sites for LmxMPK3. To distinguish between these two sites, the mutant LmxKIN29S554A carrying the single serine 554 to alanine mutation was tested and found to be not phosphorylated by activated His-LmxMPK3 (Fig. 2, lane 4) indicating that serine 554 is the sole phosphorylation site used by this kinase in vitro. However, the phosphorylation assay only determines incorporation of radiation into the kinase substrate without providing information about the actual phosphorylation site. Therefore, it is possible that for wild type LmxKIN29 His-LmxMPK3 first phosphorylates serine 554 followed by phosphorylation of serine 551 similar to the sequential phosphorylation of the TxY motif in a MAP kinase by its activating MAP kinase kinase^24^. Alternatively, serine 548 or serine 551 could be phosphorylated in vivo by additional kinases adding to the complexity of regulation involving integration of signals.

### Generation of LmxKIN29 deletion mutants

**Figure 3:**
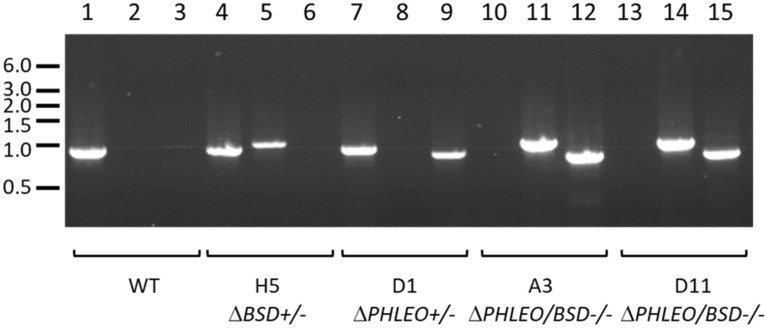
PCR to confirm deletion of LmxKIN29. Lanes 1, 4, 7, 10, and 13, detection of *LmxKIN29* by an 860 bp PCR fragment; lanes 2, 5, 8, 11 and 14, detection of correct integration of *BSD* by a 937 bp PCR fragment; lanes 3, 6, 9, 12, and 15, detection of correct integration of *PHLEO* by a 766 bp PCR fragment. Marker in kb.

To assess the function of LmxKIN29 in *L. mexicana* the generation of a null mutant using homologous recombination was attempted. Knockout cassettes containing phleomycin binding protein (*PHLEO*) and blasticidin-S deaminase (*BSD*) resistance marker genes were sequentially introduced into wild type *L. mexicana* promastigotes. To confirm the correct gene replacement in the knockout mutants, diagnostic PCRs to show successful replacement of *LmxKIN29* by either of the resistance marker genes were performed (Fig. 3). An 860 bp PCR fragment indicating the presence of at least one allele of *LmxKIN29* was detected in the wild type (Fig. 3; lane 1) and the single allele knockout clones H5 and D1 (Fig. 3; lanes 4 and 7). A 937 bp PCR fragment indicating the correct integration of the blasticidin resistance gene (*BSD*) could be amplified from the single allele knockout clone H5 and both double allele knockout clones A3 and D11 (Fig. 3; lanes 5, 11 and 14). Moreover, a 766 bp PCR fragment indicating replacement of LmxKIN29 by the phleomycin resistance gene (*PHLEO*) was generated from DNA of the single allele knockout clone D1 and both double allele knockout clones A3 and D11 (Fig. 3; lanes 9, 12 and 15). The absence of a PCR fragment for *LmxKIN29* and the presence of fragments for both resistance genes confirmed that independent null mutants were successfully generated.

### Phenotypic analysis of the LmxKIN29 null mutant

**Figure 4:**
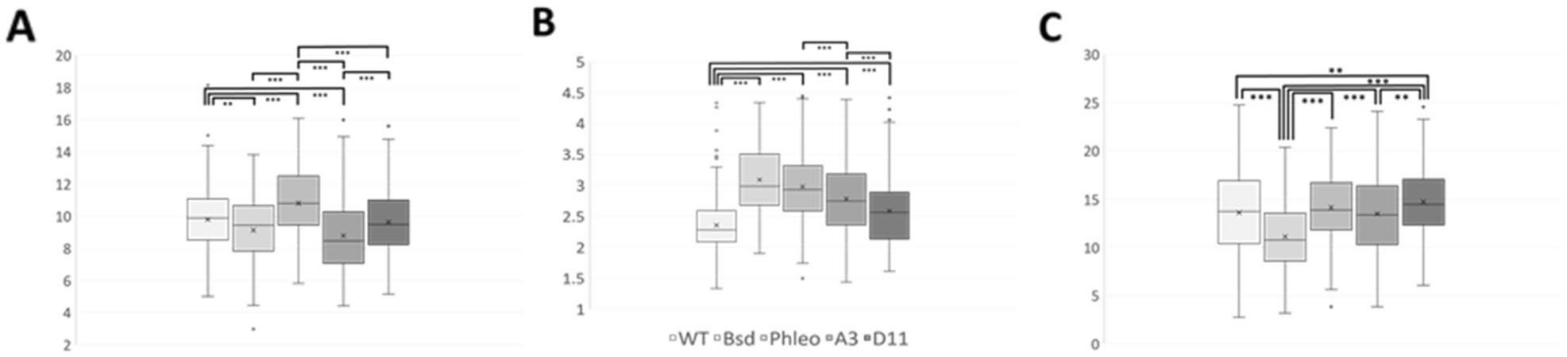
Morphological analysis of promastigotes of LmxKIN29 deletion mutants in *L. mexicana.* (**A**) cell body length in µm; (**B**) cell body width in µm; (**C**) flagellum length in µm. Boxplot of distribution of measurements. Different shades of grey from left to right indicate results for wild type (WT) *L. mexicana*, single allele knockout with *BSD* (*BSD*), single allele knockout with *PHLEO* (*PHLEO*), two null mutants with *BSD* and *PHLEO*, A3 and D11. × , means. Stars indicate significant differences (p** < 0.01, p*** < 0.001) (Student’s t-Test). Measurements of 189 random cells were taken using Image J.

Microscopic examination of 189 cells from Δ*LmxKIN29* single allele and null mutant promastigotes in logarithmic growth phase revealed no obvious differences compared to wild type promastigotes in cell body length, cell body width and flagellum length (Fig. 4). Normal distribution was confirmed by an F-test and graphic analysis of the normal distribution using Microsoft Excel. As expected for a normal distribution the mean and median for all data was very similar. Having established normal distribution, the variance of the data was determined using an F-test. According to the outcome of the F-test, data were further analysed by the relevant two-tailed, non-paired Student’s t-Test to determine whether the means were significantly different. The average cell body length of the wild type was significantly longer than the cell body length of one of the single allele knockouts (*BSD*) and of one of the null mutants (A3), not showing any difference compared to the second null mutant (D11) and being significantly shorter than that of the second single-allele knockout (*PHLEO*). Moreover, significant differences in average cell body length were observed between the two single allele knockouts and between the two null mutants. This variability led to the conclusion that cell body length is not influenced by LmxKIN29. Average cell body width was found to be smallest for the wild type promastigotes (p < 0.001), which coincidentally showed the highest cell density of 5.4 × 10^7^ cells/mL. This could indicate that the culture was about to reach late-log phase and stationary growth phase, which is characterised by the appearance of metacyclic promastigotes with shorter and slimmer cell bodies along with elongated flagella. The on average widest cells were found in one of the single allele deletion mutants (*BSD*) with the lowest cell density of 3.5 × 10^7^ cells/mL indicating a rapidly dividing population. The second single allele deletion mutant (*PHLEO*) does not follow the expected pattern and shows an average cell body width that is bigger than expected for the cell density of 5.1 × 10^7^ cells/mL. However, the two null mutants fit the expected pattern with A3 showing on average wider cells at a cell density of 3.6 × 10^7^ cells/mL than D11, which had a cell density of 4.5 × 10^7^ cells/mL. The observed variation in measurements led to the conclusion that LmxKIN29 also has no effect on cell body width. The longest average length flagella were found in one of the null mutants (D11) (p < 0.01), which also showed higher cell density compared to the second null mutant (A3). A3 showed no significant difference of average flagellar length compared to the wild type, however, a lower range in flagellar lengths was observed. Finally, as expected, the cells with the lowest cell density, the single allele knockout *BSD*, also showed the shortest average flagellum length. Overall, none of the measured features was consistently affected by the deletion of *LmxKIN29* in both single allele knockouts or both null mutants and the observed differences are therefore likely due to variations in cell densities and might represent cells in various stages of the cell cycle^25^. It has been shown that the length of *Leishmania* flagella extends over multiple cell division cycles, growing progressively longer with each cycle^25^. Alternatively, there could be another kinesin present in promastigotes, which could maintain the morphology of the *LmxKIN29* null mutants through functional redundancy^26^. A knockout of the kinesin TbKif13-2, which had been localised to the flagellum, showed no significant elongation of the flagellum and overexpression only slightly decreased flagellar length and the rate of growth of a new flagellum during cell division^7^. Localisation of LmxKIN29 in promastigotes could possibly help to inform about its role in the parasite. The absence of LmxKIN29 did not cause defects in flagellum length despite the fact that LmxMPK3 can phosphorylate LmxKIN29. Hence, LmxMPK3 might not only be involved in flagellum length regulation but has additional yet unknown functions involving LmxKIN29 in promastigotes grown in vitro. Further analyses are required to show in vivo interaction of the two proteins or alternatively to prove the absence of phosphorylation on S554 in the LmxMPK3 null mutant. With no apparent function in the promastigote stage, LmxKIN29 might play a role in the mammalian amastigote stage justifying an in vivo investigation.

### Generation of genomic add-backs to complement Δ*LmxKIN29−/−*

**Figure 5:**
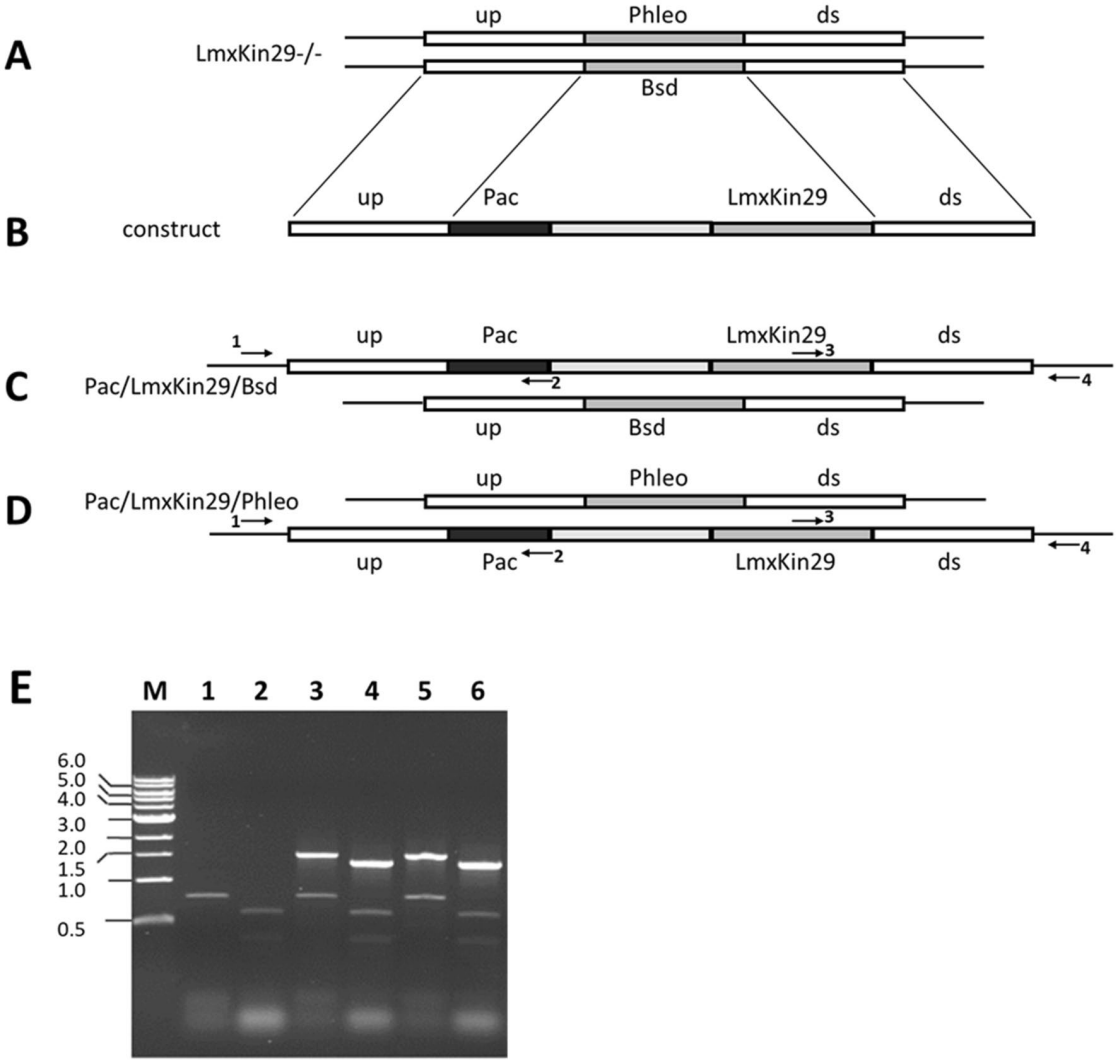
LmxKIN29 genomic add-back. (**A**) genomic situation in Δ*LmxKIN29*-/- null mutant. (**B**) *LmxKIN29* add-back construct; (**C**) add-back replacing *PHLEO*; (**D**) add-back replacing *BSD*; (**E**) PCR to prove correct replacement. Lanes 1 and 2, null mutant A3; lanes 3 and 4, *LmxKIN29* add-back A3E2; lanes 5 and 6, *LmxKIN29* add-back D11H2; lanes 1, 3, and 5, primer pair to amplify a 1481 bp DNA fragment showing correct integration of 3’-end of *PACLmxKIN29* construct; lanes 2, 4, and 6 primer pair to amplify 1308 bp fragment indicating correct integration of 5’-end of *PACLmxKIN29*; all lanes including lanes for the null mutants show amplification of either one or two additional unspecific bands between 400–800 bp, which serve as internal controls. M, DNA marker in kb.

The add-back construct pBPACLmxKIN29 was generated carrying the puromycin acetyl transferase resistance gene (*PAC*) and *LmxKIN29* wild type gene. Puromycin-resistant clones were generated by integration of the cassette into the genomic DNA (Fig. 5A–D) replacing one of the resistance markers *BSD* or *PHLEO* by homologous recombination. The 1481 bp DNA fragment obtained by PCR indicated the presence of *LmxKIN29*, whereas the 1308 bp DNA fragment showed correct integration of the *PAC/LmxKIN29* carrying construct into the *LmxKIN29* gene locus in all tested clones (A3E2 and D11H2) (Fig. 5E).

### Mouse infection studies with wild type, *LmxKIN29* deletion mutants, and add-back mutants of *L. mexicana*

**Figure 6:**
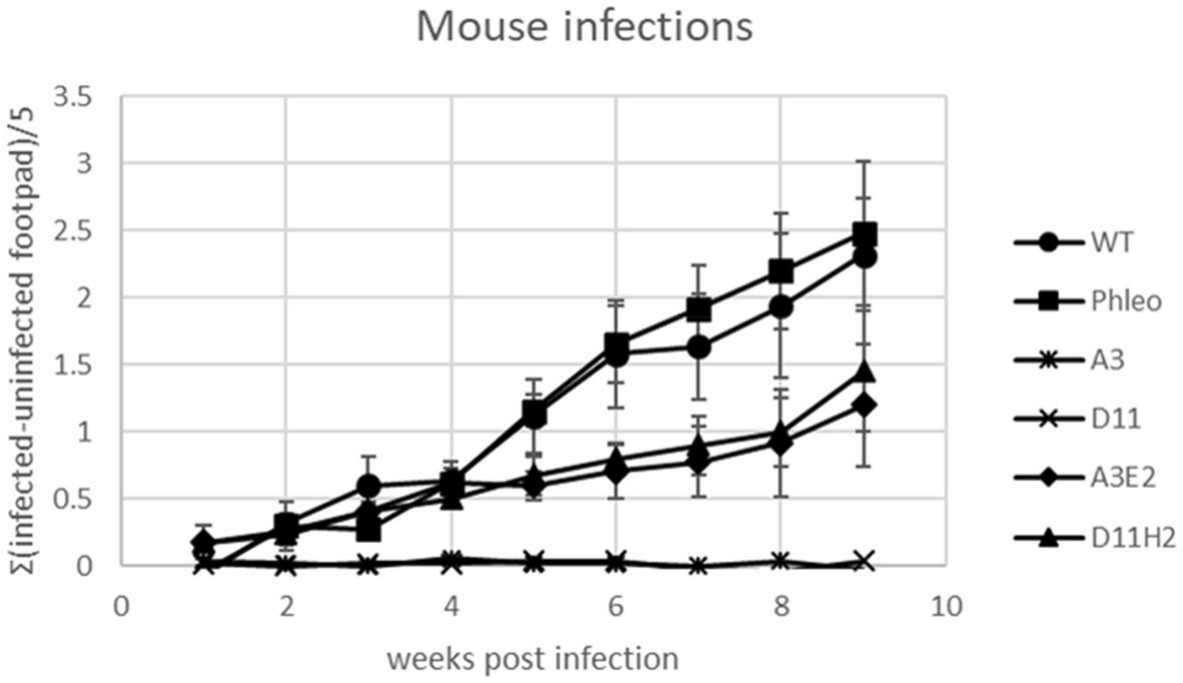
Footpad infection of female Balb/c mice with wild type, single-allele knockout, double-allele knockout, and add-back clones. WT, *L. mexicana* wild type; Phleo, Δ*LmxKIN29+/-* single-allele knockout; A3 and D11, independet Δ*LmxKIN29*-/- double allele knockout clones; A3E2, add-back clone for A3; D11H2, add-back clone for D11.

Late log-phase *L. mexicana* promastigotes were injected into the left hind footpad of five female Balb/c mice. The infected footpad was measured every week and compared to the non-infected one (Fig. 6). *L. mexicana* wild type, single allele knockout and genomic add-back caused infection and the footpad swelling increased over eight weeks. No lesion development could be observed in mice infected with either of the null mutant clones (A3, D11). Mice infected with the single allele deletion mutant or any of the *LmxKIN29* add-back clones (A3E2, D11H2) showed no significant difference in lesion development compared to the wild type. The attempt to grow parasites from the area of infection was successful from mice showing clear lesion development, but failed for the null mutants Δ*LmxKIN29*-/-, suggesting that the null mutant parasites did not survive in the mouse, corroborating LmxKIN29 as a suitable drug target.

### Localisation of LmxKIN29

**Figure 7:**
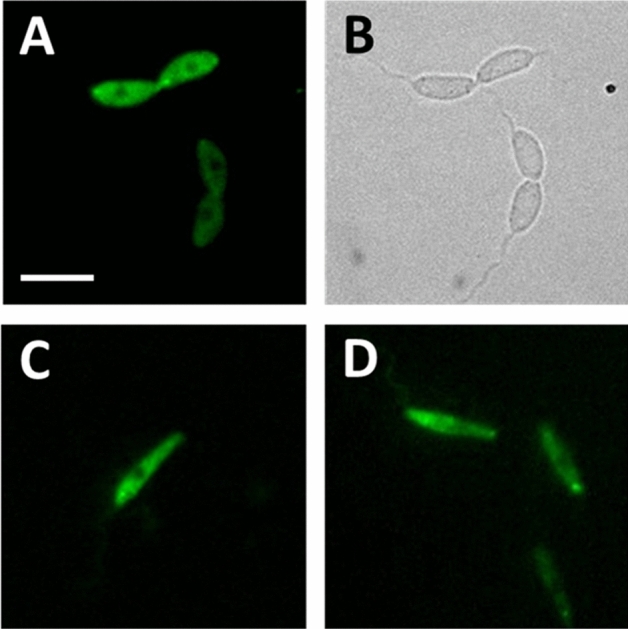
Fluorescence microscopy of LmxKIN29 in live promastigotes. (**A**, **B**) *L. mexicana* promastigotes carrying pTHKin29GFP clone A3C12 (GFP fused to C-terminus); (**C**, **D**) *L. mexicana* promastigotes carrying pTHGFPKin29, D11C2 (GFP fused to N-terminus). Bar, 10 μm.

**Figure 8:**
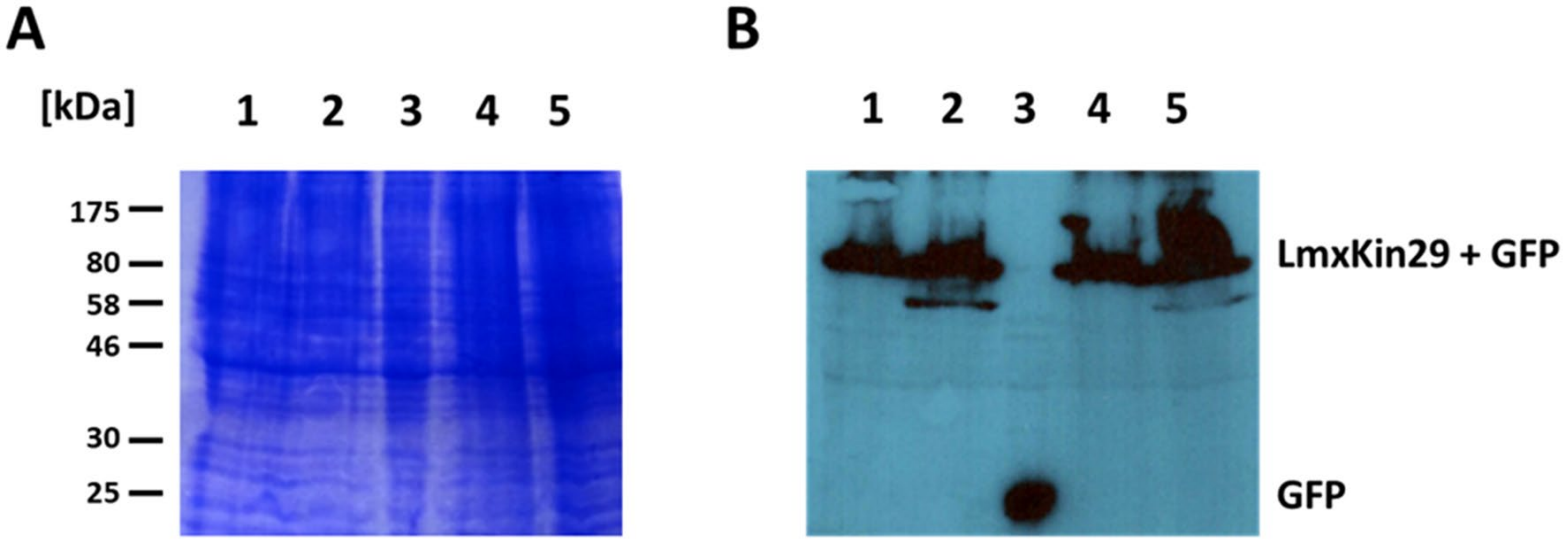
Expression of GFP-tagged LmxKIN29 in *L. mexicana* promastigotes. **(A**) 14% Coomassie-stained SDS-PAGE; (**B**) immunoblot using an anti-GFP antibody. Lanes 1 and 2, pTHGFPLmxKIN29 A3C10 and D11C2, respectively (N-terminal GFP); lane 3, *L. mexicana* expressing GFP only; lanes 4 and 5, pTHKin29GFP A3C12 and D11A1, respectively (C-terminal GFP). Marker in kDa.

*LmxKIN29* was expressed in promastigotes of the two null mutant clones by introducing pTHGFPLmxKIN29 where GFP is fused to the N-terminus of LmxKIN29 and pTHLmxKIN29GFP with a C-terminal fusion. Fluorescence microscopy of live *L. mexicana* promastigotes expressing LmxKIN29GFP or GFPLmxKIN29 showed localisation of LmxKIN29 throughout the cytosol with an accumulation next to the flagellar pocket (Fig. 7). Localisation was similar in most cells with some dividing cells showing an accumulation of LmxKIN29 at the anterior and posterior ends. Interestingly, a fluorescent signal could be seen in the area where dividing cells were still attached to each other for both LmxKIN29GFP and GFPLmxKIN29 (Fig. 7A). The presence of the intact GFP-tagged LmxKIN29 was validated by immunoblot analysis with an anti-GFP antibody (Fig. 8). Cell lysates of two clones carrying pTHGFPKin29 (A3C10 and D11C2), two clones with pTHKin29GFP (A3C12 and D11A1) and a cell line expressing GFP only were analysed. Expected band sizes for LmxKIN29 fused to GFP at 95.1 kDa (68.3 kDa for LmxKIN29 + 26.8 kDa for GFP) were found for all clones. No free GFP was detectable in any of the clones expressing GFP-tagged LmxKIN29 when compared to the band in the cell lysate of cells expressing GFP only (Fig. 8, lane 3). This confirms that the fluorescence seen in the promastigotes reflects the localisation of GFP-tagged LmxKIN29 and not GFP alone.

## Conclusion

LmxKIN29 is a typical kinesin motor protein containing the three main parts, motor domain, coiled-coil stalk and tail domain. Based on its primary sequence LmxKIN29 has been classified as a member of the kinetoplastida orphan kinesin superfamily^6^. It is phosphorylated in promastigotes and amastigotes and the MAP kinase LmxMPK3 was found to phosphorylate LmxKIN29 on serine 554. *LmxKIN29* null mutant clones did not show any overt phenotypic difference compared to *L. mexicana* wild type promastigotes and the protein is therefore not essential for flagellum assembly. However, LmxKIN29 is required for *L. mexicana* survival in the mammalian host because *LmxKIN29* null mutants were unable to cause lesions in infected Balb/c mice and no parasites could be detected at the injection site ten weeks post infection. Being an orphan kinesin and diverse in sequence suggests that it should be possible to identify a specific inhibitor not affecting a mammalian host kinesin. A specific drug able to inactivate LmxKIN29 could therefore be explored as a viable strategy against leishmaniasis.

## Material and methods

### Ethics statement

Animals were maintained and handled at the Strathclyde Institute of Pharmacy and Biomedical Sciences (SIPBS) of the University of Strathclyde in accordance with institutional guidelines and UK national legislation (Animals Scientific Procedures Act 1986). All experiments were approved by the University of Strathclyde Ethics Committee and performed under the Home Office license (PPL PF669CAE8). All methods involving the use of animals are reported in accordance with ARRIVE guidelines (https://arriveguidelines.org) for the reporting of animal experiments.

### Generation of DNA constructs

The Expand High Fidelity PCR System (Roche) was used for the generation of all DNA fragments for cloning. PCRs were performed using genomic DNA from *L. mexicana* prepared using the method described before^27^. *LmxKIN29* was cloned from genomic DNA of *L. mexicana* using three PCR reactions to amplify an N-terminal coding PCR-fragment of 1095 bp introducing EcoRI, BamHI and PciI sites at the 5’-end and NheI, EcoRV and HindIII sites at the 3’-end of the sequence using the oligos 5’- CGC**GAATTC**GG**GGATCC**GA**ACATGT**CACGCATACAGAAAAGC-3’ and 5’-GCG**AAGCTTGATATCGCTAGC**GCGCTGCCCAAAAAAGAGGGTG-3’ and two C-terminal coding PCR-fragments of 804 bp and 825 bp introducing EcoRI and NheI sites at the 5’-end and HpaI and HindIII sites at the 3’-end of the first sequence with the oligos 5’-CGC**GAATTCGCTAGC**CAAATCAAGCAGGATGCG-3’ and 5’-GCG**AAGCTTGTTAAC**TCACGCCGCCTTGCGCTGCGCAGCT-3’ and an additional EcoRV site and the epitope for the monoclonal antibody mAb LT8.2^28^ in the second sequence with the alternative reverse oligo 5’-GCG**AAGCTT**TCA**GATATC**GACGCGCGGCGACCA**GTTAAC**CGCCGCCTTGCGCTGCGCAGCT-3’. All fragments were cleaved with EcoRI and HindIII and cloned into pBSKII(+) linearised with the same enzymes. The inserts of the resulting plasmids were sequenced. All three plasmids were cleaved with NheI and HindIII and the resulting 4020 bp fragment was ligated to either the 786 bp or 807 bp fragment to generate the plasmids pBHNKin29 carrying full length *LmxKIN29* or pBHNKin29LT8.2 carrying *LmxKIN29* plus the sequence coding for the mAb LT8.2 epitope as a C-terminal tag.

For expression as a GFP-fusion protein in *Leishmania*, a 1853 bp EcoRI/HpaI fragment from pBHNKin29 was cloned into pTH6cGFPn linearised with MfeI and HpaI for an N-terminal GFP; a 1871 bp EcoRI/EcoRV fragment from pBHNKin29LT8.2 was cloned into pTH6nGFPc linearised with MfeI and HpaI for a C-terminal GFP after the mAb LT8.2 epitope^29^.

The bacterial expression plasmid pGEX-KG^30^ was cleaved with SmaI and BamHI, the ends filled-in and re-ligated to generate pGEX-KGSP lacking a proline residue in the linker beween the glutathione-S-transferase gene and the multicloning site removing a putative MAP kinase phosphorylation motif (serine followed py proline or SP-site). For expression as a GST-fusion protein in *Escherichia coli*, pBHNKin29 was cleaved with PciI and HindIII and the resulting 1841 bp fragment was ligated into pGEX-KGSP linearised with NcoI and HindIII resulting in pGEX-KGSPKin29. This plasmid was used to introduce different mutations in the putative phosphorylation sites by replacement of various DNA fragments. A recoded part of *LmxKIN29* spanning the sequence from the AfeI site located at position 1167 and the SphI site at position 1792 of *LmxKIN29* was synthesised for replacement containing a serine 551 to alanine mutation and additional KpnI and NruI sites for identification. A 255 bp DNA sequence flanked by AflII and HindIII sites was synthesised containing serine 551 and serine 554 to alanine mutations. The introduction of a KpnI site allowed identification of the desired plasmid. Finally, a serine 554 to alanine mutation was generated by synthesising another 255 bp DNA sequence flanked by AflII and HindIII sites containing the serine 554 to alanine mutation and an AscI site allowing to identify the insert by restriction analysis.

For the deletion constructs, 679 bp of the *LmxKIN29* upstream region and 627 bp of its downstream region were amplified by PCR using genomic DNA from *L. mexicana* and the oligonucleotide pairs 5’-**GATATC**CGCGCACCCATAGCC**ACATGT**GTGCATCTCTCC-3’ and 5’-TTGTCGTCTCACTTCATCG**CCATGGCCTAGGAGATCT**CGAG-3’, and 5’-**CCTAGG**GCTAGCTTGTTCGGACACTGCGATATGCCGGACC-3’ and 5’-CCATGACCATCTGCGAGTCACGTTGTGGTGATATCTCGAG-3’ and the resulting fragments cloned into pGEM®-T Easy (Promega) and sequenced. Using AvrII and NdeI the two plasmids were combined into one construct containing the upstream region of *LmxKIN29* separated by NcoI, AvrII and NheI restriction sites from its downstream region. The pGEM®-T Easy backbone was replaced by pBSKII(+) using EcoRV resulting in pBupKin29ds. The resistance marker gene *PHLEO* was amplified from pX63polPhleo^31^ using the oligonucleotides 5’-AGATCA**CCATGG**CCAAGTTGACCAGT-3’ and 5’-CGG**CCTAGG**TCAGTCCTGCTCCTCGGC-3’ to introduce NcoI and AvrII restriction sites, cloned into pCR2.1 (Thermo Fisher Scientific) to generate pCR2.1phleo and sequenced. Using NcoI and AvrII on pCR2.1phleo and NcoI and NheI on pEX-A2-BLA-ALA, containing a newly synthesised, recoded blasticidin-S-deaminase gene (*BSD*), the resistance marker genes were cloned into pBupKin29ds via NcoI and NheI restriction sites resulting in pBKin29upBsdds and pBKin29upPhleods. DNA fragments containing the *LmxKIN29* upstream region, a resistance marker gene and the corresponding downstream region were liberated from these plasmids using EcoRV, gel-purified and used for transfection of *L. mexicana* (see below).

For the generation of a genomic add-back construct, a 5852 bp EcoRV/HpaI DNA fragment was isolated from pX14polNcoIPac^32^. pBHNKin29 (see above) was cleaved with BamHI and HpaI to liberate a 1845 bp DNA fragment, which was ligated with the 5852 bp DNA fragment to generate pX14polNcoIPACKin29. This plasmid was amplified in a Dam-methylase negative strain of *E. coli* to allow it to be cleaved with XbaI and NcoI to generate a 1245 bp NcoI/XbaI DNA fragment carrying a partial *LmxKIN29* and a 2626 bp NcoI/NcoI DNA fragment carrying *PAC*, the *DHFR-TS* intergenic region (IR) and the remainder of *LmxKIN29*. The first DNA fragment was ligated into pBupKin29ds (see above) linearised with NcoI and NheI followed by the ligation of the second DNA fragment into the NcoI site of the intermediate construct to generate the genomic add-back construct pBPACLmxKIN29. A 5138 bp EcoRV DNA fragment carrying the *LmxKIN29* upstream region, *PAC*, *DHFR-TS*-IR, *LmxKIN29*, and the *LmxKIN29* downstream region was isolated and used for transfection (see below).

### Expression and purification of recombinant proteins

Hexahistidine-tagged LmxMPK3 was co-expressed with a constitutively active MAP kinase kinase, LmxMKK(D)^33^ and purified using Co^2+^-sepharose as described before^32^. Different versions of *LmxKIN29* were expressed as glutathione S-transferase (GST) fusion proteins using pGEX-KGSP producing recombinant fusion proteins with the GST-tag located at the N-terminus. Expression of the glutathione S-transferase (GST) fusion proteins was achieved by induction of bacterial cultures grown to an optical density at 600 nm of 0.8 in Luria–Bertani medium with 100 µM IPTG (isopropyl-β-D-thiogalactopyranoside) overnight at 30 °C in a shaking incubator. Bacteria were washed once in cold phosphate-buffered saline (PBS) (137 mM NaCl, 2.7 mM KCl, 10 mM Na_2_HPO_4_, 1.8 mM KH_2_PO_4_) and resuspended in 50 µl of cold PBS per mL of the original culture volume. The suspension was subjected to sonication on ice with a Branson Sonifier 250 apparatus in pulse mode followed by the addition of Triton X-100 to a concentration of 1%. Solubilisation of proteins occurred by end-over-end rotation of the lysate at 4 °C for 30 min. Finally, the solution was centrifuged at 4 °C and 12,000 × g for 10 min and the supernatant was collected for purification of the protein on Amintra® Glutathione Affinity Resin following the instructions of the manufacturer (Expedeon).

### Kinase reaction

GST-LmxKIN29 from the protein expression was left bound to Amintra beads and used with 2–5 µg/µL purified His-LmxMPK3 in 50 mM 3-(N-morpholino)propanesulfonic acid (MOPS) pH 6.5, 100 mM NaCl, 10 mM MnCl_2_ and 50 µM [γ-^32^P]ATP (500 cpm/pmol) in 50 µL. All reaction tubes were end-over-end rotated at 27 °C for one hour. 12.5 μL of 5 × SSB (2% SDS, 20% glycerol, 0.001% bromophenol blue, 200 mM DTT and 62.5 mM Tris–HCl pH 6.8) were added, the sample heated at 95 °C for 10 min and resolved on SDS-PAGE. The gel was Coomassie-stained, dried and exposed to X-ray films at − 80 °C.

### Culturing of *L. mexicana* promastigotes

Promastigotes were grown in SDM-79 medium^34^ containing 10% heat-inactivated fetal calf serum and 7.5 μg/mL hemin at 27 °C. Antibiotics (InvivoGen) were added, if required, at the following concentrations: Blasticidin (Bsd) (5 μg/mL), phleomycin (Phleo) (5 μg/mL), hygromycin B (20 μg/mL), puromycin (20 µg/mL).

### Fluorescence microscopy

Log-phase *Leishmania* promastigotes in media were cooled on ice for 30 min. 1.5 µL of the live parasite suspension trapped between a glass slide and cover slip was examined using a Nikon Eclipse E600 epifluorescence microscope equipped with a Hamamatsu Orca-285 camera and an in-house capturing software (Winfluor, John Dempster, SIPBS, University of Strathclyde). GFP fluorescence was observed with a 60× oil-immersion objective using a FITC filter cube. Images were typically taken with an exposure time of 300 ms; brightfield images were taken using 15 ms exposure time.

### Cell measurements

Log-phase *Leishmania* promastigotes were fixed in PBS/formaldehyde (9:1) and examined at 40× magnification using light microscopy. Up to 15 fields of view were randomly captured with a GXCAM camera using GXCapture software. Flagellar length, cell length and width of approximately 200 cells of each cell line were measured tracing the flagellum with the freehand tool or using a straight line of Image J Version 1.51p.

### Transfection of *L. mexicana*

Transfections were performed using a human T-cell Amaxa nucleofector kit following the manufacturer’s instructions. 3 × 10^7^ late log-phase promastigotes were harvested by centrifugation for 2 min at 5600×*g* at 4 °C and the cells resuspended in 100 μL supplemented electroporation buffer with 1–5 μg of DNA fragment or plasmid and electroporated using the programme V-033, followed by incubation on ice for 10 min. After transfer into 10 mL SDM-79 medium and incubation for 24 h at 27 °C without selection the whole culture was diluted 1:2 and 1:40 resulting in 20 mL of each dilution, relevant antibiotics were added and the cell suspensions aliquoted in 200 μL per well on 96-well plates, which were incubated at 27 °C for 10–14 days.

### PCR analyses

All diagnostic PCRs were performed using MyTaq polymerase following the instructions of the manufacturer (Meridian Bioscience). The oligonucleotides 5’-GGCAGTCGCGTAGTACTGGC-3’ and 5’-GCCTGACTTGCGGGTCACGG-3’ were used to detect the presence of *LmxKIN29* as an 860 bp DNA fragment. Replacement of *LmxKIN29* by resistance marker genes was tested using the forward oligo 5’-GGCAGTCGCGTAGTACTGGC-3’ with the reverse oligo 5’-AACTCGACCGCTCCGGCGACG-3’ for the gene encoding the phleomycin-binding protein (*PHLEO*) resulting in a 766 bp DNA fragment and 5’-ATCGCGACGATACAAGTCAGG-3’ for the gene encoding the blasticidin-S deaminase (*BSD*) generating a 937 bp DNA fragment.

Genomic add-backs were tested using the oligonucleotides 5’-GGCAGTCGCGTAGTACTGGC-3’ and 5’-CGTCCTAGGCACCGGGCTTGCGGGTCATGC-3’ to generate a 1308 bp PCR fragment indicating correct integration at the 5’-end of the add-back fragment and 5’-CAACATCAGCCCTTCCGAT-3’ and 5’-CGAGGTGCACCTTCGCGAC-3’ to generate a 1481 bp PCR fragment for the 3’-end of the correctly integrated *PACKin29* fragment.

#### Immunoblot analysis

Cell lysates at a concentration of 1 × 10^8^ cells/mL were prepared using RIPA buffer (25 mM Tris–HCl pH 7.6, 150 mM NaCl, 1% NP-40, 1% sodium deoxycholate, 0.1% SDS). 2 × 10^7^ cells were resolved on SDS-PAGE and blotted to a Polyvinylidene Difluoride membrane. The membrane was incubated for one hour at 37 °C in blocking solution (PBS, 5% (w/v) low-fat dried milk powder, 20 mM Tris–HCl pH 7.5, 0.2% Tween-20) before being incubated overnight at 4 °C with primary antibody in blocking solution. After four washes with PBST (PBS with 0.2% Tween-20) at room temperature the membrane was incubated for one hour at 37 °C with the secondary antibody in blocking buffer. Finally, the membrane was washed three times in PBST, twice in PBS and developed by chemiluminescence detection using the Supersignal system (Thermo Fisher Scientific) and exposure to X-ray film.

#### Mouse infection

The *L. mexicana* Balb/c mouse infection model is well established and produces a reproducible outcome in form of development of a swelling/lesion at the injection site. The preferred site of infection is the hind foot as it allows easy measurement of the swelling over time. The rate at which the swelling occurs at a given dose of late log-phase promastigotes is consistent and known. Variation between infected mice occurs due to slight differences in the volume of the inoculum at a high cell density of 3.3 × 10^8^ cells/mL. A sample size of five animals per group allows to adjust for these differences and the rare sudden death of an animal unrelated to the infection. The purpose of the experiment is to assess the ability of genetically modified *L. mexicana* promastigotes to cause the disease in the infected mouse. The possible outcomes are no difference in foot swelling compared to infection by the wild type, delayed onset and/or slow progression of swelling, or no swelling at all. Measurements are non-invasive and have no impact on the swelling. All experimental groups were clearly and visibly labelled and known to the person performing the measurements. All footpad infection studies were conducted with female 8–12 weeks old Balb/c mice (20–25 g) bred and supplied in-house. Mice were maintained in groups of five in cages with sawdust as a substrate, a red hut, a sizzle nest, an aspen chew stick and food (SDS CRM(p)) and water ad libitum, and a 12 h light/12 h dark photoperiod in rooms maintained at 45–65% humidity, 20–21 °C, 16/20 air changes per hour. A total of 30 mice were used with 5 mice per group allowing statistical analysis. All animals infected were used in the data analysis. The parental *L. mexicana* strain was used as the positive control. A single allele deletion mutant from which the two null mutants are derived was used to prove infectivity is not affected by gene dosage. Two independent null mutant clones were used to show that deletion of both alleles consistently leads to the same infection phenotype. Each null mutant was complemented by reintegration of the wild type *LmxKIN29*  gene to prove that infectivity could be restored independently. Late log-phase (3–4 × 10^7^ cells/mL) *L. mexicana* promastigotes were harvested by centrifugation at 5,600 × g for 2 min, washed with ice-cold PBS and resuspended in PBS to a final density of 3.3 × 10^8^ cells/mL. Each mouse was injected s.c. into the left hind footpad with 30 μL of the *Leishmania* cell suspension, equalling 1 × 10^7^ cells. The infection was monitored by weekly measurement of both hind feet using a calliper gauge. The general health of the infected mice was monitored by taking their weight weekly. Mice were also regularly examined to detect cutaneous ulcers and secondary lesions. The experiment was terminated once the first mouse had reached their clinical endpoint (5 mm diameter of the foot measured sole to top). A tissue sample was taken from each injected foot post mortem and incubated in SDM-79 medium in an attempt to recover parasites.

## Supplementary Information

Supplementary Information.
