# Supplementary material for: Absence of DEATH kinesin is fatal for Leishmania mexicana amastigotes
Source: Sci Rep. 2022 Feb 28;12:3266. doi: 10.1038/s41598-022-07412-z (PMC8885694; doi:10.1038/s41598-022-07412-z)
Supplement: Supplementary file 1 — Supplementary Information. [file 41598_2022_7412_MOESM1_ESM.pdf]

Supplementary information:

Figure S1:

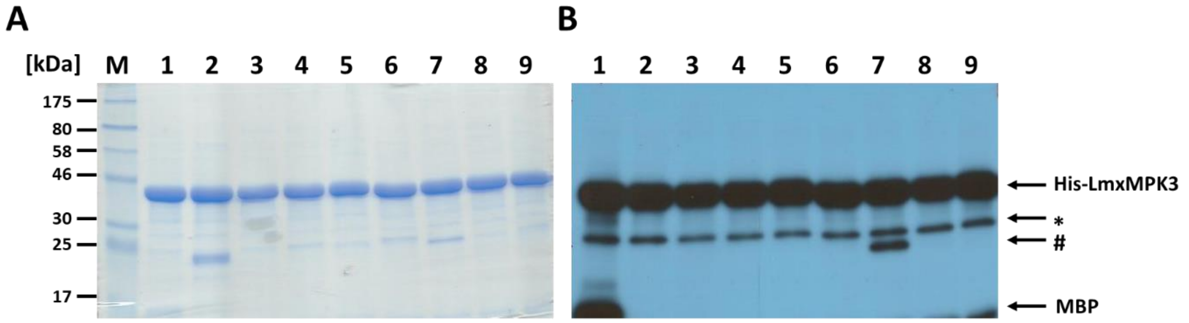

Figure S1: Phosphorylation of GST-LmxKIN29peptide fusion using activated His-LmxMPK3. Left, Coomassie-stained 12% SDS-PAGE; right, autoradiograph after 15 hours exposure. Lane 1, myelin basic protein (MBP); lane 2, GST; lanes 3-6, 8, 9, unsequenced GST-peptide fusions; lane 7, GST-LmxKin29peptide (#). (*), either protein of bacterial origin phosphorylated by His-LmxMPK3 or degradation product of autophosphorylated His-LmxMPK3; M, marker in kDa.

Figure S2:

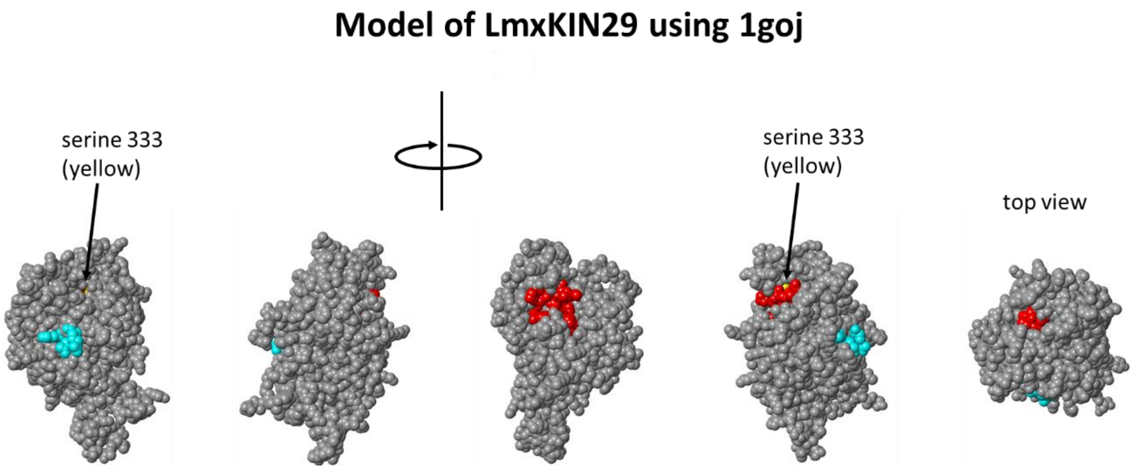

Figure S2: Views of LmxKIN29 from top and four sides. Red, ATP-binding site; cyan, microtubule-binding site; yellow, S333. Images were prepared using Jmol FirstGlance 3.0 (http://firstglance.jmol.org).

Figure S3:

```
Lmex      MSRIQKSSKSAPKTISVYCRVRPPVPQEKGHNFNNIV-YDDADNRTITVTRKSGSKSFEK      59
rat       ------MADPAECSIKVMCRFRPLNEAEILRGDKFIPKFKGEE--TVVI-------GQGK      45
Nc        -------MSSSANSIKVVARFRPQNRVEIESGGQPIVTFQGPD--TCTV----DSKEAQG      47
                  . :  :*.* .*.**    *   . : *  :.. :  *: :
                         BBBBB     HHHH         BBB    BBBB
                           β1  L1   α0   L1     β1a    β1b         L2

Lmex      RYFFNRVFRPTVTQKDVYETFAKNAVDAAFDGQHGVLFVYGQTGSGKTFTIS---NDDPK      116
rat       PYVFDRVLPPNTTQEQVYNACAKQIVKDVLEGYNGTIFAYGQTSSGKTHTMEG-KLHDPQ      104
Nc        SFTFDRVFDMSCKQSDIFDFSIKPTVDDILNGYNGTVFAYGQTGAGKSYTMMGTSIDDPD      107
           : *:**:  .  .*.::::   *  *.  ::* :*.:*.****.:**:.*:     .**.
          BBB   BB     HHHHHHHHHHHHHHHHH    BBBBBB      HHHHH
          β1c   β2  L3         α1             β3    L4   α2a       L5
                                                   P-loop

Lmex      NEGVLQQSMREIWDRIAK-DPGNDYSCSVSYVQLYNEILTDLLDDSKGKVRIQMGLEGRG      175
rat       LMGIIPRIAHDIFDHIYSMDENLEFHIKVSYFEIYLDKIRDLLDVSKTNLAVHEDK---N      161
Nc        GRGVIPRIVEQIFTSILSSAANIEYTVRVSYMEIYMERIRDLLAPQNDNLPVHEEK---N      164
            *:: :  .:*:  * .   . ::   ***.::* : : ***  .: :: ::      .
             HHHHHHHHHHHHH       BBBBBBBBBBBB  BBBB
                  α2b        L6       β4     L7 β5a          L8

Lmex      DIVMVSDATGLPVEREVKDYKGTMAFFKAGLTRKEMASTSMNNTSSRSHTIFTLNVCKAQ      235
rat       RVPYVKGC----TERFVSSPEEVMDVIDEGKANRHVAVTNMNEHSSRSHSIFLINIKQEN      217
Nc        RGVYVKGL----LEIYVSSVQEVYEVMRRGGNARAVAATNMNQESSRSHSIFVITITQKN      220
              *..      *  *.. : .  .:  *   : :* *.**: *****:** :.: : :
                        BB   HHHHHHHHHHHHHHHHHHHHH    HHH BBBBBBBBBBBB
                        β5b         α3         L9   α3a       β6
                                                    switch-1

Lmex      RVGTVTVGAETEGPTIALEGRLVLCDLAGSERVSKTHAEGKTLDEATHINRSLLTLGKVV      295
rat       -----------VETEKKLSGKLYLVDLAGSEKVSKTGAEGAVLDEAKNINKSLSALGNVI      266
Nc        -----------VETGSAKSGQLFLVDLAGSEKVGKTGASGQTLEEAKKINKSLSALGMVI      269
                            .*:* * ******:*.** *.* .*:**.:**:** :** *:
                         BBBBBBBBBBB                         HHHHHHHHH
                    L10      β7                 L11              α4
                                    switch-2

Lmex      TALTDN-AQHAPFRESKLTRILQYSLLGNGNTSIIVNISPSDENTEESLSTLFFGQRASQ      354
rat       SALAEGTKTHVPYRDSKMTRILQDSLGGNCRTTIVICCSPSVFNEAETKSTLMFGQRAKT      326
Nc        NALTDGKSSHVPYRDSKLTRILQESLGGNSRTTLIINCSPSSYNDAETLSTLRFGMRAKS      329
          .**::.   *.*:*:**:***** ** ** .*::::  ***  *  *: *** ** **.
          HHHHH           HHHHH         BBBBBBBB      HHHHHHHHHHHHHHH
                   L12      α5       L13  β8      L14        α6

Lmex      IKQDAKRHEVLDYKALYLQLMADIDNKNDKTLEEALEEERGVYEDRISSLNEEMKLLNNE      414
rat       IKNTVSVNLELTAEEWKKKY-EK-E-----------KEKNKALKSVIQHLESELNRWRNG      373
Nc        IKNKAKVNAELSPAELKQML-AK-A-----------KTQITSFENYIVNLESEVQVWRGG      376
          **: .. :  *           .             : :    :. *  *:.*::  ..
           BBB   BBB  HHHHHHHHHHHHH
           β9    β10        α7
```

Figure S3: Alignment of LmxKIN29 (Lmex) with rat conventional kinesin (rat) and *Neurospora crassa* kinesin (Nc) with secondary structure elements highlighted according to the model and named according to elements in rat kinesin (Sack et al., 1997).

SACK, S., MULLER, J., MARX, A., THORMAHLEN, M., MANDELKOW, E. M., BRADY, S. T. & MANDELKOW, E. 1997. X-ray structure of motor and neck domains from rat brain kinesin. *Biochemistry,* 36**,** 16155-65.

Supplementary File 1

Evidence for LmxKin29 phosphorylation and presence of the protein in lesion-derived amastigotes by mass spectrometry:

>LmxM.29.0350.1 | *Leishmania mexicana* MHOM/GT/2001/U1103 | kinesin, putative | protein | length=610

```
MSRIQKSSKSAPKTISVYCRVRPPVPQEKGHNFNNIVYDDADNRTITVTRKSGSKSFEKRYFFNRVFR
PTVTQKDVYETFAKNAVDAAFDGQHGVLFVYGQTGSGKTFTISNDDPKNEGVLQQSMREIWDRIAKDP
GNDYSCSVSYVQLYNEILTDLLDDSKGKVRIQMGLEGRGDIVMVSDATGLPVEREVKDYKGTMAFFKA
GLTRKEMASTSMNNTSSRSHTIFTLNVCKAQRVGTVTVGAETEGPTIALEGRLVLCDLAGSERVSKTH
AEGKTLDEATHINRSLLTLGKVVTALTDNAQHAPFRESKLTRILQYSLLGNGNTSIIVNISPSDENTE
ESLSTLFFGQRASQIKQDAKRHEVLDYKALYLQLMADIDNKNDKTLEEALEEERGVYEDRISSLNEEM
KLLNNENAMLRNENKQLRQYVPADRLKLIDETPSSGVPGVNGGSISGGWAKANQELRELIQQRDEKMK
VISNERVRLALVVAEEKRKCFQLAQKLRSFAMRYKVEREQLTQRQEELTTELASLKGTDYLSAVGTFE
PTVSPASPGSPKFARDGEDFNDAESAQAQLRALWAERTELMLYQAKAANAIRMLVKEREAAQRKAA
```

Green: Unphosphorylated peptides identified in lesion-derived amastigotes.

Yellow: Phosphorylated peptide identified in promastigotes and axenic amastigotes.

Red: Phosphorylation sites.

The following shows data for peptides identified by tandem mass spectrometry. Data for peptides displayed in the fragmentation table and graph are those highlighted in blue, which had the highest Mascot score. The fragmentation table shows all expected b- and y-ions with those actually detected highlighted in red and blue. For their relative intensity see the graph below the table.

Promastigotes:

S554 phosphorylation (SIMAC)

Good?	...	Sequence	Prob	Masc...	Masc...	Masc...	NTT	Modifications	Observed	Actual Mass	Charge	Delta ...	Delta ...	S
☑	✓	(K)GTDYLSAVGTFEPTVSPASPGSPK(F	95%	77.8	29.1	0.0	2	Phospho (+80)	1,223.06	2,444.11	2	-0.0014	-0.56	

B	B Ions	B+2H	B-NH3	B-H2O	AA	Y Ions	Y+2H	Y-NH3	Y-H2O	Y
1	58.0				G	2,445.1	1,223.1	2,428.1	2,427.1	24
2	159.1			141.1	T	2,388.1	1,194.6	2,371.1	2,370.1	23
3	274.1			256.1	D	2,287.1	1,144.0	2,270.0	2,269.0	22
4	437.2			419.2	Y	2,172.0	1,086.5	2,155.0	2,154.0	21
5	550.3			532.2	L	2,009.0	1,005.0	1,991.9	1,991.0	20
6	637.3	319.1		619.3	S	1,895.9	948.4	1,878.9	1,877.9	19
7	708.3	354.7		690.3	A	1,808.8	904.9	1,791.8	1,790.8	18
8	807.4	404.2		789.4	V	1,737.8	869.4	1,720.8	1,719.8	17
9	864.4	432.7		846.4	G	1,638.7	819.9	1,621.7	1,620.7	16
10	965.5	483.2		947.4	T	1,581.7	791.4	1,564.7	1,563.7	15
11	1,112.5	556.8		1,094.5	F	1,480.7	740.8	1,463.6	1,462.7	14
12	1,241.6	621.3		1,223.6	E	1,333.6	667.3	1,316.6	1,315.6	13
13	1,338.6	669.8		1,320.6	P	1,204.6	602.8	1,187.5	1,186.6	12
14	1,439.7	720.3		1,421.7	T	1,107.5	554.3	1,090.5	1,089.5	11
15	1,538.7	769.9		1,520.7	V	1,006.5	503.7	989.4	988.5	10
16	1,625.8	813.4		1,607.8	S	907.4	454.2	890.4	889.4	9
17	1,722.8	861.9		1,704.8	P	820.4	410.7	803.3	802.3	8
18	1,793.9	897.4		1,775.8	A	723.3	362.2	706.3	705.3	7
19	1,880.9	940.9		1,862.9	S	652.3	326.6	635.2	634.3	6
20	1,977.9	989.5		1,959.9	P	565.2		548.2	547.2	5
21	2,035.0	1,018.0		2,017.0	G	468.2		451.2	450.2	4
22	2,202.0	1,101.5		2,184.0	S+80	411.2		394.1	393.2	3
23	2,299.0	1,150.0		2,281.0	P	244.2		227.1		2
24	2,445.1	1,223.1	2,428.1	2,427.1	K	147.1		130.1		1

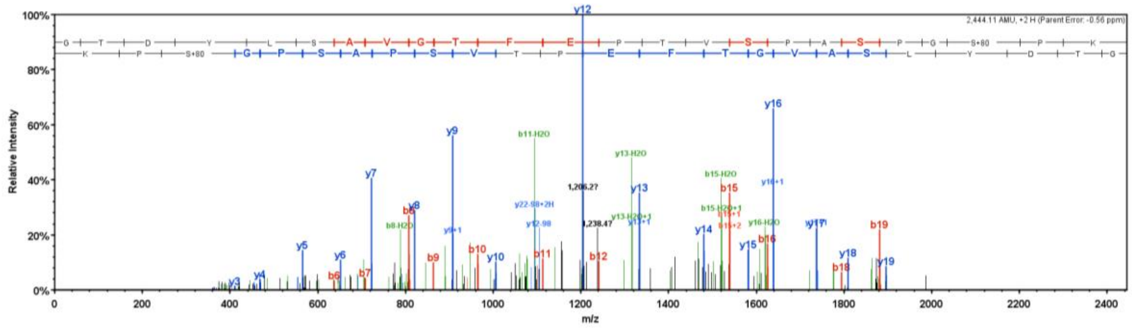

S551 phosphorylation in promastigotes:

Good?	…	Sequence	Prob	Masc…	Masc…	Masc…	NTT	Modifications	Observed	Actual Mass	Charge	Delta …	Delta …	S
☑	✓	(K)GTDYLSAVGTFEPTVSPASPGSPK(F	95%	39.3	31.8	0.0	2	Phospho (+80)	1,223.07	2,444.13	2	0.012	4.8	
☑	✓	(K)GTDYLSAVGTFEPTVSPASPGSPK(F	95%	51.8	31.9	0.0	2	Phospho (+80)	1,223.07	2,444.13	2	0.013	5.2	
☑	✓	(K)GTDYLSAVGTFEPTVSPASPGSPK(F	95%	56.1	31.9	0.0	2	Phospho (+80)	1,223.07	2,444.13	2	0.013	5.4	

B	B Ions	B+2H	B-NH3	B-H2O	AA	Y Ions	Y+2H	Y-NH3	Y-H2O	Y
1	58.0				G	2,445.1	1,223.1	2,428.1	2,427.1	24
2	159.1			141.1	T	2,388.1	1,194.6	2,371.1	2,370.1	23
3	274.1			256.1	D	2,287.1	1,144.0	2,270.0	2,269.0	22
4	437.2			419.2	Y	2,172.0	1,086.5	2,155.0	2,154.0	21
5	550.3			532.2	L	2,009.0	1,005.0	1,991.9	1,991.0	20
6	637.3	319.1		619.3	S	1,895.9	948.4	1,878.9	1,877.9	19
7	708.3	354.7		690.3	A	1,808.8	904.9	1,791.8	1,790.8	18
8	807.4	404.2		789.4	V	1,737.8	869.4	1,720.8	1,719.8	17
9	864.4	432.7		846.4	G	1,638.7	819.9	1,621.7	1,620.7	16
10	965.5	483.2		947.4	T	1,581.7	791.4	1,564.7	1,563.7	15
11	1,112.5	556.8		1,094.5	F	1,480.7	740.8	1,463.6	1,462.7	14
12	1,241.6	621.3		1,223.6	E	1,333.6	667.3	1,316.6	1,315.6	13
13	1,338.6	669.8		1,320.6	P	1,204.6	602.8	1,187.5	1,186.6	12
14	1,439.7	720.3		1,421.7	T	1,107.5	554.3	1,090.5	1,089.5	11
15	1,538.7	769.9		1,520.7	V	1,006.5	503.7	989.4	988.5	10
16	1,625.8	813.4		1,607.8	S	907.4	454.2	890.4	889.4	9
17	1,722.8	861.9		1,704.8	P	820.4	410.7	803.3	802.3	8
18	1,793.9	897.4		1,775.8	A	723.3	362.2	706.3	705.3	7
19	1,960.9	980.9		1,942.8	S+80	652.3	326.6	635.2	634.3	6
20	2,057.9	1,029.5		2,039.9	P	485.3		468.2	467.3	5
21	2,114.9	1,058.0		2,096.9	G	388.2		371.2	370.2	4
22	2,202.0	1,101.5		2,184.0	S	331.2		314.2	313.2	3
23	2,299.0	1,150.0		2,281.0	P	244.2		227.1		2
24	2,445.1	1,223.1	2,428.1	2,427.1	K	147.1		130.1		1

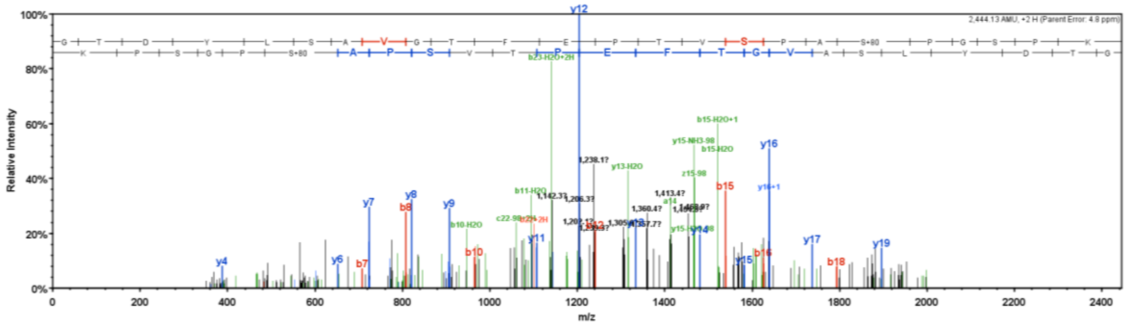

S548 phosphorylation in axenic amastigotes:

Good?	...	Sequence	Prob	Masc...	Masc...	Masc...	NTT	Modifications	Observed	Actual Mass	Charge	Delta ...	Delta ...	S
☑	✓	(K)GTDYLSAVGTFEPTVSPASPGSPK(F	95%	27.1	28.9	11.8	2	Phospho (+80)	1,223.06	2,444.11	2	-0.0013	-0.52	
☑	✓	(K)GTDYLSAVGTFEPTVSPASPGSPK(F	95%	48.3	29.0	7.8	2	Phospho (+80)	1,223.06	2,444.11	2	-0.0057	-2.4	
☑	✓	(K)GTDYLSAVGTFEPTVSPASPGSPK(F	95%	52.0	28.8	1.5	2	Phospho (+80)	1,223.07	2,444.12	2	0.0040	1.6	
☑	✓	(K)GTDYLSAVGTFEPTVSPASPGSPK(F	95%	54.0	29.4	24.2	2	Phospho (+80), P...	1,263.05	2,524.08	2	-0.0034	-1.3	
☑	✓	(K)GTDYLSAVGTFEPTVSPASPGSPK(F	95%	54.0	29.0	11.1	2	Phospho (+80)	1,223.06	2,444.11	2	-0.0060	-2.5	
☑	✓	(K)GTDYLSAVGTFEPTVSPASPGSPK(F	95%	58.3	29.5	29.7	2	Phospho (+80), P...	1,263.05	2,524.08	2	0.00055	0.22	
☑	✓	(K)GTDYLSAVGTFEPTVSPASPGSPK(F	95%	59.7	28.9	8.7	2	Phospho (+80)	1,223.06	2,444.11	2	-0.0014	-0.56	
☑	✓	(K)GTDYLSAVGTFEPTVSPASPGSPK(F	95%	68.5	29.1	20.7	2	Phospho (+80), P...	1,263.05	2,524.09	2	0.0049	1.9	
☑	✓	(K)GTDYLSAVGTFEPTVSPASPGSPK(F	95%	72.1	29.1	12.5	2	Phospho (+80), P...	1,263.05	2,524.09	2	0.0068	2.7	
☑	✓	(K)GTDYLSAVGTFEPTVSPASPGSPK(F	95%	79.0	29.1	38.2	2	Phospho (+80), P...	1,263.05	2,524.08	2	-0.00020	-0.079	
☑	✓	(K)GTDYLSAVGTFEPTVSPASPGSPK(F	95%	83.8	28.8	20.6	2	Phospho (+80)	1,223.07	2,444.12	2	0.0034	1.4	
☑	✓	(K)GTDYLSAVGTFEPTVSPASPGSPK(F	95%	93.3	28.8	22.6	2	Phospho (+80)	1,223.07	2,444.12	2	0.0047	1.9	
☑	✓	(K)GTDYLSAVGTFEPTVSPASPGSPK(F	95%	94.8	29.4	48.3	2	Phospho (+80), P...	1,263.05	2,524.08	2	0.0019	0.74	
☑	✓	(K)GTDYLSAVGTFEPTVSPASPGSPK(F	95%	97.9	29.1	51.4	2	Phospho (+80), P...	1,263.05	2,524.09	2	0.0057	2.2	
☑	✓	(K)GTDYLSAVGTFEPTVSPASPGSPK(F	95%	111.2	29.0	66.2	2	Phospho (+80), P...	1,263.05	2,524.09	2	0.0063	2.5	

B	B Ions	B+2H	B-NH3	B-H2O	AA	Y Ions	Y+2H	Y-NH3	Y-H2O	Y
1	58.0				G	2,445.1	1,223.1	2,428.1	2,427.1	24
2	159.1			141.1	T	2,388.1	1,194.6	2,371.1	2,370.1	23
3	274.1			256.1	D	2,287.1	1,144.0	2,270.0	2,269.0	22
4	437.2			419.2	Y	2,172.0	1,086.5	2,155.0	2,154.0	21
5	550.3			532.2	L	2,009.0	1,005.0	1,991.9	1,991.0	20
6	637.3	319.1		619.3	S	1,895.9	948.4	1,878.9	1,877.9	19
7	708.3	354.7		690.3	A	1,808.8	904.9	1,791.8	1,790.8	18
8	807.4	404.2		789.4	V	1,737.8	869.4	1,720.8	1,719.8	17
9	864.4	432.7		846.4	G	1,638.7	819.9	1,621.7	1,620.7	16
10	965.5	483.2		947.4	T	1,581.7	791.4	1,564.7	1,563.7	15
11	1,112.5	556.8		1,094.5	F	1,480.7	740.8	1,463.6	1,462.7	14
12	1,241.6	621.3		1,223.6	E	1,333.6	667.3	1,316.6	1,315.6	13
13	1,338.6	669.8		1,320.6	P	1,204.6	602.8	1,187.5	1,186.6	12
14	1,439.7	720.3		1,421.7	T	1,107.5	554.3	1,090.5	1,089.5	11
15	1,538.7	769.9		1,520.7	V	1,006.5	503.7	989.4	988.4	10
16	1,705.7	853.4		1,687.7	S+80	907.4	454.2	890.4	889.4	9
17	1,802.8	901.9		1,784.8	P	740.4	370.7	723.4	722.4	8
18	1,873.8	937.4		1,855.8	A	643.3	322.2	626.3	625.3	7
19	1,960.9	980.9		1,942.8	S	572.3	286.7	555.3	554.3	6
20	2,057.9	1,029.5		2,039.9	P	485.3		468.2	467.3	5
21	2,114.9	1,058.0		2,096.9	G	388.2		371.2	370.2	4
22	2,202.0	1,101.5		2,184.0	S	331.2		314.2	313.2	3
23	2,299.0	1,150.0		2,281.0	P	244.2		227.1		2
24	2,445.1	1,223.1	2,428.1	2,427.1	K	147.1		130.1		1

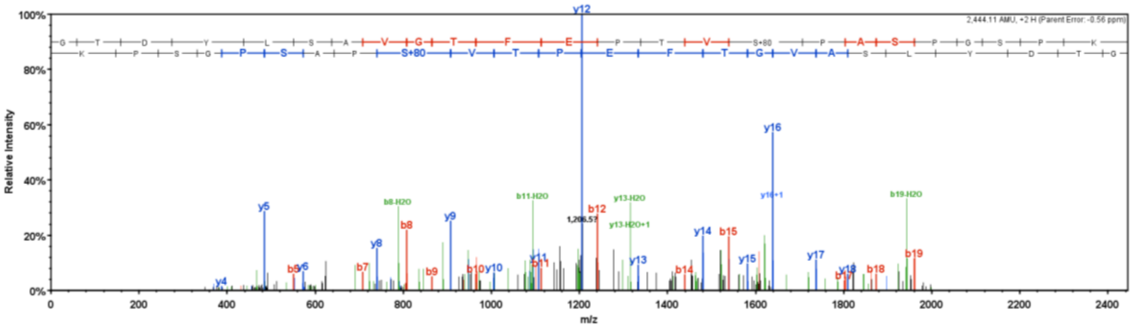

S551 phosphorylation in axenic amastigotes:

Good?	...	Sequence	Prob	Masc...	Masc...	Masc...	NTT	Modifications	Observed	Actual Mass	Charge	Delta ...	Delta ...
☑	✓	(K)GTDYLSAVGTFEPTVSPASPGSPK(F	95%	27.1	28.9	11.8	2	Phospho (+80)	1,223.06	2,444.11	2	-0.0013	-0.52
☑	✓	(K)GTDYLSAVGTFEPTVSPASPGSPK(F	95%	48.3	29.0	7.8	2	Phospho (+80)	1,223.06	2,444.11	2	-0.0057	-2.4
☑	✓	(K)GTDYLSAVGTFEPTVSPASPGSPK(F	95%	52.0	28.8	1.5	2	Phospho (+80)	1,223.07	2,444.12	2	0.0040	1.6
☑	✓	(K)GTDYLSAVGTFEPTVSPASPGSPK(F	95%	54.0	29.4	24.2	2	Phospho (+80), P...	1,263.05	2,524.08	2	-0.0034	-1.3
☑	✓	(K)GTDYLSAVGTFEPTVSPASPGSPK(F	95%	54.0	29.0	11.1	2	Phospho (+80)	1,223.06	2,444.11	2	-0.0060	-2.5
☑	✓	(K)GTDYLSAVGTFEPTVSPASPGSPK(F	95%	58.3	29.5	29.7	2	Phospho (+80), P...	1,263.05	2,524.08	2	0.00055	0.22
☑	✓	(K)GTDYLSAVGTFEPTVSPASPGSPK(F	95%	59.7	28.9	8.7	2	Phospho (+80)	1,223.06	2,444.11	2	-0.0014	-0.56
☑	✓	(K)GTDYLSAVGTFEPTVSPASPGSPK(F	95%	68.5	29.1	20.7	2	Phospho (+80), P...	1,263.05	2,524.09	2	0.0049	1.9
☑	✓	(K)GTDYLSAVGTFEPTVSPASPGSPK(F	95%	72.1	29.1	12.5	2	Phospho (+80), P...	1,263.05	2,524.09	2	0.0068	2.7
☑	✓	(K)GTDYLSAVGTFEPTVSPASPGSPK(F	95%	79.0	29.1	38.2	2	Phospho (+80), P...	1,263.05	2,524.08	2	-0.00020	-0.079
☑	✓	(K)GTDYLSAVGTFEPTVSPASPGSPK(F	95%	83.8	28.8	20.6	2	Phospho (+80)	1,223.07	2,444.12	2	0.0034	1.4
☑	✓	(K)GTDYLSAVGTFEPTVSPASPGSPK(F	95%	93.3	28.8	22.6	2	Phospho (+80)	1,223.07	2,444.12	2	0.0047	1.9
☑	✓	(K)GTDYLSAVGTFEPTVSPASPGSPK(F	95%	94.8	29.4	48.3	2	Phospho (+80), P...	1,263.05	2,524.08	2	0.0019	0.74
☑	✓	(K)GTDYLSAVGTFEPTVSPASPGSPK(F	95%	97.9	29.1	51.4	2	Phospho (+80), P...	1,263.05	2,524.09	2	0.0057	2.2
☑	✓	(K)GTDYLSAVGTFEPTVSPASPGSPK(F	95%	111.2	29.0	66.2	2	Phospho (+80), P...	1,263.05	2,524.09	2	0.0063	2.5

B	B Ions	B+2H	B-NH3	B-H2O	AA	Y Ions	Y+2H	Y-NH3	Y-H2O	Y
1	58.0				G	2,445.1	1,223.1	2,428.1	2,427.1	24
2	159.1			141.1	T	2,388.1	1,194.6	2,371.1	2,370.1	23
3	274.1			256.1	D	2,287.1	1,144.0	2,270.0	2,269.0	22
4	437.2			419.2	Y	2,172.0	1,086.5	2,155.0	2,154.0	21
5	550.3			532.2	L	2,009.0	1,005.0	1,991.9	1,991.0	20
6	637.3	319.1		619.3	S	1,895.9	948.4	1,878.9	1,877.9	19
7	708.3	354.7		690.3	A	1,808.8	904.9	1,791.8	1,790.8	18
8	807.4	404.2		789.4	V	1,737.8	869.4	1,720.8	1,719.8	17
9	864.4	432.7		846.4	G	1,638.7	819.9	1,621.7	1,620.7	16
10	965.5	483.2		947.4	T	1,581.7	791.4	1,564.7	1,563.7	15
11	1,112.5	556.8		1,094.5	F	1,480.7	740.8	1,463.6	1,462.7	14
12	1,241.6	621.3		1,223.6	E	1,333.6	667.3	1,316.6	1,315.6	13
13	1,338.6	669.8		1,320.6	P	1,204.6	602.8	1,187.5	1,186.6	12
14	1,439.7	720.3		1,421.7	T	1,107.5	554.3	1,090.5	1,089.5	11
15	1,538.7	769.9		1,520.7	V	1,006.5	503.7	989.4	988.5	10
16	1,625.8	813.4		1,607.8	S	907.4	454.2	890.4	889.4	9
17	1,722.8	861.9		1,704.8	P	820.4	410.7	803.3	802.3	8
18	1,793.9	897.4		1,775.8	A	723.3	362.2	706.3	705.3	7
19	1,960.9	980.9		1,942.8	S+80	652.3	326.6	635.2	634.3	6
20	2,057.9	1,029.5		2,039.9	P	485.3		468.2	467.3	5
21	2,114.9	1,058.0		2,096.9	G	388.2		371.2	370.2	4
22	2,202.0	1,101.5		2,184.0	S	331.2		314.2	313.2	3
23	2,299.0	1,150.0		2,281.0	P	244.2		227.1		2
24	2,445.1	1,223.1	2,428.1	2,427.1	K	147.1		130.1		1

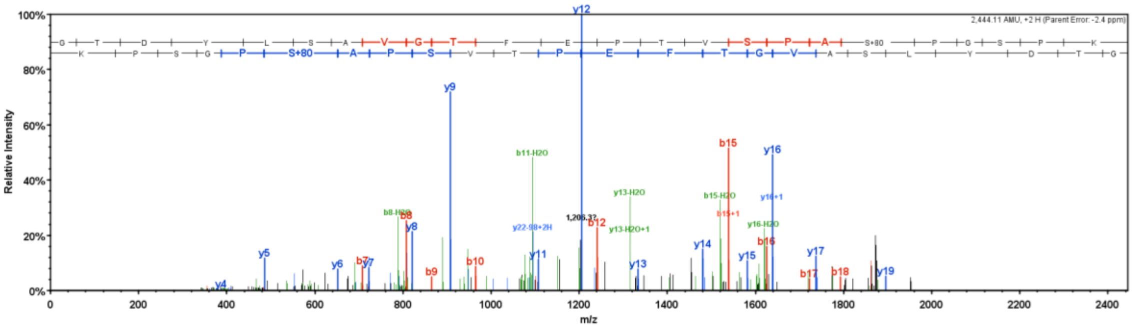

S554 phosphorylation in axenic amastigotes:

Good?	...	Sequence	Prob	Masc...	Masc...	Masc...	NTT	Modifications	Observed	Actual Mass	Charge	Delta ...	Delta ...	S
☑	✓	(K)GTDYLSAVGTFEPTVSPASPGSPK(F	95%	27.1	28.9	11.8	2	Phospho (+80)	1,223.06	2,444.11	2	-0.0013	-0.52	
☑	✓	(K)GTDYLSAVGTFEPTVSPASPGSPK(F	95%	48.3	29.0	7.8	2	Phospho (+80)	1,223.06	2,444.11	2	-0.0057	-2.4	
☑	✓	(K)GTDYLSAVGTFEPTVSPASPGSPK(F	95%	52.0	28.8	1.5	2	Phospho (+80)	1,223.07	2,444.12	2	0.0040	1.6	
☑	✓	(K)GTDYLSAVGTFEPTVSPASPGSPK(F	95%	54.0	29.4	24.2	2	Phospho (+80), P...	1,263.05	2,524.08	2	-0.0034	-1.3	
☑	✓	(K)GTDYLSAVGTFEPTVSPASPGSPK(F	95%	54.0	29.0	11.1	2	Phospho (+80)	1,223.06	2,444.11	2	-0.0060	-2.5	
☑	✓	(K)GTDYLSAVGTFEPTVSPASPGSPK(F	95%	58.3	29.5	29.7	2	Phospho (+80), P...	1,263.05	2,524.08	2	0.00055	0.22	
☑	✓	(K)GTDYLSAVGTFEPTVSPASPGSPK(F	95%	59.7	28.9	8.7	2	Phospho (+80)	1,223.06	2,444.11	2	-0.0014	-0.56	
☑	✓	(K)GTDYLSAVGTFEPTVSPASPGSPK(F	95%	68.5	29.1	20.7	2	Phospho (+80), P...	1,263.05	2,524.09	2	0.0049	1.9	
☑	✓	(K)GTDYLSAVGTFEPTVSPASPGSPK(F	95%	72.1	29.1	12.5	2	Phospho (+80), P...	1,263.05	2,524.09	2	0.0068	2.7	
☑	✓	(K)GTDYLSAVGTFEPTVSPASPGSPK(F	95%	79.0	29.1	38.2	2	Phospho (+80), P...	1,263.05	2,524.08	2	-0.00020	-0.079	
☑	✓	(K)GTDYLSAVGTFEPTVSPASPGSPK(F	95%	83.8	28.8	20.6	2	Phospho (+80)	1,223.07	2,444.12	2	0.0034	1.4	
☑	✓	(K)GTDYLSAVGTFEPTVSPASPGSPK(F	95%	93.3	28.8	22.6	2	Phospho (+80)	1,223.07	2,444.12	2	0.0047	1.9	
☑	✓	(K)GTDYLSAVGTFEPTVSPASPGSPK(F	95%	94.8	29.4	48.3	2	Phospho (+80), P...	1,263.05	2,524.08	2	0.0019	0.74	
☑	✓	(K)GTDYLSAVGTFEPTVSPASPGSPK(F	95%	97.9	29.1	51.4	2	Phospho (+80), P...	1,263.05	2,524.09	2	0.0057	2.2	
☑	✓	(K)GTDYLSAVGTFEPTVSPASPGSPK(F	95%	111.2	29.0	66.2	2	Phospho (+80), P...	1,263.05	2,524.09	2	0.0063	2.5	

B	B Ions	B+2H	B-NH3	B-H2O	AA	Y Ions	Y+2H	Y-NH3	Y-H2O	Y
1	58.0				G	2,445.1	1,223.1	2,428.1	2,427.1	24
2	159.1			141.1	T	2,388.1	1,194.6	2,371.1	2,370.1	23
3	274.1			256.1	D	2,287.1	1,144.0	2,270.0	2,269.0	22
4	437.2			419.2	Y	2,172.0	1,086.5	2,155.0	2,154.0	21
5	550.3			532.2	L	2,009.0	1,005.0	1,991.9	1,991.0	20
6	637.3	319.1		619.3	S	1,895.9	948.4	1,878.9	1,877.9	19
7	708.3	354.7		690.3	A	1,808.8	904.9	1,791.8	1,790.8	18
8	807.4	404.2		789.4	V	1,737.8	869.4	1,720.8	1,719.8	17
9	864.4	432.7		846.4	G	1,638.7	819.9	1,621.7	1,620.7	16
10	965.5	483.2		947.4	T	1,581.7	791.4	1,564.7	1,563.7	15
11	1,112.5	556.8		1,094.5	F	1,480.7	740.8	1,463.6	1,462.7	14
12	1,241.6	621.3		1,223.6	E	1,333.6	667.3	1,316.6	1,315.6	13
13	1,338.6	669.8		1,320.6	P	1,204.6	602.8	1,187.5	1,186.6	12
14	1,439.7	720.3		1,421.7	T	1,107.5	554.3	1,090.5	1,089.5	11
15	1,538.7	769.9		1,520.7	V	1,006.5	503.7	989.4	988.5	10
16	1,625.8	813.4		1,607.8	S	907.4	454.2	890.4	889.4	9
17	1,722.8	861.9		1,704.8	P	820.4	410.7	803.3	802.3	8
18	1,793.9	897.4		1,775.8	A	723.3	362.2	706.3	705.3	7
19	1,880.9	940.9		1,862.9	S	652.3	326.6	635.2	634.3	6
20	1,977.9	989.5		1,959.9	P	565.2		548.2	547.2	5
21	2,035.0	1,018.0		2,017.0	G	468.2		451.2	450.2	4
22	2,202.0	1,101.5		2,184.0	S+80	411.2		394.1	393.2	3
23	2,299.0	1,150.0		2,281.0	P	244.2		227.1		2
24	2,445.1	1,223.1	2,428.1	2,427.1	K	147.1		130.1		1

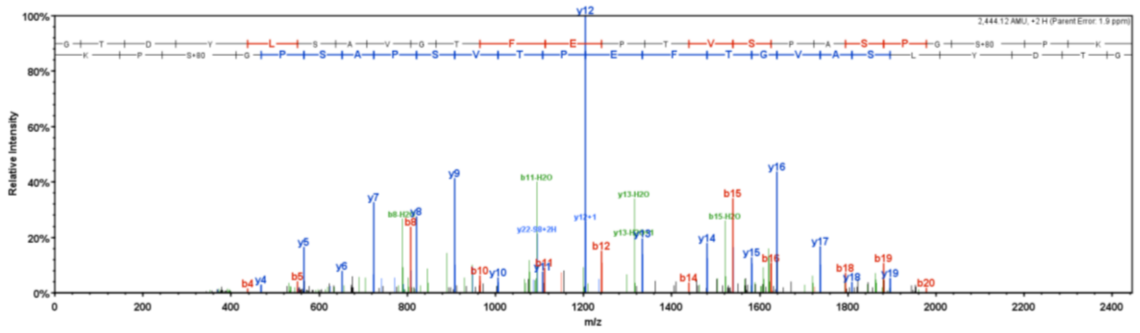

S548/S551 phosphorylation in axenic amastigotes:

Good?	...	Sequence	Prob	Masc...	Masc...	Masc...	NTT	Modifications	Observed	Actual Mass	Charge	Delta ...	Delta ...	S
☑	✓	(K)GTDYLSAVGTFEPTVSPASPGSPK(F	95%	27.1	28.9	11.8	2	Phospho (+80)	1,223.06	2,444.11	2	-0.0013	-0.52	
☑	✓	(K)GTDYLSAVGTFEPTVSPASPGSPK(F	95%	48.3	29.0	7.8	2	Phospho (+80)	1,223.06	2,444.11	2	-0.0057	-2.4	
☑	✓	(K)GTDYLSAVGTFEPTVSPASPGSPK(F	95%	52.0	28.8	1.5	2	Phospho (+80)	1,223.07	2,444.12	2	0.0040	1.6	
☑	✓	(K)GTDYLSAVGTFEPTVSPASPGSPK(F	95%	54.0	29.4	24.2	2	Phospho (+80), P...	1,263.05	2,524.08	2	-0.0034	-1.3	
☑	✓	(K)GTDYLSAVGTFEPTVSPASPGSPK(F	95%	54.0	29.0	11.1	2	Phospho (+80)	1,223.06	2,444.11	2	-0.0060	-2.5	
☑	✓	(K)GTDYLSAVGTFEPTVSPASPGSPK(F	95%	58.3	29.5	29.7	2	Phospho (+80), P...	1,263.05	2,524.08	2	0.00055	0.22	
☑	✓	(K)GTDYLSAVGTFEPTVSPASPGSPK(F	95%	59.7	28.9	8.7	2	Phospho (+80)	1,223.06	2,444.11	2	-0.0014	-0.56	
☑	✓	(K)GTDYLSAVGTFEPTVSPASPGSPK(F	95%	68.5	29.1	20.7	2	Phospho (+80), P...	1,263.05	2,524.09	2	0.0049	1.9	
☑	✓	(K)GTDYLSAVGTFEPTVSPASPGSPK(F	95%	72.1	29.1	12.5	2	Phospho (+80), P...	1,263.05	2,524.09	2	0.0068	2.7	
☑	✓	(K)GTDYLSAVGTFEPTVSPASPGSPK(F	95%	79.0	29.1	38.2	2	Phospho (+80), P...	1,263.05	2,524.08	2	-0.00020	-0.079	
☑	✓	(K)GTDYLSAVGTFEPTVSPASPGSPK(F	95%	83.8	28.8	20.6	2	Phospho (+80)	1,223.07	2,444.12	2	0.0034	1.4	
☑	✓	(K)GTDYLSAVGTFEPTVSPASPGSPK(F	95%	93.3	28.8	22.6	2	Phospho (+80)	1,223.07	2,444.12	2	0.0047	1.9	
☑	✓	(K)GTDYLSAVGTFEPTVSPASPGSPK(F	95%	94.8	29.4	48.3	2	Phospho (+80), P...	1,263.05	2,524.08	2	0.0019	0.74	
☑	✓	(K)GTDYLSAVGTFEPTVSPASPGSPK(F	95%	97.9	29.1	51.4	2	Phospho (+80), P...	1,263.05	2,524.09	2	0.0057	2.2	
☑	✓	(K)GTDYLSAVGTFEPTVSPASPGSPK(F	95%	111.2	29.0	66.2	2	Phospho (+80), P...	1,263.05	2,524.09	2	0.0063	2.5	

B	B Ions	B+2H	B-NH3	B-H2O	AA	Y Ions	Y+2H	Y-NH3	Y-H2O	Y
1	58.0				G	2,525.1	1,263.0	2,508.1	2,507.1	24
2	159.1			141.1	T	2,468.1	1,234.5	2,451.0	2,450.1	23
3	274.1			256.1	D	2,367.0	1,184.0	2,350.0	2,349.0	22
4	437.2			419.2	Y	2,252.0	1,126.5	2,235.0	2,234.0	21
5	550.3			532.2	L	2,088.9	1,045.0	2,071.9	2,070.9	20
6	637.3	319.1		619.3	S	1,975.8	988.4	1,958.8	1,957.8	19
7	708.3	354.7		690.3	A	1,888.8	944.9	1,871.8	1,870.8	18
8	807.4	404.2		789.4	V	1,817.8	909.4	1,800.7	1,799.8	17
9	864.4	432.7		846.4	G	1,718.7	859.9	1,701.7	1,700.7	16
10	965.5	483.2		947.4	T	1,661.7	831.3	1,644.7	1,643.7	15
11	1,112.5	556.8		1,094.5	F	1,560.6	780.8	1,543.6	1,542.6	14
12	1,241.6	621.3		1,223.6	E	1,413.6	707.3	1,396.5	1,395.6	13
13	1,338.6	669.8		1,320.6	P	1,284.5	642.8	1,267.5	1,266.5	12
14	1,439.7	720.3		1,421.7	T	1,187.5	594.2	1,170.4	1,169.5	11
15	1,538.7	769.9		1,520.7	V	1,086.4	543.7	1,069.4	1,068.4	10
16	1,705.7	853.4		1,687.7	S+80	987.4	494.2	970.3	969.3	9
17	1,802.8	901.9		1,784.8	P	820.4	410.7	803.3	802.3	8
18	1,873.8	937.4		1,855.8	A	723.3	362.2	706.3	705.3	7
19	2,040.8	1,020.9		2,022.8	S+80	652.3	326.6	635.2	634.3	6
20	2,137.9	1,069.4		2,119.9	P	485.3		468.2	467.3	5
21	2,194.9	1,098.0		2,176.9	G	388.2		371.2	370.2	4
22	2,281.9	1,141.5		2,263.9	S	331.2		314.2	313.2	3
23	2,379.0	1,190.0		2,361.0	P	244.2		227.1		2
24	2,525.1	1,263.0	2,508.1	2,507.1	K	147.1		130.1		1

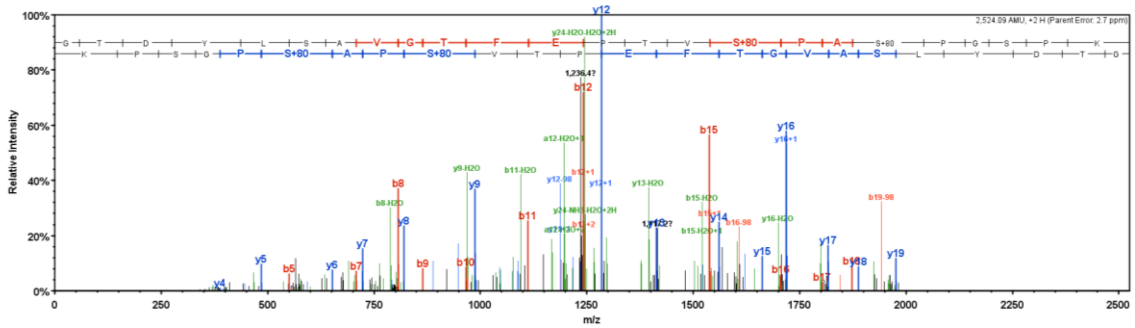

S551/S554 phosphorylation in axenic amastigotes:

Good?	...	Sequence	Prob	Masc...	Masc...	Masc...	NTT	Modifications	Observed	Actual Mass	Charge	Delta ...	Delta ...	S
☑	✓	(K)GTDYLSAVGTFEPTVSPASPGSPK(F	95%	27.1	28.9	11.8	2	Phospho (+80)	1,223.06	2,444.11	2	-0.0013	-0.52	
☑	✓	(K)GTDYLSAVGTFEPTVSPASPGSPK(F	95%	48.3	29.0	7.8	2	Phospho (+80)	1,223.06	2,444.11	2	-0.0057	-2.4	
☑	✓	(K)GTDYLSAVGTFEPTVSPASPGSPK(F	95%	52.0	28.8	1.5	2	Phospho (+80)	1,223.07	2,444.12	2	0.0040	1.6	
☑	✓	(K)GTDYLSAVGTFEPTVSPASPGSPK(F	95%	54.0	29.4	24.2	2	Phospho (+80), P...	1,263.05	2,524.08	2	-0.0034	-1.3	
☑	✓	(K)GTDYLSAVGTFEPTVSPASPGSPK(F	95%	54.0	29.0	11.1	2	Phospho (+80)	1,223.06	2,444.11	2	-0.0060	-2.5	
☑	✓	(K)GTDYLSAVGTFEPTVSPASPGSPK(F	95%	58.3	29.5	29.7	2	Phospho (+80), P...	1,263.05	2,524.08	2	0.00055	0.22	
☑	✓	(K)GTDYLSAVGTFEPTVSPASPGSPK(F	95%	59.7	28.9	8.7	2	Phospho (+80)	1,223.06	2,444.11	2	-0.0014	-0.56	
☑	✓	(K)GTDYLSAVGTFEPTVSPASPGSPK(F	95%	68.5	29.1	20.7	2	Phospho (+80), P...	1,263.05	2,524.09	2	0.0049	1.9	
☑	✓	(K)GTDYLSAVGTFEPTVSPASPGSPK(F	95%	72.1	29.1	12.5	2	Phospho (+80), P...	1,263.05	2,524.09	2	0.0068	2.7	
☑	✓	(K)GTDYLSAVGTFEPTVSPASPGSPK(F	95%	79.0	29.1	38.2	2	Phospho (+80), P...	1,263.05	2,524.08	2	-0.00020	-0.079	
☑	✓	(K)GTDYLSAVGTFEPTVSPASPGSPK(F	95%	83.8	28.8	20.6	2	Phospho (+80)	1,223.07	2,444.12	2	0.0034	1.4	
☑	✓	(K)GTDYLSAVGTFEPTVSPASPGSPK(F	95%	93.3	28.8	22.6	2	Phospho (+80)	1,223.07	2,444.12	2	0.0047	1.9	
☑	✓	(K)GTDYLSAVGTFEPTVSPASPGSPK(F	95%	94.8	29.4	48.3	2	Phospho (+80), P...	1,263.05	2,524.08	2	0.0019	0.74	
☑	✓	(K)GTDYLSAVGTFEPTVSPASPGSPK(F	95%	97.9	29.1	51.4	2	Phospho (+80), P...	1,263.05	2,524.09	2	0.0057	2.2	
☑	✓	(K)GTDYLSAVGTFEPTVSPASPGSPK(F	95%	111.2	29.0	66.2	2	Phospho (+80), P...	1,263.05	2,524.09	2	0.0063	2.5	

B	B Ions	B+2H	B-NH3	B-H2O	AA	Y Ions	Y+2H	Y-NH3	Y-H2O	Y
1	58.0				G	2,525.1	1,263.0	2,508.1	2,507.1	24
2	159.1			141.1	T	2,468.1	1,234.5	2,451.0	2,450.1	23
3	274.1			256.1	D	2,367.0	1,184.0	2,350.0	2,349.0	22
4	437.2			419.2	Y	2,252.0	1,126.5	2,235.0	2,234.0	21
5	550.3			532.2	L	2,088.9	1,045.0	2,071.9	2,070.9	20
6	637.3	319.1		619.3	S	1,975.8	988.4	1,958.8	1,957.8	19
7	708.3	354.7		690.3	A	1,888.8	944.9	1,871.8	1,870.8	18
8	807.4	404.2		789.4	V	1,817.8	909.4	1,800.7	1,799.8	17
9	864.4	432.7		846.4	G	1,718.7	859.9	1,701.7	1,700.7	16
10	965.5	483.2		947.4	T	1,661.7	831.3	1,644.7	1,643.7	15
11	1,112.5	556.8		1,094.5	F	1,560.6	780.8	1,543.6	1,542.6	14
12	1,241.6	621.3		1,223.6	E	1,413.6	707.3	1,396.5	1,395.6	13
13	1,338.6	669.8		1,320.6	P	1,284.5	642.8	1,267.5	1,266.5	12
14	1,439.7	720.3		1,421.7	T	1,187.5	594.2	1,170.4	1,169.5	11
15	1,538.7	769.9		1,520.7	V	1,086.4	543.7	1,069.4	1,068.4	10
16	1,625.8	813.4		1,607.8	S	987.4	494.2	970.3	969.3	9
17	1,722.8	861.9		1,704.8	P	900.3	450.7	883.3	882.3	8
18	1,793.9	897.4		1,775.8	A	803.3	402.1	786.2	785.3	7
19	1,960.9	980.9		1,942.8	S+80	732.2	366.6	715.2	714.2	6
20	2,057.9	1,029.5		2,039.9	P	565.2		548.2	547.2	5
21	2,114.9	1,058.0		2,096.9	G	468.2		451.2	450.2	4
22	2,281.9	1,141.5		2,263.9	S+80	411.2		394.1	393.2	3
23	2,379.0	1,190.0		2,361.0	P	244.2		227.1		2
24	2,525.1	1,263.0	2,508.1	2,507.1	K	147.1		130.1		1

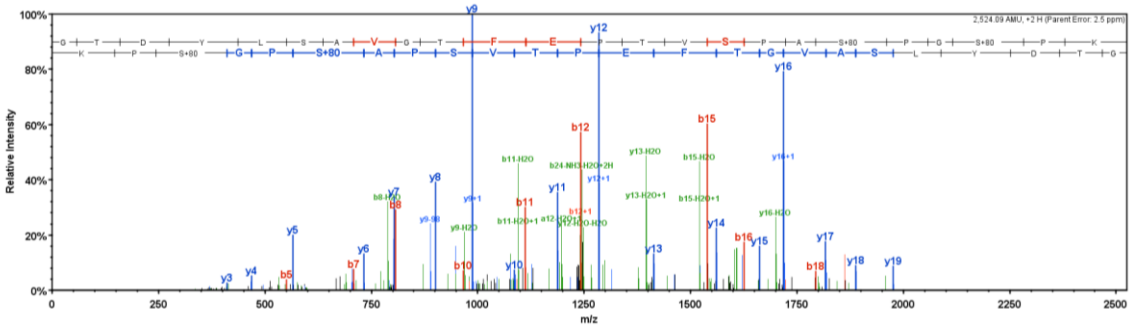

Lesion-derived amastigotes LmxKin29 peptides:

Good?	...	Sequence	Prob	Masc...	Masc...	Masc...	NTT	Modifications	Observed	Actual Mass	Charge	Delta ...	Delta ...	S
☑	✓	(K)EMASTSMNNTSSR(S)	95%	60.7	32.9	45.5	2		708.30	1,414.59	2	0.0081	5.8	
☑	✓	(R)GDIVMVSDATGLPVER(E)	95%	35.5	28.6	26.0	2		829.93	1,657.85	2	0.013	7.6	
☑	✓	(R)GDIVMVSDATGLPVER(E)	95%	49.6	30.9	42.3	2		829.93	1,657.84	2	0.0057	3.4	
☑	✓	(R)GDIVMVSDATGLPVER(E)	95%	51.4	30.3	40.6	2		829.93	1,657.85	2	0.013	7.9	
☑	✓	(R)GDIVMVSDATGLPVER(E)	95%	56.0	30.3	42.3	2		829.93	1,657.85	2	0.013	7.9	
☑	✓	(R)GDIVMVSDATGLPVER(E)	95%	58.8	30.6	49.2	2		829.93	1,657.84	2	0.0090	5.4	
☑	✓	(K)TLDEATHINR(S)	95%	40.5	30.5	23.8	2		585.30	1,168.59	2	0.0047	4.0	
☑	✓	(K)TLEEALEEER(G)	95%	45.0	33.1	33.4	2		609.80	1,217.59	2	0.0078	6.4	

B	B Ions	B+2H	B-NH3	B-H2O	AA	Y Ions	Y+2H	Y-NH3	Y-H2O	Y
1	58.0				G	1,658.8	829.9	1,641.8	1,640.8	16
2	173.1			155.0	D	1,601.8	801.4	1,584.8	1,583.8	15
3	286.1			268.1	I	1,486.8	743.9	1,469.8	1,468.8	14
4	385.2			367.2	V	1,373.7	687.4	1,356.7	1,355.7	13
5	516.2			498.2	M	1,274.6	637.8	1,257.6	1,256.6	12
6	615.3	308.2		597.3	V	1,143.6	572.3	1,126.6	1,125.6	11
7	702.3	351.7		684.3	S	1,044.5	522.8	1,027.5	1,026.5	10
8	817.4	409.2		799.4	D	957.5	479.3	940.5	939.5	9
9	888.4	444.7		870.4	A	842.5	421.7	825.4	824.5	8
10	989.5	495.2		971.5	T	771.4	386.2	754.4	753.4	7
11	1,046.5	523.7		1,028.5	G	670.4	335.7	653.4	652.4	6
12	1,159.6	580.3		1,141.6	L	613.4		596.3	595.4	5
13	1,256.6	628.8		1,238.6	P	500.3		483.3	482.3	4
14	1,355.7	678.3		1,337.7	V	403.2		386.2	385.2	3
15	1,484.7	742.9		1,466.7	E	304.2		287.1	286.2	2
16	1,658.8	829.9	1,641.8	1,640.8	R	175.1		158.1		1

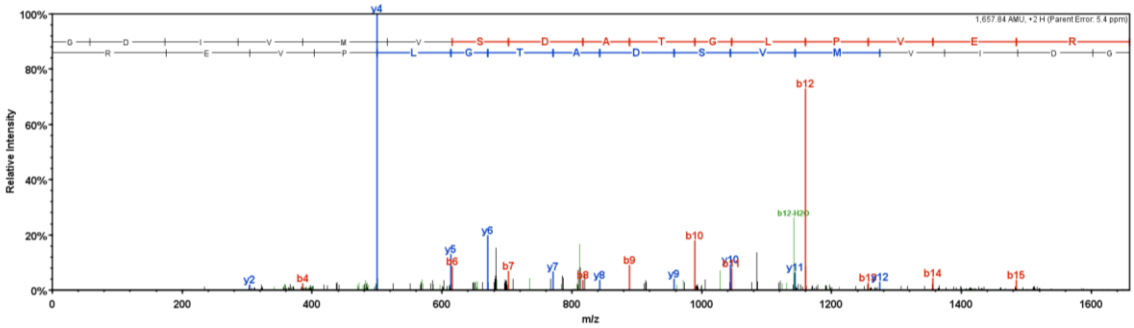

Good?	…	Sequence	Prob	Masc…	Masc…	Masc…	NTT	Modifications	Observed	Actual Mass	Charge	Delta …	Delta …	S
☑	✓	(K)EMASTSMNNTSSR(S)	95%	60.7	32.9	45.5	2		708.30	1,414.59	2	0.0081	5.8	
☑	✓	(R)GDIVMVSDATGLPVER(E)	95%	35.5	28.6	26.0	2		829.93	1,657.85	2	0.013	7.6	
☑	✓	(R)GDIVMVSDATGLPVER(E)	95%	49.6	30.9	42.3	2		829.93	1,657.84	2	0.0057	3.4	
☑	✓	(R)GDIVMVSDATGLPVER(E)	95%	51.4	30.3	40.6	2		829.93	1,657.85	2	0.013	7.9	
☑	✓	(R)GDIVMVSDATGLPVER(E)	95%	56.0	30.3	42.3	2		829.93	1,657.85	2	0.013	7.9	
☑	✓	(R)GDIVMVSDATGLPVER(E)	95%	58.8	30.6	49.2	2		829.93	1,657.84	2	0.0090	5.4	
☑	✓	(K)TLDEATHINR(S)	95%	40.5	30.5	23.8	2		585.30	1,168.59	2	0.0047	4.0	
☑	✓	(K)TLEEALEEER(G)	95%	45.0	33.1	33.4	2		609.80	1,217.59	2	0.0078	6.4	

B	B Ions	B+2H	B-NH3	B-H2O	AA	Y Ions	Y+2H	Y-NH3	Y-H2O	Y
1	130.0			112.0	E	1,415.6	708.3	1,398.6	1,397.6	13
2	261.1			243.1	M	1,286.5	643.8	1,269.5	1,268.5	12
3	332.1			314.1	A	1,155.5	578.3	1,138.5	1,137.5	11
4	419.2			401.1	S	1,084.5	542.7	1,067.4	1,066.5	10
5	520.2			502.2	T	997.4	499.2	980.4	979.4	9
6	607.2	304.1		589.2	S	896.4	448.7	879.4	878.4	8
7	738.3	369.6		720.3	M	809.4	405.2	792.3	791.3	7
8	852.3	426.7	835.3	834.3	N	678.3	339.7	661.3	660.3	6
9	966.4	483.7	949.3	948.4	N	564.3		547.2	546.3	5
10	1,067.4	534.2	1,050.4	1,049.4	T	450.2		433.2	432.2	4
11	1,154.4	577.7	1,137.4	1,136.4	S	349.2		332.2	331.2	3
12	1,241.5	621.2	1,224.5	1,223.5	S	262.2		245.1	244.1	2
13	1,415.6	708.3	1,398.6	1,397.6	R	175.1		158.1		1

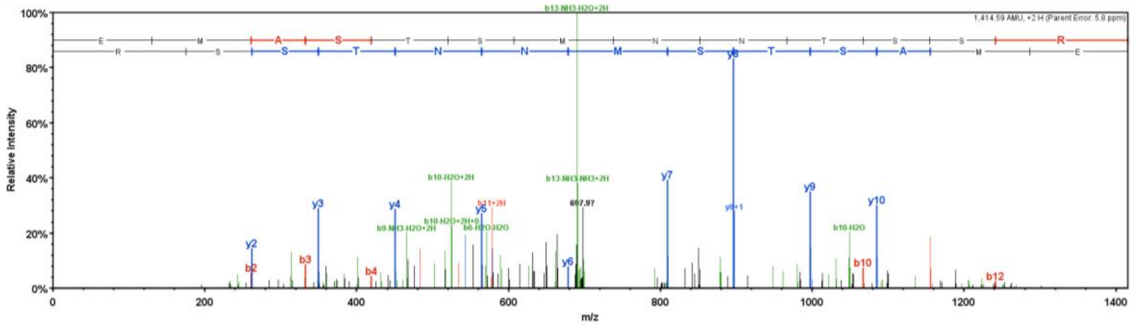

Good?	…	Sequence	Prob	Masc…	Masc…	Masc…	NTT	Modifications	Observed	Actual Mass	Charge	Delta …	Delta …	S
☑	✓	(K)EMASTSMNNTSSR(S)	95%	60.7	32.9	45.5	2		708.30	1,414.59	2	0.0081	5.8	
☑	✓	(R)GDIVMVSDATGLPVER(E)	95%	35.5	28.6	26.0	2		829.93	1,657.85	2	0.013	7.6	
☑	✓	(R)GDIVMVSDATGLPVER(E)	95%	49.6	30.9	42.3	2		829.93	1,657.84	2	0.0057	3.4	
☑	✓	(R)GDIVMVSDATGLPVER(E)	95%	51.4	30.3	40.6	2		829.93	1,657.85	2	0.013	7.9	
☑	✓	(R)GDIVMVSDATGLPVER(E)	95%	56.0	30.3	42.3	2		829.93	1,657.85	2	0.013	7.9	
☑	✓	(R)GDIVMVSDATGLPVER(E)	95%	58.8	30.6	49.2	2		829.93	1,657.84	2	0.0090	5.4	
☑	✓	(K)TLDEATHINR(S)	95%	40.5	30.5	23.8	2		585.30	1,168.59	2	0.0047	4.0	
☑	✓	(K)TLEEALEEER(G)	95%	45.0	33.1	33.4	2		609.80	1,217.59	2	0.0078	6.4	

B	B Ions	B+2H	B-NH3	B-H2O	AA	Y Ions	Y+2H	Y-NH3	Y-H2O	Y
1	102.1			84.0	T	1,169.6	585.3	1,152.6	1,151.6	10
2	215.1			197.1	L	1,068.5	534.8	1,051.5	1,050.5	9
3	330.2			312.2	D	955.5	478.2	938.4	937.4	8
4	459.2			441.2	E	840.4	420.7	823.4	822.4	7
5	530.2			512.2	A	711.4	356.2	694.4	693.4	6
6	631.3	316.2		613.3	T	640.4	320.7	623.3	622.3	5
7	768.4	384.7		750.3	H	539.3	270.2	522.3		4
8	881.4	441.2		863.4	I	402.2		385.2		3
9	995.5	498.2	978.5	977.5	N	289.2		272.1		2
10	1,169.6	585.3	1,152.6	1,151.6	R	175.1		158.1		1

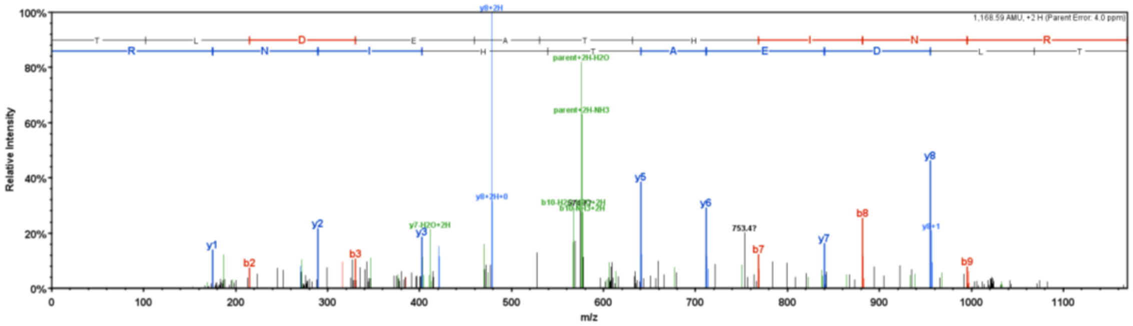

ood?	...	Sequence	Prob	Masc...	Masc...	Masc...	NTT	Modifications	Observed	Actual Mass	Charge	Delta ...	Delta ...	S
☑	✓	(K)EMASTSMNNTSSR(S)	95%	60.7	32.9	45.5	2		708.30	1,414.59	2	0.0081	5.8	
☑	✓	(R)GDIVMVSDATGLPVER(E)	95%	35.5	28.6	26.0	2		829.93	1,657.85	2	0.013	7.6	
☑	✓	(R)GDIVMVSDATGLPVER(E)	95%	49.6	30.9	42.3	2		829.93	1,657.84	2	0.0057	3.4	
☑	✓	(R)GDIVMVSDATGLPVER(E)	95%	51.4	30.3	40.6	2		829.93	1,657.85	2	0.013	7.9	
☑	✓	(R)GDIVMVSDATGLPVER(E)	95%	56.0	30.3	42.3	2		829.93	1,657.85	2	0.013	7.9	
☑	✓	(R)GDIVMVSDATGLPVER(E)	95%	58.8	30.6	49.2	2		829.93	1,657.84	2	0.0090	5.4	
☑	✓	(K)TLDEATHINR(S)	95%	40.5	30.5	23.8	2		585.30	1,168.59	2	0.0047	4.0	
☑	✓	(K)TLEEALEEER(G)	95%	45.0	33.1	33.4	2		609.80	1,217.59	2	0.0078	6.4	

B	B Ions	B+2H	B-NH3	B-H2O	AA	Y Ions	Y+2H	Y-NH3	Y-H2O	Y
1	102.1			84.0	T	1,218.6	609.8	1,201.6	1,200.6	10
2	215.1			197.1	L	1,117.5	559.3	1,100.5	1,099.5	9
3	344.2			326.2	E	1,004.5	502.7	987.4	986.4	8
4	473.2			455.2	E	875.4	438.2	858.4	857.4	7
5	544.3			526.3	A	746.4	373.7	729.3	728.4	6
6	657.3	329.2		639.3	L	675.3		658.3	657.3	5
7	786.4	393.7		768.4	E	562.2		545.2	544.2	4
8	915.4	458.2		897.4	E	433.2		416.2	415.2	3
9	1,044.5	522.7		1,026.5	E	304.2		287.1	286.2	2
10	1,218.6	609.8	1,201.6	1,200.6	R	175.1		158.1		1

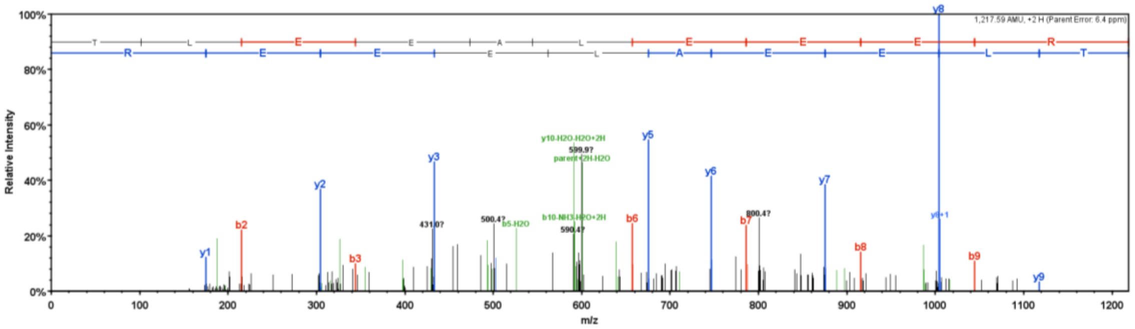

Good?	...	Sequence	Prob	Masc...	Masc...	Masc...	NTT	Modifications	Observed	Actual Mass	Charge	Delta ...	Delta ...	S
☑	✓	(R)ELIQQR(D)	95%	31.2	29.8	0.0	2		393.73	785.44	2	0.0023	2.9	
☑	✓	(K)EMASTSMNNTSSR(S)	95%	60.7	32.9	0.0	2		708.30	1,414.59	2	0.0081	5.8	
☑	✓	(R)GDIVMVSDATGLPVER(E)	95%	49.6	30.9	0.0	2		829.93	1,657.84	2	0.0057	3.4	
☑	✓	(R)GDIVMVSDATGLPVER(E)	95%	51.4	30.3	0.0	2		829.93	1,657.85	2	0.013	7.9	

B	B Ions	B+2H	B-NH3	B-H2O	AA	Y Ions	Y+2H	Y-NH3	Y-H2O	Y
1	130.0			112.0	E	786.4	393.7	769.4	768.4	6
2	243.1			225.1	L	657.4		640.4		5
3	356.2			338.2	I	544.3		527.3		4
4	484.3		467.3	466.3	Q	431.2		414.2		3
5	612.3		595.3	594.3	Q	303.2		286.2		2
6	786.4	393.7	769.4	768.4	R	175.1		158.1		1

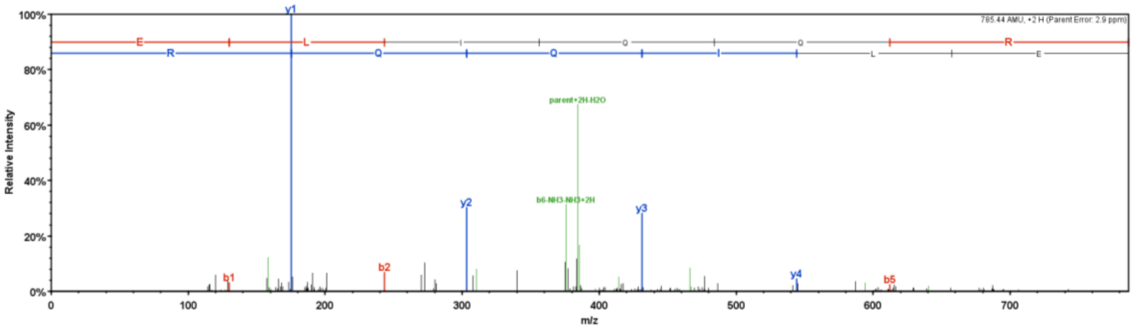

Supplementary File 2

Alignment of LmxKIN29 to homologues of other kinetoplastida:

		L. mexicana		
		Identities	Positives	Gaps
L. amazonensis	*LAMA_000589400*	602/610(99%)	606/610(99%)	0/610(0%)
L. donovani	*LdBPK_300350.1*	531/610(87%)	568/610(93%)	0/610(0%)
L. infantum	*LinJ.30.0350*	531/610(87%)	567/610(92%)	0/610(0%)
L. aethiopica	*LAEL147_000550000*	527/610(86%)	565/610(92%)	0/610(0%)
L. tropica	*LTRL590_300009000*	522/610(86%)	562/610(92%)	0/610(0%)
L. major	*LmjF.30.0350*	511/610(84%)	559/610(91%)	3/610(0%)
L. arabica	*LARLEM1108_300008900*	513/608(84%)	558/608(91%)	3/608(0%)
L. gerbilli	*LGELEM452_300008700*	512/608(84%)	558/608(91%)	3/608(0%)
L. turanica	*LTULEM423_300009100*	505/608(83%)	553/608(90%)	3/608(0%)
L. tarentolae	*LtaP30.0410.mRNA*	480/616(78%)	547/616(88%)	7/616(1%)
L. enriettii	*LENLEM3045_300009300*	467/611(76%)	543/611(88%)	6/611(0%)
L. panamensis	*LPAL13_300008900*	464/613(76%)	535/613(87%)	3/613(0%)
L. braziliensis	*LbrM.30.0390*	457/610(75%)	532/610(87%)	3/610(0%)
C. fasciculata	*CFAC1_260021700*	455/622(73%)	523/622(84%)	16/622(2%)
T. brucei	*Tb927.6.1770*	336/617(54%)	430/617(69%)	27/617(4%)
T. cruzi	*Tc00.1047053511491.60*	346/623(56%)	453/623(72%)	34/623(5%)

L. braziliensis protein aligned to *L. mexicana* protein:

```
 Score           Expect  Method                          Identities    Positives     Gaps

 964 bits(2493)  0.0     Compositional matrix adjust.    457/610(75%)  532/610(87%)  3/610(0%)

Lbra   1     MSRPQASSKSAPKTISVFCRVRPLVPHEKSHTCNNITYDPNDNRAITVNRKTTTKAGEKK  60
             MSR Q SSKSAPKTISV+CRVRP VP EK H  NNI YD  DNR ITV RK+ +K+ EK+
Lmex   1     MSRIQKSSKSAPKTISVYCRVRPPVPQEKGHNFNNIVYDDADNRTITVTRKSGSKSFEKR  60

Lbra   61    FMFNRVFQPNSTQKEVYEAFAKGAVDAAFDGQHGVLFVYGQTGSGKTFTISNDDPKSEGV  120
             + FNRVF+P  TQK+VYE FAK AVDAAFDGQHGVLFVYGQTGSGKTFTISNDDPK+EGV
Lmex   61    YFFNRVFRPTVTQKDVYETFAKNAVDAAFDGQHGVLFVYGQTGSGKTFTISNDDPKNEGV  120

Lbra   121   LQQSMRDIWAKIANDTEHDYSCSVSYVQLYNEILTDLLDEKKGKVRIQLGTEGCGDVVLV  180
             LQQSMR+IW +IA D  +DYSCSVSYVQLYNEILTDLLD+ KGKVRIQ+G EG GD+V+V
Lmex   121   LQQSMREIWDRIAKDPGNDYSCSVSYVQLYNEILTDLLDDSKGKVRIQMGLEGRGDIVMV  180

Lbra   181   SDDSGKGIEREVKDYNGTMNFFKTGLSRKEMASTAMNNTSSRSHTVFTLNISRSTKVTAV  240
             SD +G  +EREVKDY GTM FFK GL+RKEMAST+MNNTSSRSHT+FTLN+ ++ +V  V
Lmex   181   SDATGLPVEREVKDYKGTMAFFKAGLTRKEMASTSMNNTSSRSHTIFTLNVCKAQRVGTV  240
```

```
Lbra   241  TVGADSGGATVALDGRLVLCDLAGSERVSKTHAEGKTLDEATHINRSLLTLGKVVAALTA  300
            TVGA++ G T+AL+GRLVLCDLAGSERVSKTHAEGKTLDEATHINRSLLTLGKVV ALT
Lmex   241  TVGAETEGPTIALEGRLVLCDLAGSERVSKTHAEGKTLDEATHINRSLLTLGKVVTALTD  300

Lbra   301  NAQHAPFRESKLTRILQYSLMGNGNTSIVVNVSPSDENTEETFSAILFGQRASQIKQDAK  360
            NAQHAPFRESKLTRILQYSL+GNGNTSI+VN+SPSDENTEE+ S + FGQRASQIKQDAK
Lmex   301  NAQHAPFRESKLTRILQYSLLGNGNTSIIVNISPSDENTEESLSTLFFGQRASQIKQDAK  360

Lbra   361  RHEVLDYKALYLQLMADLDNKNDKTLEDALEEERGVFDDRINSLNDQVKMLTEENGMLRN  420
            RHEVLDYKALYLQLMAD+DNKNDKTLE+ALEEERGV++DRI+SLN+++K+L  EN MLRN
Lmex   361  RHEVLDYKALYLQLMADIDNKNDKTLEEALEEERGVYEDRISSLNEEMKLLNNENAMLRN  420

Lbra   421  ENKQLRQCVPADKLKTIDETPASGVPAYEGASGGGNWAKANQELREVIKARDEKLRTINE  480
            ENKQLRQ VPAD+LK IDETP+SGVP   G S  G WAKANQELRE+I+ RDEK++ I+
Lmex   421  ENKQLRQYVPADRLKLIDETPSSGVPGVNGGSISGGWAKANQELRELIQQRDEKMKVISN  480

Lbra   481  ERLRLALVVAEEQRKCFQLAQKMRAFALRYKMEREQSSRRQDALTAELASVKGTDYLSAL  540
            ER+RLALVVAEE+RKCFQLAQK+R+FA+RYK+EREQ ++RQ+ LT ELAS+KGTDYLSA+
Lmex   481  ERVRLALVVAEEKRKCFQLAQKLRSFAMRYKVEREQLTQRQEELTTELASLKGTDYLSAV  540

Lbra   541  GTFDSSMSPA---SPRGGSDAEDYNELESARQQIRSLRAERADLIMYQKKAAEAIRVLAS  597
            GTF+ ++SPA   SP+   D ED+N+ ESA+ Q+R+L AER +L++YQ KAA AIR+L
Lmex   541  GTFEPTVSPASPGSPKFARDGEDFNDAESAQAQLRALWAERTELMLYQAKAANAIRMLVK  600

Lbra   598  ERDAALRKAA  607
            ER+AA RKAA
Lmex   601  EREAAQRKAA  610
```

L. donovani protein aligned to *L. mexicana* protein:

Score	Expect	Method	Identities	Positives	Gaps
1119 bits(2894)	0.0	Compositional matrix adjust.	531/610(87%)	568/610(93%)	0/610(0%)

```
Ldon   1    MSRTQTSSKSAPKTIAVYCRVRPPVPNEKGHTFQNISYDDRDARAITVNRKSGAKSFEKK  60
            MSR Q SSKSAPKTI+VYCRVRPPVP EKGH F NI YDD D R ITV RKSG+KSFEK+
Lmex   1    MSRIQKSSKSAPKTISVYCRVRPPVPQEKGHNFNNIVYDDADNRTITVTRKSGSKSFEKR  60

Ldon   61   YLFNRVFRPNVTQKDVYENFARNAVDAAFDGQHGVLFVYGQTGSGKTFTISNDDPNNEGV  120
            Y FNRVFRP VTQKDVYE FA+NAVDAAFDGQHGVLFVYGQTGSGKTFTISNDDP NEGV
Lmex   61   YFFNRVFRPTVTQKDVYETFAKNAVDAAFDGQHGVLFVYGQTGSGKTFTISNDDPKNEGV  120

Ldon   121  LQQSMREIWNKIASDTANDYSCSVSYVQLYNEILTDLLDDKKSKVRIQMGSEGRGDVVMV  180
```

```
             LQQSMREIW++IA D  NDYSCSVSYVQLYNEILTDLLDD K KVRIQMG EGRGD+VMV
Lmex   121   LQQSMREIWDRIAKDPGNDYSCSVSYVQLYNEILTDLLDDSKGKVRIQMGLEGRGDIVMV  180

Ldon   181   SDSTGLAVEREVKDYKSTMAYFKAGLARKEMASTSMNNTSSRSHTIFTLNIVKAKKVGAV  240
             SD+TGL VEREVKDYK TMA+FKAGL RKEMASTSMNNTSSRSHTIFTLN+ KA++VG V
Lmex   181   SDATGLPVEREVKDYKGTMAFFKAGLTRKEMASTSMNNTSSRSHTIFTLNVCKAQRVGTV  240

Ldon   241   TVGAETEGPTVALEGRLVLCDLAGSERVSKTHAEGKTLDEATHINRSLLTLGKVVTALTD  300
             TVGAETEGPT+ALEGRLVLCDLAGSERVSKTHAEGKTLDEATHINRSLLTLGKVVTALTD
Lmex   241   TVGAETEGPTIALEGRLVLCDLAGSERVSKTHAEGKTLDEATHINRSLLTLGKVVTALTD  300

Ldon   301   NAQHAPFRESKLTRILQYSLMGNGNTSIIVNISPSDENTEESLSAILFGQRASQIKQDAK  360
             NAQHAPFRESKLTRILQYSL+GNGNTSIIVNISPSDENTEESLS + FGQRASQIKQDAK
Lmex   301   NAQHAPFRESKLTRILQYSLLGNGNTSIIVNISPSDENTEESLSTLFFGQRASQIKQDAK  360

Ldon   361   RHEVLDYKALYLQLMADLDNKNDKTLEEALEEERGVYEERISALDEEMKLLSDENAMLRN  420
             RHEVLDYKALYLQLMAD+DNKNDKTLEEALEEERGVYE+RIS+L+EEMKLL++ENAMLRN
Lmex   361   RHEVLDYKALYLQLMADIDNKNDKTLEEALEEERGVYEDRISSLNEEMKLLNNENAMLRN  420

Ldon   421   ENKQLRQYVPADRLKLIDETPSSGVPGANGEVASGGWAKANQDLRELVKQRDDKLKVISD  480
             ENKQLRQYVPADRLKLIDETPSSGVPG NG   SGGWAKANQ+LREL++QRD+K+KVIS+
Lmex   421   ENKQLRQYVPADRLKLIDETPSSGVPGVNGGSISGGWAKANQELRELIQQRDEKMKVISN  480

Ldon   481   ERVRLALVVAEEKRKCFQLAQKMRSFAMRYKMEREQSTQRQEELCAELAALKGTDYLSAV  540
             ERVRLALVVAEEKRKCFQLAQK+RSFAMRYK+EREQ TQRQEEL  ELA+LKGTDYLSAV
Lmex   481   ERVRLALVVAEEKRKCFQLAQKLRSFAMRYKVEREQLTQRQEELTTELASLKGTDYLSAV  540

Ldon   541   GNFDATASPGSPGSPHYPREGEEFNDAESAQAQIRALRAERTELMVYQAKAANAIRMLVK  600
             G F+ T SP SPGSP + R+GE+FNDAESAQAQ+RAL AERTELM+YQAKAANAIRMLVK
Lmex   541   GTFEPTVSPASPGSPKFARDGEDFNDAESAQAQLRALWAERTELMLYQAKAANAIRMLVK  600

Ldon   601   ERDAAQRKVA  610
             ER+AAQRK A
Lmex   601   EREAAQRKAA  610
```

L. infantum protein aligned to *L. mexicana* protein:

Score	Expect	Method	Identities	Positives	Gaps
1118 bits(2892)	0.0	Compositional matrix adjust.	531/610(87%)	567/610(92%)	0/610(0%)

```
Linf   1     MSRTQTSSKSAPKTIAVYCRVRPPVPNEKGHTFQNISYDDRDARAITVNRKSGAKSFEKK  60
             MSR Q SSKSAPKTI+VYCRVRPPVP EKGH F NI YDD D R ITV RKSG+KSFEK+
Lmex   1     MSRIQKSSKSAPKTISVYCRVRPPVPQEKGHNFNNIVYDDADNRTITVTRKSGSKSFEKR  60
```

```
Linf   61   YLFNRVFRPNVTQKDVYENFARNAVDAAFDGQHGVLFVYGQTGSGKTFTISNDDPNNEGV  120
            Y FNRVFRP VTQKDVYE FA+NAVDAAFDGQHGVLFVYGQTGSGKTFTISNDDP NEGV
Lmex   61   YFFNRVFRPTVTQKDVYETFAKNAVDAAFDGQHGVLFVYGQTGSGKTFTISNDDPKNEGV  120

Linf   121  LQQSMREIWNKIASDTANDYSCSVSYVQLYNEILTDLLDDKKSKVRIQMGSEGRGDVVMV  180
            LQQSMREIW++IA D  NDYSCSVSYVQLYNEILTDLLDD K KVRIQMG EGRGD+VMV
Lmex   121  LQQSMREIWDRIAKDPGNDYSCSVSYVQLYNEILTDLLDDSKGKVRIQMGLEGRGDIVMV  180

Linf   181  SDTTGLAVEREVKDYKSTMAYFKAGLARKEMASTSMNNTSSRSHTIFTLNIVKAKKVGAV  240
            SD TGL VEREVKDYK TMA+FKAGL RKEMASTSMNNTSSRSHTIFTLN+ KA++VG V
Lmex   181  SDATGLPVEREVKDYKGTMAFFKAGLTRKEMASTSMNNTSSRSHTIFTLNVCKAQRVGTV  240

Linf   241  TVGAETEGPTVALEGRLVLCDLAGSERVSKTHAEGKTLDEATHINRSLLTLGKVVTALTD  300
            TVGAETEGPT+ALEGRLVLCDLAGSERVSKTHAEGKTLDEATHINRSLLTLGKVVTALTD
Lmex   241  TVGAETEGPTIALEGRLVLCDLAGSERVSKTHAEGKTLDEATHINRSLLTLGKVVTALTD  300

Linf   301  NAQHAPFRESKLTRILQYSLMGNGNTSIIVNISPSDENTEESLSAILFGQRASQIKQDAK  360
            NAQHAPFRESKLTRILQYSL+GNGNTSIIVNISPSDENTEESLS + FGQRASQIKQDAK
Lmex   301  NAQHAPFRESKLTRILQYSLLGNGNTSIIVNISPSDENTEESLSTLFFGQRASQIKQDAK  360

Linf   361  RHEVLDYKALYLQLMADLDNKNDKTLEEALEEERGVYEERISALDEEMKLLSDENAMLRN  420
            RHEVLDYKALYLQLMAD+DNKNDKTLEEALEEERGVYE+RIS+L+EEMKLL++ENAMLRN
Lmex   361  RHEVLDYKALYLQLMADIDNKNDKTLEEALEEERGVYEDRISSLNEEMKLLNNENAMLRN  420

Linf   421  ENKQLRQYVPADRLKLIDETPSSGVPGANGEVASGGWAKANQDLRELVKQRDDKLKVISD  480
            ENKQLRQYVPADRLKLIDETPSSGVPG NG   SGGWAKANQ+LREL++QRD+K+KVIS+
Lmex   421  ENKQLRQYVPADRLKLIDETPSSGVPGVNGGSISGGWAKANQELRELIQQRDEKMKVISN  480

Linf   481  ERVRLALVVAEEKRKCFQLAQKMRSFAMRYKMEREQSTQRQEELCAELAALKGTDYLSAV  540
            ERVRLALVVAEEKRKCFQLAQK+RSFAMRYK+EREQ TQRQEEL  ELA+LKGTDYLSAV
Lmex   481  ERVRLALVVAEEKRKCFQLAQKLRSFAMRYKVEREQLTQRQEELTTELASLKGTDYLSAV  540

Linf   541  GNFDATASPGSPGSPHYPREGEEFNDAESAQAQIRALRAERTELMVYQAKAANAIRMLVK  600
            G F+ T SP SPGSP + R+GE+FNDAESAQAQ+RAL AERTELM+YQAKAANAIRMLVK
Lmex   541  GTFEPTVSPASPGSPKFARDGEDFNDAESAQAQLRALWAERTELMLYQAKAANAIRMLVK  600

Linf   601  ERDAAQRKVA  610
            ER+AAQRK A
Lmex   601  EREAAQRKAA  610
```

L. major protein aligned to *L. mexicana* protein:

Score	Expect	Method	Identities	Positives	Gaps

```
1076 bits(2783)  0.0     Compositional matrix adjust.   511/610(84%)  559/610(91%)  3/610(0%)

Lmaj   1     MSRTQTSSKSVPKNIAVYCRVRPPVPNEKGHTFQNISYDDSDSRAIAVARKSGTKALEKT  60
             MSR Q SSKS PK I+VYCRVRPPVP EKGH F NI YDD+D+R I V RKSG+K+ EK
Lmex   1     MSRIQKSSKSAPKTISVYCRVRPPVPQEKGHNFNNIVYDDADNRTITVTRKSGSKSFEKR  60

Lmaj   61    YLFNRVFRPTATQKDVYETFAKGAVDAAFDGQHGVLFVYGQTGSGKTFTISNDEPNNEGV  120
             Y FNRVFRPT TQKDVYETFAK AVDAAFDGQHGVLFVYGQTGSGKTFTISND+P NEGV
Lmex   61    YFFNRVFRPTVTQKDVYETFAKNAVDAAFDGQHGVLFVYGQTGSGKTFTISNDDPKNEGV  120

Lmaj   121   LQRSMRDIWNRIASDTANDYSCSVSYVQLYNEILTDLLDDAKGKVRIQMGSEGRGDVVMV  180
             LQ+SMR+IW+RIA D  NDYSCSVSYVQLYNEILTDLLDD+KGKVRIQMG EGRGD+VMV
Lmex   121   LQQSMREIWDRIAKDPGNDYSCSVSYVQLYNEILTDLLDDSKGKVRIQMGLEGRGDIVMV  180

Lmaj   181   SDSTGMAIEREVKDYKSTMACFQVGLARKEMASTSMNSTSSRSHTIFTLNIVKAKKVVAV  240
             SD+TG+ +EREVKDYK TMA F+ GL RKEMASTSMN+TSSRSHTIFTLN+ KA++V  V
Lmex   181   SDATGLPVEREVKDYKGTMAFFKAGLTRKEMASTSMNNTSSRSHTIFTLNVCKAQRVGTV  240

Lmaj   241   TVGTEAEGPTIALEGRLVLCDLAGSERVSKTHAEGKTLDEATHINRSLLTLGKVVTALTD  300
             TVG E EGPTIALEGRLVLCDLAGSERVSKTHAEGKTLDEATHINRSLLTLGKVVTALTD
Lmex   241   TVGAETEGPTIALEGRLVLCDLAGSERVSKTHAEGKTLDEATHINRSLLTLGKVVTALTD  300

Lmaj   301   NAQHAPFRESKLTRILQYSLMGNGNTSIIVNISPSDDNTEESLSAILFGQRASQIKQDAK  360
             NAQHAPFRESKLTRILQYSL+GNGNTSIIVNISPSD+NTEESLS + FGQRASQIKQDAK
Lmex   301   NAQHAPFRESKLTRILQYSLLGNGNTSIIVNISPSDENTEESLSTLFFGQRASQIKQDAK  360

Lmaj   361   RHEVLDYKALYMQLMAELDNKNDKTLEEALEEERGVYEERISALDEEMKLLSDENAMLRN  420
             RHEVLDYKALY+QLMA++DNKNDKTLEEALEEERGVYE+RIS+L+EEMKLL++ENAMLRN
Lmex   361   RHEVLDYKALYLQLMADIDNKNDKTLEEALEEERGVYEDRISSLNEEMKLLNNENAMLRN  420

Lmaj   421   ENKQLRQYVPADRLKLIDETPSSGVSGANGEVVSGGWAKVNQDLRKLVQQRDERLKVISD  480
             ENKQLRQYVPADRLKLIDETPSSGV G NG  +SGGWAK NQ+LR+L+QQRDE++KVIS+
Lmex   421   ENKQLRQYVPADRLKLIDETPSSGVPGVNGGSISGGWAKANQELRELIQQRDEKMKVISN  480

Lmaj   481   ERVRLALVVAEEKRKCFQLAQKMRSFAMRYKMEREQSTQRQEELCTELATLKGTDYLSAV  540
             ERVRLALVVAEEKRKCFQLAQK+RSFAMRYK+EREQ TQRQEEL TELA+LKGTDYLSAV
Lmex   481   ERVRLALVVAEEKRKCFQLAQKLRSFAMRYKVEREQLTQRQEELTTELASLKGTDYLSAV  540

Lmaj   541   GSFDAT---ASPGSPNYPRENEEFNDAESAQAQIRALRAERMELMVYQAKAANAIRKLVK  597
             G+F+ T   ASPGSP + R+ E+FNDAESAQAQ+RAL AER ELM+YQAKAANAIR LVK
Lmex   541   GTFEPTVSPASPGSPKFARDGEDFNDAESAQAQLRALWAERTELMLYQAKAANAIRMLVK  600

Lmaj   598   ERDAAQRKVA  607
             ER+AAQRK A
Lmex   601   EREAAQRKAA  610
```

L. aethiopica protein aligned to *L. mexicana* protein:

Score	Expect	Method	Identities	Positives	Gaps
1113 bits(2879)	0.0	Compositional matrix adjust.	527/610(86%)	565/610(92%)	0/610(0%)

```
Laet   1    MSRAQTSSKSAPKTIAVYCRVRPPVPHEKGHTFQNINYDDGDSRAIIVNRKSGTKSLEKK  60
            MSR Q SSKSAPKTI+VYCRVRPPVP EKGH F NI YDD D+R I V RKSG+KS EK+
Lmex   1    MSRIQKSSKSAPKTISVYCRVRPPVPQEKGHNFNNIVYDDADNRTITVTRKSGSKSFEKR  60

Laet   61   YFFNRVFRPNVTQKDVYETFARSAVDAAFDGQHGVLFVYGQTGSGKTFTISNDDPNNEGV  120
            YFFNRVFRP VTQKDVYETFA++AVDAAFDGQHGVLFVYGQTGSGKTFTISNDDP NEGV
Lmex   61   YFFNRVFRPTVTQKDVYETFAKNAVDAAFDGQHGVLFVYGQTGSGKTFTISNDDPKNEGV  120

Laet   121  LQQSMREIWDKVASDTANDYSCSVSYVQLYNEILTDLLDDKKGKVRIQMGSEGRGDVVMV  180
            LQQSMREIWD++A D  NDYSCSVSYVQLYNEILTDLLDD KGKVRIQMG EGRGD+VMV
Lmex   121  LQQSMREIWDRIAKDPGNDYSCSVSYVQLYNEILTDLLDDSKGKVRIQMGLEGRGDIVMV  180

Laet   181  SDSTGMAIEREVKDYKSTMAYFKGGLARKEMASTSMNNTSSRSHTVFTLNIVKAKKVVAV  240
            SD+TG+ +EREVKDYK TMA+FK GL RKEMASTSMNNTSSRSHT+FTLN+ KA++V  V
Lmex   181  SDATGLPVEREVKDYKGTMAFFKAGLTRKEMASTSMNNTSSRSHTIFTLNVCKAQRVGTV  240

Laet   241  TVGAETDGPTVALEGRLVLCDLAGSERVSKTHAEGKTLDEATHINRSLLTLGKVVTALTD  300
            TVGAET+GPT+ALEGRLVLCDLAGSERVSKTHAEGKTLDEATHINRSLLTLGKVVTALTD
Lmex   241  TVGAETEGPTIALEGRLVLCDLAGSERVSKTHAEGKTLDEATHINRSLLTLGKVVTALTD  300

Laet   301  NAQHAPFRESKLTRILQYSLLGNGNTSIIVNISPSDNNTEESLSAIFFGQRASQIKQDAK  360
            NAQHAPFRESKLTRILQYSLLGNGNTSIIVNISPSD NTEESLS +FFGQRASQIKQDAK
Lmex   301  NAQHAPFRESKLTRILQYSLLGNGNTSIIVNISPSDENTEESLSTLFFGQRASQIKQDAK  360

Laet   361  RHEVLDYKALYLQLMADMDNKNDKTLEEALEEERGVYEERISALDEEMKLLNNENAMLRN  420
            RHEVLDYKALYLQLMAD+DNKNDKTLEEALEEERGVYE+RIS+L+EEMKLLNNENAMLRN
Lmex   361  RHEVLDYKALYLQLMADIDNKNDKTLEEALEEERGVYEDRISSLNEEMKLLNNENAMLRN  420

Laet   421  ENKQLRQYVPTDRLKLIDETPSSGVSGANGEVVSGGWAKANQDLRELVKQRDEKLKVISD  480
            ENKQLRQYVP DRLKLIDETPSSGV G NG  +SGGWAKANQ+LREL++QRDEK+KVIS+
Lmex   421  ENKQLRQYVPADRLKLIDETPSSGVPGVNGGSISGGWAKANQELRELIQQRDEKMKVISN  480

Laet   481  ERVRLALVVAEEKRKCFQLAQKMRSFAMRYKMEREQSTQRQEELCAELATLKGTDYLSAV  540
            ERVRLALVVAEEKRKCFQLAQK+RSFAMRYK+EREQ TQRQEEL  ELA+LKGTDYLSAV
Lmex   481  ERVRLALVVAEEKRKCFQLAQKLRSFAMRYKVEREQLTQRQEELTTELASLKGTDYLSAV  540

Laet   541  GTFDAVASPGSPGSPNCSREGEDFNDAESAQAQIRALRAERAELMLYQAKAANAIRKLVK  600
            GTF+   SP SPGSP  +R+GEDFNDAESAQAQ+RAL AER ELMLYQAKAANAIR LVK
```

```
Lmex   541   GTFEPTVSPASPGSPKFARDGEDFNDAESAQAQLRALWAERTELMLYQAKAANAIRMLVK  600


Laet   601   ERDAAQRKVA  610
             ER+AAQRK A
Lmex   601   EREAAQRKAA  610
```

L. amazonensis protein aligned to *L. mexicana* protein:

Score	Expect	Method	Identities	Positives	Gaps
1250 bits(3235)	0.0	Compositional matrix adjust.	602/610(99%)	606/610(99%)	0/610(0%)

```
Lama   1     MSRTQTSSKSAPKTISVYCRVRPPVPQEKGHSFNNIVYDDADNRTITVTRKSGTKSFEKR  60
             MSR Q SSKSAPKTISVYCRVRPPVPQEKGH+FNNIVYDDADNRTITVTRKSG+KSFEKR
Lmex   1     MSRIQKSSKSAPKTISVYCRVRPPVPQEKGHNFNNIVYDDADNRTITVTRKSGSKSFEKR  60

Lama   61    YFFNRVFRPTVTQKDVYETFAKNAVDAAFDGQHGVLFVYGQTGSGKTFTISNDDPKNEGV  120
             YFFNRVFRPTVTQKDVYETFAKNAVDAAFDGQHGVLFVYGQTGSGKTFTISNDDPKNEGV
Lmex   61    YFFNRVFRPTVTQKDVYETFAKNAVDAAFDGQHGVLFVYGQTGSGKTFTISNDDPKNEGV  120

Lama   121   LQQSMREIWGRIAKDPGNDYSCSVSYVQLYNEILTDLLDDSKGKVRIQMGLEGRGDIVMV  180
             LQQSMREIW RIAKDPGNDYSCSVSYVQLYNEILTDLLDDSKGKVRIQMGLEGRGDIVMV
Lmex   121   LQQSMREIWDRIAKDPGNDYSCSVSYVQLYNEILTDLLDDSKGKVRIQMGLEGRGDIVMV  180

Lama   181   SDATGLPVEREVKDYKGTMAFFKAGLARKEMASTSMNNTSSRSHTIFTLNVCKAQRVGTV  240
             SDATGLPVEREVKDYKGTMAFFKAGL RKEMASTSMNNTSSRSHTIFTLNVCKAQRVGTV
Lmex   181   SDATGLPVEREVKDYKGTMAFFKAGLTRKEMASTSMNNTSSRSHTIFTLNVCKAQRVGTV  240

Lama   241   TVGAETEGPTIALEGRLVLCDLAGSERVSKTHAEGKTLDEATHINRSLLTLGKVVTALTD  300
             TVGAETEGPTIALEGRLVLCDLAGSERVSKTHAEGKTLDEATHINRSLLTLGKVVTALTD
Lmex   241   TVGAETEGPTIALEGRLVLCDLAGSERVSKTHAEGKTLDEATHINRSLLTLGKVVTALTD  300

Lama   301   NAQHAPFRESKLTRILQYSLLGNGNTSIIVNISPSDENTEESLSTLFFGQRASQIKQDAK  360
             NAQHAPFRESKLTRILQYSLLGNGNTSIIVNISPSDENTEESLSTLFFGQRASQIKQDAK
Lmex   301   NAQHAPFRESKLTRILQYSLLGNGNTSIIVNISPSDENTEESLSTLFFGQRASQIKQDAK  360

Lama   361   RHEVLDYKALYLQLMADIDNKNDKTLEEALEEERGVYEDRISSLNEEMKLLNNENAMLRN  420
             RHEVLDYKALYLQLMADIDNKNDKTLEEALEEERGVYEDRISSLNEEMKLLNNENAMLRN
Lmex   361   RHEVLDYKALYLQLMADIDNKNDKTLEEALEEERGVYEDRISSLNEEMKLLNNENAMLRN  420

Lama   421   ENKQLRQYVPADRLKLIDETPSSGVPGVNGGSISGGWAKANQELRELIQQRDEKMKVISN  480
             ENKQLRQYVPADRLKLIDETPSSGVPGVNGGSISGGWAKANQELRELIQQRDEKMKVISN
Lmex   421   ENKQLRQYVPADRLKLIDETPSSGVPGVNGGSISGGWAKANQELRELIQQRDEKMKVISN  480
```

```
Lama    481   ERVRLALVVAEEKRKCFQLAQKLRSFAMRYKVEREQLTQRQEELTTELASLKGTDYLSAV  540
              ERVRLALVVAEEKRKCFQLAQKLRSFAMRYKVEREQLTQRQEELTTELASLKGTDYLSAV
Lmex    481   ERVRLALVVAEEKRKCFQLAQKLRSFAMRYKVEREQLTQRQEELTTELASLKGTDYLSAV  540

Lama    541   GTFDPTVSPATPGSPKFARDGEDFNDAESAQAQLRALWAERTELMLYQAKAANAIRMLVK  600
              GTF+PTVSPA+PGSPKFARDGEDFNDAESAQAQLRALWAERTELMLYQAKAANAIRMLVK
Lmex    541   GTFEPTVSPASPGSPKFARDGEDFNDAESAQAQLRALWAERTELMLYQAKAANAIRMLVK  600

Lama    601   EREAAQRKAA  610
              EREAAQRKAA
Lmex    601   EREAAQRKAA  610
```

L. arabica protein aligned to *L. mexicana* protein:

```
 Score            Expect  Method                          Identities    Positives     Gaps

 1079 bits(2790)  0.0     Compositional matrix adjust.   513/608(84%)  558/608(91%)  3/608(0%)

Lara    1     MSRTQTSSKSAPKHIAVYCRVRPPVLNEKGHTFQNISYDDSDNRAITVTRKSGTKALEKR  60
              MSR Q SSKSAPK I+VYCRVRPPV  EKGH F NI YDD+DNR ITVTRKSG+K+ EKR
Lmex    1     MSRIQKSSKSAPKTISVYCRVRPPVPQEKGHNFNNIVYDDADNRTITVTRKSGSKSFEKR  60

Lara    61    YLFNRVFRPNATQKDVYETFAKNAVDAAFDGQHGVLFVYGQTGSGKTFTISNDDPNNEGV  120
              Y FNRVFRP  TQKDVYETFAKNAVDAAFDGQHGVLFVYGQTGSGKTFTISNDDP NEGV
Lmex    61    YFFNRVFRPTVTQKDVYETFAKNAVDAAFDGQHGVLFVYGQTGSGKTFTISNDDPKNEGV  120

Lara    121   LQQSMREIWNKIASDTANDYSCSVSYVQLYNEILTDLLDDAKSKVRIQMGSEGRGDVVMV  180
              LQQSMREIW++IA D  NDYSCSVSYVQLYNEILTDLLDD+K KVRIQMG EGRGD+VMV
Lmex    121   LQQSMREIWDRIAKDPGNDYSCSVSYVQLYNEILTDLLDDSKGKVRIQMGLEGRGDIVMV  180

Lara    181   SDSTGMAIEREVKDFKSTMACFKVGLARKEMASTSMNNTSSRSHTIFTLNIVKAKKVVAV  240
              SD+TG+ +EREVKD+K TMA FK GL RKEMASTSMNNTSSRSHTIFTLN+ KA++V  V
Lmex    181   SDATGLPVEREVKDYKGTMAFFKAGLTRKEMASTSMNNTSSRSHTIFTLNVCKAQRVGTV  240

Lara    241   TVGTETEGPTVALEGRLVLCDLAGSERVSKTRAEGKTLDEATHINRSLLTLGKVVTALTD  300
              TVG ETEGPT+ALEGRLVLCDLAGSERVSKT AEGKTLDEATHINRSLLTLGKVVTALTD
Lmex    241   TVGAETEGPTIALEGRLVLCDLAGSERVSKTHAEGKTLDEATHINRSLLTLGKVVTALTD  300

Lara    301   NAQHAPFRESKLTRILQYSLMGNGNTSIIVNISPSDDNTEESLSAILFGQRASQIKQDAK  360
              NAQHAPFRESKLTRILQYSL+GNGNTSIIVNISPSD+NTEESLS + FGQRASQIKQDAK
Lmex    301   NAQHAPFRESKLTRILQYSLLGNGNTSIIVNISPSDENTEESLSTLFFGQRASQIKQDAK  360

Lara    361   RHEVLDYKALYMQLMADLDNKNDKTLEEALEEERGVYEERISALDEEMKLLSDENAMLRN  420
```

```
             RHEVLDYKALY+QLMAD+DNKNDKTLEEALEEERGVYE+RIS+L+EEMKLL++ENAMLRN
Lmex   361   RHEVLDYKALYLQLMADIDNKNDKTLEEALEEERGVYEDRISSLNEEMKLLNNENAMLRN  420

Lara   421   ENRQLRQYVPADRLKLIDETPCSGVSGANGGVVSGGWAKVNQDLRELVQQRDERLKVISD  480
             EN+QLRQYVPADRLKLIDETP SGV G NGG +SGGWAK NQ+LREL+QQRDE++KVIS+
Lmex   421   ENKQLRQYVPADRLKLIDETPSSGVPGVNGGSISGGWAKANQELRELIQQRDEKMKVISN  480

Lara   481   ERVRLALVVAEEKRKCFQLAQKMRSFAMRYKMEREQSTQRQEELCAELATLKGTDYLSAV  540
             ERVRLALVVAEEKRKCFQLAQK+RSFAMRYK+EREQ TQRQEEL  ELA+LKGTDYLSAV
Lmex   481   ERVRLALVVAEEKRKCFQLAQKLRSFAMRYKVEREQLTQRQEELTTELASLKGTDYLSAV  540

Lara   541   GNFDATL---SPGSPNFPRENEEFNEAESAQAEIRALRAERRELMVYQAKAANAIRKLVK  597
             G F+ T+   SPGSP F R+ E+FN+AESAQA++RAL AER ELM+YQAKAANAIR LVK
Lmex   541   GTFEPTVSPASPGSPKFARDGEDFNDAESAQAQLRALWAERTELMLYQAKAANAIRMLVK  600

Lara   598   ERDAAQRK  605
             ER+AAQRK
Lmex   601   EREAAQRK  608
```

L. enriettii protein aligned to *L. mexicana* protein:

Score	Expect	Method	Identities	Positives	Gaps
991 bits(2563)	0.0	Compositional matrix adjust.	467/611(76%)	543/611(88%)	6/611(0%)

```
Lenr   1     MSRAQTTSKTAPKTVSVFCRVRPPVPQEKGHTFDSISYDEKDNRAIVVNRKSGSRTVEKK  60
             MSR Q +SK+APKT+SV+CRVRPPVPQEKGH F++I YD+ DNR I V RKSGS++ EK+
Lmex   1     MSRIQKSSKSAPKTISVYCRVRPPVPQEKGHNFNNIVYDDADNRTITVTRKSGSKSFEKR  60

Lenr   61    YFFNRVFRPTVSQKEVYECFAKNAVEAAFDGQHGVLFVYGQTGSGKTFTISNDDPQNEGV  120
             YFFNRVFRPTV+QK+VYE FAKNAV+AAFDGQHGVLFVYGQTGSGKTFTISNDDP+NEGV
Lmex   61    YFFNRVFRPTVTQKDVYETFAKNAVDAAFDGQHGVLFVYGQTGSGKTFTISNDDPKNEGV  120

Lenr   121   LQQAMKAIWTKIAEDKANDYTCSVSYVQLYNEILTDLLDDQKGKVRIQMGTEGRGDVLMV  180
             LQQ+M+ IW +IA+D  NDY+CSVSYVQLYNEILTDLLDD KGKVRIQMG EGRGD++MV
Lmex   121   LQQSMREIWDRIAKDPGNDYSCSVSYVQLYNEILTDLLDDSKGKVRIQMGLEGRGDIVMV  180

Lenr   181   SDATGLGIEREVTDYKTTMSYFKTGLSRKEMASTSMNNTSSRSHTVFTLNINKSKKVGAV  240
             SDATGL +EREV DYK TM++FK GL+RKEMASTSMNNTSSRSHT+FTLN+ K+++VG V
Lmex   181   SDATGLPVEREVKDYKGTMAFFKAGLTRKEMASTSMNNTSSRSHTIFTLNVCKAQRVGTV  240

Lenr   241   TVGAESEGPAVALEGRLVLCDLAGSERVSKTHAEGKTLGEATHINRSLLTLGKVVVALTE  300
             TVGAE+EGP +ALEGRLVLCDLAGSERVSKTHAEGKTL EATHINRSLLTLGKVV ALT+
Lmex   241   TVGAETEGPTIALEGRLVLCDLAGSERVSKTHAEGKTLDEATHINRSLLTLGKVVTALTD  300
```

```
Lenr   301   NAQHAPFRESKLTRILQYSLMGNGNTSLVVNISPSDSNTEESLSAIMFGQRASQIKQDAK  360
             NAQHAPFRESKLTRILQYSL+GNGNTS++VNISPSD NTEESLS + FGQRASQIKQDAK
Lmex   301   NAQHAPFRESKLTRILQYSLLGNGNTSIIVNISPSDENTEESLSTLFFGQRASQIKQDAK  360

Lenr   361   RHEVLDYKALYLQLMADMDNKNDKTLEDALEEERSVYEDRISNLNDQIKLLSNENSMLRN  420
             RHEVLDYKALYLQLMAD+DNKNDKTLE+ALEEER VYEDRIS+LN+++KLL+NEN+MLRN
Lmex   361   RHEVLDYKALYLQLMADIDNKNDKTLEEALEEERGVYEDRISSLNEEMKLLNNENAMLRN  420

Lenr   421   ENSMLRSAVPPEKLKMIDETPASGVAVPDGAAGGGGGGNWAKANQELREMIKLRDEKLRA  480
             EN  LR  VP ++LK+IDETP+SGV    G  GG   G WAKANQELRE+I+ RDEK++
Lmex   421   ENKQLRQYVPADRLKLIDETPSSGVP---GVNGGSISGGWAKANQELRELIQQRDEKMKV  477

Lenr   481   ISDERVRLALVLAEEQRKCFQLAQKMRSFASRYKLERAQAIRRQEELCAELAAVKGTDYL  540
             IS+ERVRLALV+AEE+RKCFQLAQK+RSFA RYK+ER Q  +RQEEL  ELA++KGTDYL
Lmex   478   ISNERVRLALVVAEEKRKCFQLAQKLRSFAMRYKVEREQLTQRQEELTTELASLKGTDYL  537

Lenr   541   SALGNFDAAL---TAGSARTSREAEDFSDNDRAQALIRALRAERMELIVYQAKAASAIRM  597
             SA+G F+  +   + GS + +R+ EDF+D + AQA +RAL AER EL++YQAKAA+AIRM
Lmex   538   SAVGTFEPTVSPASPGSPKFARDGEDFNDAESAQAQLRALWAERTELMLYQAKAANAIRM  597

Lenr   598   LVKERDAALRE  608
             LVKER+AA R+
Lmex   598   LVKEREAAQRK  608
```

L. gerbilli protein aligned to *L. mexicana* protein:

Score	Expect	Method	Identities	Positives	Gaps
1077 bits(2785)	0.0	Compositional matrix adjust.	512/608(84%)	558/608(91%)	3/608(0%)

```
Lger   1     MSRTQTSSKSAPKNIAVYCRVRPPVLNEKGHTFQNISYDESDNRAITVTRKSGTKALEKR  60
             MSR Q SSKSAPK I+VYCRVRPPV  EKGH F NI YD++DNR ITVTRKSG+K+ EKR
Lmex   1     MSRIQKSSKSAPKTISVYCRVRPPVPQEKGHNFNNIVYDDADNRTITVTRKSGSKSFEKR  60

Lger   61    YLFNRVFRPNATQKDVYETFAKNAVDAAFDGQHGVLFVYGQTGSGKTFTISNDDPNNEGV  120
             Y FNRVFRP  TQKDVYETFAKNAVDAAFDGQHGVLFVYGQTGSGKTFTISNDDP NEGV
Lmex   61    YFFNRVFRPTVTQKDVYETFAKNAVDAAFDGQHGVLFVYGQTGSGKTFTISNDDPKNEGV  120

Lger   121   LQQSMREIWSKIASDTANDYSCSVSYVQLYNEILTDLLDDAKSKVRIQMGSEGRGDVVMV  180
             LQQSMREIW +IA D  NDYSCSVSYVQLYNEILTDLLDD+K KVRIQMG EGRGD+VMV
Lmex   121   LQQSMREIWDRIAKDPGNDYSCSVSYVQLYNEILTDLLDDSKGKVRIQMGLEGRGDIVMV  180

Lger   181   SDSTGVAIEREVKDYKSTMACFKAGLARKEMASTSMNNTSSRSHTIFTLNIVKAKKVVAV  240
             SD+TG+ +EREVKDYK TMA FKAGL RKEMASTSMNNTSSRSHTIFTLN+ KA++V  V
```

```
Lmex   181  SDATGLPVEREVKDYKGTMAFFKAGLTRKEMASTSMNNTSSRSHTIFTLNVCKAQRVGTV  240

Lger   241  TVGTETEGPTVALEGRLVLCDLAGSERVSKTRAEGKTLDEATHINRSLLTLGKVVTALTD  300
            TVG ETEGPT+ALEGRLVLCDLAGSERVSKT AEGKTLDEATHINRSLLTLGKVVTALTD
Lmex   241  TVGAETEGPTIALEGRLVLCDLAGSERVSKTHAEGKTLDEATHINRSLLTLGKVVTALTD  300

Lger   301  NAQHAPFRESKLTRILQYSLMGNGNTSIIVNISPSDDNTEESLSAILFGQRASQIKQDAK  360
            NAQHAPFRESKLTRILQYSL+GNGNTSIIVNISPSD+NTEESLS + FGQRASQIKQDAK
Lmex   301  NAQHAPFRESKLTRILQYSLLGNGNTSIIVNISPSDENTEESLSTLFFGQRASQIKQDAK  360

Lger   361  RHEVLDYKALYMQLMADLDNKNDRTLEEALEEERGVYEERISALDEEMKLLSDENAMLRN  420
            RHEVLDYKALY+QLMAD+DNKND+TLEEALEEERGVYE+RIS+L+EEMKLL++ENAMLRN
Lmex   361  RHEVLDYKALYLQLMADIDNKNDKTLEEALEEERGVYEDRISSLNEEMKLLNNENAMLRN  420

Lger   421  ENRQLRQYVPADRLKLIDETPFSGVSGANGGVVSGGWAKVNQDLRELVQQRDERLKVISD  480
            EN+QLRQYVPADRLKLIDETP SGV G NGG +SGGWAK NQ+LREL+QQRDE++KVIS+
Lmex   421  ENKQLRQYVPADRLKLIDETPSSGVPGVNGGSISGGWAKANQELRELIQQRDEKMKVISN  480

Lger   481  ERVRLALVVAEEKRKCFQLAQKMRSFAMRYKMEREQSTQRQEELCAELATLKGTDYLSAV  540
            ERVRLALVVAEEKRKCFQLAQK+RSFAMRYK+EREQ TQRQEEL  ELA+LKGTDYLSAV
Lmex   481  ERVRLALVVAEEKRKCFQLAQKLRSFAMRYKVEREQLTQRQEELTTELASLKGTDYLSAV  540

Lger   541  GNFDAT---SNPGSPNFPRENEEFNEAESAQAEIRALRAERRELMVYQAKAANAIRKLVK  597
            G F+ T   ++PGSP F R+ E+FN+AESAQA++RAL AER ELM+YQAKAANAIR LVK
Lmex   541  GTFEPTVSPASPGSPKFARDGEDFNDAESAQAQLRALWAERTELMLYQAKAANAIRMLVK  600

Lger   598  ERDAAQRK  605
            ER+AAQRK
Lmex   601  EREAAQRK  608
```

L. panamensis protein aligned to *L. mexicana* protein:

Score	Expect	Method	Identities	Positives	Gaps
978 bits(2527)	0.0	Compositional matrix adjust.	464/613(76%)	535/613(87%)	3/613(0%)

```
Lpan   1    MSRPQASSKSAPKTISVFCRVRPLVPHEKSHTCNNITYDPNDNRAITVNRKTTTKAGEKK  60
            MSR Q SSKSAPKTISV+CRVRP VP EK H  NNI YD  DNR ITV RK+ +K+ EK+
Lmex   1    MSRIQKSSKSAPKTISVYCRVRPPVPQEKGHNFNNIVYDDADNRTITVTRKSGSKSFEKR  60

Lpan   61   FMFNRVFQPNSTQKEVYEAFAKNAVDAAFDGQHGVLFVYGQTGSGKTFTISNDDPKNEGV  120
            + FNRVF+P  TQK+VYE FAKNAVDAAFDGQHGVLFVYGQTGSGKTFTISNDDPKNEGV
Lmex   61   YFFNRVFRPTVTQKDVYETFAKNAVDAAFDGQHGVLFVYGQTGSGKTFTISNDDPKNEGV  120
```

```
Lpan   121  LQQSMRDIWTKIANDTEHDYSCSVSYVQLYNEILTDLLDEKKGKVRIQLGTEGCGDVVLV  180
            LQQSMR+IW +IA D  +DYSCSVSYVQLYNEILTDLLD+ KGKVRIQ+G EG GD+V+V
Lmex   121  LQQSMREIWDRIAKDPGNDYSCSVSYVQLYNEILTDLLDDSKGKVRIQMGLEGRGDIVMV  180

Lpan   181  SDGSGKGIEREVKDYNGTMNFFKTGLSRKEMASTAMNNTSSRSHTVFTLNISRCTKVTAV  240
            SD +G   +EREVKDY GTM FFK GL+RKEMAST+MNNTSSRSHT+FTLN+ +  +V  V
Lmex   181  SDATGLPVEREVKDYKGTMAFFKAGLTRKEMASTSMNNTSSRSHTIFTLNVCKAQRVGTV  240

Lpan   241  TVGGDSGGATVALDGRLVLCDLAGSERVSKTHAEGKTLDEATHINRSLLTLGKVVAALTA  300
            TVG ++ G T+AL+GRLVLCDLAGSERVSKTHAEGKTLDEATHINRSLLTLGKVV ALT
Lmex   241  TVGAETEGPTIALEGRLVLCDLAGSERVSKTHAEGKTLDEATHINRSLLTLGKVVTALTD  300

Lpan   301  NAQHAPFRESKLTRILQYSLMGNGNTSIVVNVSPSDENTEETFSAILFGQRASQIKQDAK  360
            NAQHAPFRESKLTRILQYSL+GNGNTSI+VN+SPSDENTEE+ S + FGQRASQIKQDAK
Lmex   301  NAQHAPFRESKLTRILQYSLLGNGNTSIIVNISPSDENTEESLSTLFFGQRASQIKQDAK  360

Lpan   361  RHEVLDYKALYLQLMADLDNKNDKTLEDALEEERGVFDDRINSLNDQVKILTEENGMLRN  420
            RHEVLDYKALYLQLMAD+DNKNDKTLE+ALEEERGV++DRI+SLN+++K+L  EN MLRN
Lmex   361  RHEVLDYKALYLQLMADIDNKNDKTLEEALEEERGVYEDRISSLNEEMKLLNNENAMLRN  420

Lpan   421  ENKQLRQCVPPDKLKTIDETPASGVPAYEGASGGGSWAKANQELREVIKSRDEKLRTINE  480
            ENKQLRQ VP D+LK IDETP+SGVP   G S  G WAKANQELRE+I+ RDEK++ I+
Lmex   421  ENKQLRQYVPADRLKLIDETPSSGVPGVNGGSISGGWAKANQELRELIQQRDEKMKVISN  480

Lpan   481  ERLRLALVVAEEQRKCFQLAQKMRAFALRYKMEREQSSRRQDALTAELASVKGTDYLSAL  540
            ER+RLALVVAEE+RKCFQLAQK+R+FA+RYK+EREQ ++RQ+ LT ELAS+KGTDYLSA+
Lmex   481  ERVRLALVVAEEKRKCFQLAQKLRSFAMRYKVEREQLTQRQEELTTELASLKGTDYLSAV  540

Lpan   541  GTFDSSMSPASPGSPGSPRGGSDGEDYNELESARQQIRALRAERTDLIMYQKKAAEAIRV  600
            GTF+ ++SPASPGSP   R   DGED+N+ ESA+ Q+RAL AERT+L++YQ KAA AIR+
Lmex   541  GTFEPTVSPASPGSPKFAR---DGEDFNDAESAQAQLRALWAERTELMLYQAKAANAIRM  597

Lpan   601  LASERDAALRKAA  613
            L  ER+AA RKAA
Lmex   598  LVKEREAAQRKAA  610
```

L. tropica protein aligned to *L. mexicana* protein:

Score	Expect	Method	Identities	Positives	Gaps
1100 bits(2845)	0.0	Compositional matrix adjust.	522/610(86%)	562/610(92%)	0/610(0%)

```
Ltro   1    MSRTQTSSKSAPKTIAVYCRVRPPVPHEKGDTFQNISYDDNDSRAITVNRKSGTKSIEKR  60
            MSR Q SSKSAPKTI+VYCRVRPPVP EKG   F NI YDD D+R ITV RKSG+KS EKR
```

```
Lmex   1    MSRIQKSSKSAPKTISVYCRVRPPVPQEKGHNFNNIVYDDADNRTITVTRKSGSKSFEKR  60


Ltro   61   YLFNRVFRPNVTQKDVYETFARNAVEAAFDGQHGVLFVYGQTGSGKTFTISNDDPNNEGV  120
            Y FNRVFRP VTQKDVYETFA+NAV+AAFDGQHGVLFVYGQTGSGKTFTISNDDP NEGV
Lmex   61   YFFNRVFRPTVTQKDVYETFAKNAVDAAFDGQHGVLFVYGQTGSGKTFTISNDDPKNEGV  120


Ltro   121  LQQSMREIWNKVTSDTANDYSCSVSYVQLYNEILTDLLDDKKGKVRIQMGSEGRGDIVMV  180
            LQQSMREIW+++  D  NDYSCSVSYVQLYNEILTDLLDD KGKVRIQMG EGRGDIVMV
Lmex   121  LQQSMREIWDRIAKDPGNDYSCSVSYVQLYNEILTDLLDDSKGKVRIQMGLEGRGDIVMV  180


Ltro   181  SDSTGTAIEREVKDYKSTMAYFKAGLARKEMASTSMNNTSSRSHTVFTLSIVKAKKVVAV  240
            SD+TG  +EREVKDYK TMA+FKAGL RKEMASTSMNNTSSRSHT+FTL++ KA++V  V
Lmex   181  SDATGLPVEREVKDYKGTMAFFKAGLTRKEMASTSMNNTSSRSHTIFTLNVCKAQRVGTV  240


Ltro   241  TVGAEADGPTVALEGRLVLCDLAGSERVSKTHAEGKTLDEATHINRSLLTLGKVVTALTD  300
            TVGAE +GPT+ALEGRLVLCDLAGSERVSKTHAEGKTLDEATHINRSLLTLGKVVTALTD
Lmex   241  TVGAETEGPTIALEGRLVLCDLAGSERVSKTHAEGKTLDEATHINRSLLTLGKVVTALTD  300


Ltro   301  KAQHAPFRESKLTRILQYSLLGNGNTSIIVNISPSDNNTEESLSAIFFGQRASQIKQDAK  360
             AQHAPFRESKLTRILQYSLLGNGNTSIIVNISPSD NTEESLS +FFGQRASQIKQDAK
Lmex   301  NAQHAPFRESKLTRILQYSLLGNGNTSIIVNISPSDENTEESLSTLFFGQRASQIKQDAK  360


Ltro   361  RHEVLDYKALYLQLMADMDNKNDKTLEEALEEERGVYEERISALDEEMKLLNNENVMLRN  420
            RHEVLDYKALYLQLMAD+DNKNDKTLEEALEEERGVYE+RIS+L+EEMKLLNNEN MLRN
Lmex   361  RHEVLDYKALYLQLMADIDNKNDKTLEEALEEERGVYEDRISSLNEEMKLLNNENAMLRN  420


Ltro   421  ENKQLRQYVPADRLKLIDETPSSGVHGANGEVVSGGWAKANQDLRELVRQRDEKLKVISD  480
            ENKQLRQYVPADRLKLIDETPSSGV G NG  +SGGWAKANQ+LREL++QRDEK+KVIS+
Lmex   421  ENKQLRQYVPADRLKLIDETPSSGVPGVNGGSISGGWAKANQELRELIQQRDEKMKVISN  480


Ltro   481  ERVRLALVVAEEKRKCFQLAQKMRSFAMRYKMEREQSTQRQEELCAELATLKGTDYLSAV  540
            ERVRLALVVAEEKRKCFQLAQK+RSFAMRYK+EREQ TQRQEEL  ELA+LKGTDYLSAV
Lmex   481  ERVRLALVVAEEKRKCFQLAQKLRSFAMRYKVEREQLTQRQEELTTELASLKGTDYLSAV  540


Ltro   541  GTFDAAASPGSPGSPNYSREGEDFNDAESAQAQIRALRAERAELMVYQAKAANAIRRLVE  600
            GTF+   SP SPGSP ++R+GEDFNDAESAQAQ+RAL AER ELM+YQAKAANAIR LV+
Lmex   541  GTFEPTVSPASPGSPKFARDGEDFNDAESAQAQLRALWAERTELMLYQAKAANAIRMLVK  600


Ltro   601  ERDAAQRKVA  610
            ER+AAQRK A
Lmex   601  EREAAQRKAA  610
```

L. turanica protein aligned to *L. mexicana* protein:

Score	Expect	Method	Identities	Positives	Gaps
1060 bits(2741)	0.0	Compositional matrix adjust.	505/608(83%)	553/608(90%)	3/608(0%)

```
Ltur  1    MSRTQTSSKSAPKNIAVYCRVRPPVLNEKGHTFHNISYDGNDSRAITVTRKSGTKALEKR  60
           MSR Q SSKSAPK I+VYCRVRPPV  EKGH F+NI YD  D+R ITVTRKSG+K+ EKR
Lmex  1    MSRIQKSSKSAPKTISVYCRVRPPVPQEKGHNFNNIVYDDADNRTITVTRKSGSKSFEKR  60

Ltur  61   YLFNRVFRPNATQKDVYETFAKNAVEAAFDGQHGVLFVYGQTGSGKTFTISNDDPNNEGV  120
           Y FNRVFRP  TQKDVYETFAKNAV+AAFDGQHGVLFVYGQTGSGKTFTISNDDP NEGV
Lmex  61   YFFNRVFRPTVTQKDVYETFAKNAVDAAFDGQHGVLFVYGQTGSGKTFTISNDDPKNEGV  120

Ltur  121  LQQSMREIWSKIASDTANDYSCSVSYVQLYNEILTDLLDDAKSKVRIQIGPEGRGDVVMV  180
           LQQSMREIW +IA D  NDYSCSVSYVQLYNEILTDLLDD+K KVRIQ+G EGRGD+VMV
Lmex  121  LQQSMREIWDRIAKDPGNDYSCSVSYVQLYNEILTDLLDDSKGKVRIQMGLEGRGDIVMV  180

Ltur  181  SDSTGMAIEREVKDFKSTMACFKAGLARKEMASTSMNNTSSRSHTIFTLNIVKAKKVVAV  240
           SD+TG+ +EREVKD+K TMA FKAGL RKEMASTSMNNTSSRSHTIFTLN+ KA++V  V
Lmex  181  SDATGLPVEREVKDYKGTMAFFKAGLTRKEMASTSMNNTSSRSHTIFTLNVCKAQRVGTV  240

Ltur  241  TVGTETEGPTVALEGRLVLCDLAGSERVCKTRAEGKTLDEATHINRSLLTLGKVVTALTD  300
           TVG ETEGPT+ALEGRLVLCDLAGSERV KT AEGKTLDEATHINRSLLTLGKVVTALTD
Lmex  241  TVGAETEGPTIALEGRLVLCDLAGSERVSKTHAEGKTLDEATHINRSLLTLGKVVTALTD  300

Ltur  301  NAQHAPFRESKLTRILQYSLMGNGNTSIIVNISPSDDNTEESLSAILFGQRASQIKQDAK  360
           NAQHAPFRESKLTRILQYSL+GNGNTSIIVNISPSD+NTEESLS + FGQRASQIKQDAK
Lmex  301  NAQHAPFRESKLTRILQYSLLGNGNTSIIVNISPSDENTEESLSTLFFGQRASQIKQDAK  360

Ltur  361  RHEVLDYKALYMQLMADLDSKNDKTLEEALEEERGVYEERISALDEEMKLLSDENAMLRN  420
           RHEVLDYKALY+QLMAD+D+KNDKTLEEALEEERGVYE+RIS+L+EEMKLL++ENAMLRN
Lmex  361  RHEVLDYKALYLQLMADIDNKNDKTLEEALEEERGVYEDRISSLNEEMKLLNNENAMLRN  420

Ltur  421  ENKQLRQYVPADRLKLIDETPCSGVSGANGGVVSGGWAKVNQDLRELVQQRDARLKVISE  480
           ENKQLRQYVPADRLKLIDETP SGV G NGG +SGGWAK NQ+LREL+QQRD ++KVIS
Lmex  421  ENKQLRQYVPADRLKLIDETPSSGVPGVNGGSISGGWAKANQELRELIQQRDEKMKVISN  480

Ltur  481  ERVRLALVVAEEKRKCFQLAQKMRSFAIRYKMEREQSTQRQEELSAELATLKGTDYLSAV  540
           ERVRLALVVAEEKRKCFQLAQK+RSFA+RYK+EREQ TQRQEEL+ ELA+LKGTDYLSAV
Lmex  481  ERVRLALVVAEEKRKCFQLAQKLRSFAMRYKVEREQLTQRQEELTTELASLKGTDYLSAV  540

Ltur  541  GNFDAT---SSPGSPNFPRENEEFNEVESAQAEIRALRAERRELMVYQAKAANAIRKLVS  597
           G F+ T   +SPGSP F R+ E+FN+ ESAQA++RAL AER ELM+YQAKAANAIR LV
Lmex  541  GTFEPTVSPASPGSPKFARDGEDFNDAESAQAQLRALWAERTELMLYQAKAANAIRMLVK  600

Ltur  598  ERDAAQRK  605
           ER+AAQRK
```

```
Lmex   601  EREAAQRK  608
```

L. tarentolae protein aligned to *L. mexicana* protein:

```
 Score            Expect  Method                          Identities    Positives     Gaps

 1011 bits(2615)  0.0     Compositional matrix adjust.   480/616(78%)  547/616(88%)  7/616(1%)

Ltar   1    MSRTQANNKSAPKTISVYCRVRPPVPSEKGHTFQNIAYDDGDDRAIVVSRKSGTKSLEKR  60
            MSR Q ++KSAPKTISVYCRVRPPVP EKGH F NI YDD D+R I V+RKSG+KS EKR
Lmex   1    MSRIQKSSKSAPKTISVYCRVRPPVPQEKGHNFNNIVYDDADNRTITVTRKSGSKSFEKR  60

Ltar   61   FLFNRVFQPNATQREVYDTFAKSAVEAAFDGQQGVLFVYGQTGSGKTFTISSDDPNNEGV  120
            + FNRVF+P  TQ++VY+TFAK+AV+AAFDGQ GVLFVYGQTGSGKTFTIS+DDP NEGV
Lmex   61   YFFNRVFRPTVTQKDVYETFAKNAVDAAFDGQHGVLFVYGQTGSGKTFTISNDDPKNEGV  120

Ltar   121  LQQSVKEIWKKIANDPTNDYSCSVSYVQLYNEILTDLLDEQKGKVRIQIGSEGRGDVVMV  180
            LQQS++EIW +IA DP NDYSCSVSYVQLYNEILTDLLD+ KGKVRIQ+G EGRGD+VMV
Lmex   121  LQQSMREIWDRIAKDPGNDYSCSVSYVQLYNEILTDLLDDSKGKVRIQMGLEGRGDIVMV  180

Ltar   181  SDSTGLAIEREVKDMQSTMSFFKAGLTRKEMGSTSMNDRSSRSHTVFTLHIVKSKKVGTV  240
            SD+TGL +EREVKD + TM+FFKAGLTRKEM STSMN+ SSRSHT+FTL++ K+++VGTV
Lmex   181  SDATGLPVEREVKDYKGTMAFFKAGLTRKEMASTSMNNTSSRSHTIFTLNVCKAQRVGTV  240

Ltar   241  TVGSETEGQVVALEGRLVLCDLAGSERVSKTHAEGKTLDEAMHINRSLLTLGKVVNALTD  300
            TVG+ETEG  +ALEGRLVLCDLAGSERVSKTHAEGKTLDEA HINRSLLTLGKVV ALTD
Lmex   241  TVGAETEGPTIALEGRLVLCDLAGSERVSKTHAEGKTLDEATHINRSLLTLGKVVTALTD  300

Ltar   301  NAQHAPFRESKLTRILQYSLMGNGNTSIIVNIGPSDSNTEESLSAIVFGQRASQIKQDAK  360
            NAQHAPFRESKLTRILQYSL+GNGNTSIIVNI PSD NTEESLS + FGQRASQIKQDAK
Lmex   301  NAQHAPFRESKLTRILQYSLLGNGNTSIIVNISPSDENTEESLSTLFFGQRASQIKQDAK  360

Ltar   361  RHEVLNYKALYLQLMADLDNKNDRTLEAALEEERAVYEDRVAALVEEMRLLNDENSMLRN  420
            RHEVL+YKALYLQLMAD+DNKND+TLE ALEEER VYEDR+++L EEM+LLN+EN+MLRN
Lmex   361  RHEVLDYKALYLQLMADIDNKNDKTLEEALEEERGVYEDRISSLNEEMKLLNNENAMLRN  420

Ltar   421  ENNRLRHYVPPDKLKMIDQIPASGVSGASEAG-SGGWAKANQELRELVRQRDEKLKVISD  479
            EN +LR YVP D+LK+ID+ P+SGV G +    SGGWAKANQELREL++QRDEK+KVIS+
Lmex   421  ENKQLRQYVPADRLKLIDETPSSGVPGVNGGSISGGWAKANQELRELIQQRDEKMKVISN  480

Ltar   480  ERVRLALVVAEEQRKCFQLAQKMRSFAMRYKMERELSTQRQEELSAELAALKGTEYLSTL  539
            ERVRLALVVAEE+RKCFQLAQK+RSFAMRYK+ERE  TQRQEEL+ ELA+LKGT+YLS +
Lmex   481  ERVRLALVVAEEKRKCFQLAQKLRSFAMRYKVEREQLTQRQEELTTELASLKGTDYLSAV  540
```

```
Ltar   540   GHFDSTAGLGSPGSPGSPGSPRFPRDGEEFNDIERAQAQIRAYRAERMELIVYQAKAANA  599
             G F+ T       SP SPGSP+F RDGE+FND E AQAQ+RA  AER EL++YQAKAANA
Lmex   541   GTFEPTV------SPASPGSPKFARDGEDFNDAESAQAQLRALWAERTELMLYQAKAANA  594

Ltar   600   IRMLVMERDAALRKAA  615
             IRMLV ER+AA RKAA
Lmex   595   IRMLVKEREAAQRKAA  610
```

C. fasciculata protein aligned to *L. mexicana* protein:

```
 Score           Expect  Method                           Identities    Positives     Gaps

 949 bits(2452)  0.0     Compositional matrix adjust.   455/622(73%)  523/622(84%)  16/622(2%)

Cfas   1     MSRAQTSSKSAPKNISVFCRVRPPVSHEKNHTFDNITYDARDDRAIMVNRKSGTKMIEKR  60
             MSR Q SSKSAPK ISV+CRVRPPV  EK H F+NI YD  D+R I V RKSG+K  EKR
Lmex   1     MSRIQKSSKSAPKTISVYCRVRPPVPQEKGHNFNNIVYDDADNRTITVTRKSGSKSFEKR  60

Cfas   61    YMFNRVFQPNVAQKDVYETFAKGAVEAAFDGQHGVLFVYGQTGSGKTFTISNDDPKNEGV  120
             Y FNRVF+P V QKDVYETFAK AV+AAFDGQHGVLFVYGQTGSGKTFTISNDDPKNEGV
Lmex   61    YFFNRVFRPTVTQKDVYETFAKNAVDAAFDGQHGVLFVYGQTGSGKTFTISNDDPKNEGV  120

Cfas   121   LQQSMKEIWGKIAADKENDYSCSVSYVQLYNEILTDLLDEQKGRVRIQIGAEGRGDVVMV  180
             LQQSM+EIW +IA D  NDYSCSVSYVQLYNEILTDLLD+ KG+VRIQ+G EGRGD+VMV
Lmex   121   LQQSMREIWDRIAKDPGNDYSCSVSYVQLYNEILTDLLDDSKGKVRIQMGLEGRGDIVMV  180

Cfas   181   SDATGLPIERPVKDYKGTMNAFKTGMARKEMASTSMNNTSSRSHTVFTLNINKSRKTGAV  240
             SDATGLP+ER VKDYKGTM  FK G+ RKEMASTSMNNTSSRSHT+FTLN+ K+++ G V
Lmex   181   SDATGLPVEREVKDYKGTMAFFKAGLTRKEMASTSMNNTSSRSHTIFTLNVCKAQRVGTV  240

Cfas   241   EVGG-GGGAAVALEGRLVLCDLAGSERVSKTHAEGKTLDEATHINRSLLTLGKVVAALTE  299
              VG    G  +ALEGRLVLCDLAGSERVSKTHAEGKTLDEATHINRSLLTLGKVV ALT+
Lmex   241   TVGAETEGPTIALEGRLVLCDLAGSERVSKTHAEGKTLDEATHINRSLLTLGKVVTALTD  300

Cfas   300   NAQHAPFRESKLTRILQYSLMGNGNTSLVVNISPSDDNIEESLSAIMFGQRASQIKQDAK  359
             NAQHAPFRESKLTRILQYSL+GNGNTS++VNISPSD+N EESLS + FGQRASQIKQDAK
Lmex   301   NAQHAPFRESKLTRILQYSLLGNGNTSIIVNISPSDENTEESLSTLFFGQRASQIKQDAK  360

Cfas   360   RHEVLDYKALYLQLMADLDNKNDKTLEDALDEERGVYEDRISALNDQIKLLTDENTMLRK  419
             RHEVLDYKALYLQLMAD+DNKNDKTLE+AL+EERGVYEDRIS+LN+++KLL +EN MLR
Lmex   361   RHEVLDYKALYLQLMADIDNKNDKTLEEALEEERGVYEDRISSLNEEMKLLNNENAMLRN  420

Cfas   420   ENGQLRAVVPKDKLKLIVQTPGGGAAGAAADGAAGGEEQMSGGGGGGDWAEANRQLREMI  479
             EN QLR  VP D+LKLI +TP  G  G            ++GG   G WA+AN++LRE+I
```

```
Lmex   421  ENKQLRQYVPADRLKLIDETPSSGVPG------------VNGGSISGGWAKANQELRELI  468


Cfas   480  GLRDAKLRTISDERVRLALLLSEEQRKCFKLAQKMQAFGLKYKMERSQLTHRQDELAAEL  539
              RD K++ IS+ERVRLAL+++EE+RKCF+LAQK+++F ++YK+ER QLT RQ+EL  EL
Lmex   469  QQRDEKMKVISNERVRLALVVAEEKRKCFQLAQKLRSFAMRYKVEREQLTQRQEELTTEL  528


Cfas   540  AASKGTDYLSAMGAFDMYTSPSSPRPP---REGEEFNDLEKAQAQIRAFRAERQELIVYQ  596
            A+ KGTDYLSA+G F+   SP+SP  P   R+GE+FND E AQAQ+RA  AER EL++YQ
Lmex   529  ASLKGTDYLSAVGTFEPTVSPASPGSPKFARDGEDFNDAESAQAQLRALWAERTELMLYQ  588


Cfas   597  VKAASAIRMLVKERDAALRKAG  618
             KAA+AIRMLVKER+AA RKA
Lmex   589  AKAANAIRMLVKEREAAQRKAA  610
```

T. brucei protein aligned to *L. mexicana* protein:

Score	Expect	Method	Identities	Positives	Gaps
651 bits(1680)	0.0	Compositional matrix adjust.	336/617(54%)	430/617(69%)	27/617(4%)

```
Tbru   6    SGKAAPKNISVFLRVRPPVPRELKGGTFNNLVCDPSDPQRVTITRGGSARKGTKSFLFNR  65
            S K+APK ISV+ RVRPPVP+E KG  FNN+V D +D + +T+TR   ++   K + FNR
Lmex   7    SSKSAPKTISVYCRVRPPVPQE-KGHNFNNIVYDDADNRTITVTRKSGSKSFEKRYFFNR  65


Tbru   66   VFDPECTQQTIYNEVARGAVDAAFDGQHGVLFVYGQTGSGKTFTISNNDPEKPGVLQQSL  125
            VF P  TQ+ +Y   A+ AVDAAFDGQHGVLFVYGQTGSGKTFTISN+DP+  GVLQQS+
Lmex   66   VFRPTVTQKDVYETFAKNAVDAAFDGQHGVLFVYGQTGSGKTFTISNDDPKNEGVLQQSM  125


Tbru   126  RDIWDRFQADTEYDYSCTVSYVQLYNEMLTDLLDPQGGRVRIQLGPEGRGDVVLVTEASG  185
            R+IWDR   D   DYSC+VSYVQLYNE+LTDLLD   G+VRIQ+G EGRGD+V+V++A+G
Lmex   126  REIWDRIAKDPGNDYSCSVSYVQLYNEILTDLLDDSKGKVRIQMGLEGRGDIVMVSDATG  185


Tbru   186  ASIERKVESYEDCLKYFYEGMDRKEMTSTKMNNTSSRSHTVFNFNLTRSAKVKTVDLSSA  245
              +ER+V+ Y+  + +F  G+ RKEM ST MNNTSSRSHT+F  N+ ++ +V TV + +
Lmex   186  LPVEREVKDYKGTMAFFKAGLTRKEMASTSMNNTSSRSHTIFTLNVCKAQRVGTVTVGAE  245


Tbru   246  KANNEPVIALQGRLVVCDLAGSERASRTNAEGKTLDEATHINGSLLVLGKVVAALTESGS  305
                 P IAL+GRLV+CDLAGSER S+T+AEGKTLDEATHIN SLL LGKVV ALT++ +
Lmex   246  TEG--PTIALEGRLVLCDLAGSERVSKTHAEGKTLDEATHINRSLLTLGKVVTALTDN-A  302


Tbru   306  QHAPFRESKLTRILQYSLLGNGNTSIVVNCSPCDDSTEETLGAIMFGQRAIQIKQDAKRH  365
            QHAPFRESKLTRILQYSLLGNGNTSI+VN SP D++TEE+L  + FGQRA QIKQDAKRH
Lmex   303  QHAPFRESKLTRILQYSLLGNGNTSIIVNISPSDENTEESLSTLFFGQRASQIKQDAKRH  362
```

```
Tbru   366  EILDYKALYYQLLADLDSKNDRTLETALSEERTAYEDRIRVLEERIKILTSENDMLRRES  425
            E+LDYKALY QL+AD+D+KND+TLE AL EER  YEDRI  L E +K+L +EN MLR E+
Lmex   363  EVLDYKALYLQLMADIDNKNDKTLEEALEEERGVYEDRISSLNEEMKLLNNENAMLRNEN  422

Tbru   426  SQLGGTGPVSGTSTASGAAAAAV--AMGGDDANDWRSLTMKMRRAIEKLDADLKRTDKER  483
             QL    P           ++ V    GG  +  W     ++R  I++ D  +K    ER
Lmex   423  KQLRQYVPADRLKLIDETPSSGVPGVNGGSISGGWAKANQELRELIQQRDEKMKVISNER  482

Tbru   484  VELAQFLALEKNKVNVLAQKLRAESLKHIMENKELTQRVTELSIDNAKLKGTDYIS----  539
            V LA  +A EK K   LAQKLR+ ++++ +E ++LTQR  EL+ + A LKGTDY+S
Lmex   483  VRLALVVAEEKRKCFQLAQKLRSFAMRYKVEREQLTQRQEELTTELASLKGTDYLSAVGT  542

Tbru   540  FQPSAACEDALPLSLDSPRRGTPSSGLSQSINVGDAYLQEQLDKANRQLRVLNEERVELI  599
            F+P+ +     P S  SP+        + +            + A  QLR L  ER EL+
Lmex   543  FEPTVS-----PASPGSPKFARDGEDFNDA------------ESAQAQLRALWAERTELM  585

Tbru   600  VYQMMASKAIRLLHAEK  616
            +YQ  A+ AIR+L  E+
Lmex   586  LYQAKAANAIRMLVKER  602
```

T. cruzi protein aligned to *L. mexicana* protein:

Score	Expect	Method	Identities	Positives	Gaps
697 bits(1799)	0.0	Compositional matrix adjust.	346/623(56%)	453/623(72%)	34/623(5%)

```
Tcru   4    QAGTRTAPKNISVFLRVRPTIARESKASCNNLKFDPSDPRRVTVTRKSGSRPISKSFVFN  63
            Q  +++APK ISV+ RVRP + +E   + NN+ +D +D R +TVTRKSGS+   K + FN
Lmex   5    QKSSKSAPKTISVYCRVRPPVPQEKGHNFNNIVYDDADNRTITVTRKSGSKSFEKRYFFN  64

Tcru   64   KVFTPTTGQTEVYEDFARGAVDAAFDGQHGVLFVYGQTGSGKTYTMSNDDPKNLGMMQQG  123
            +VF PT  Q +VYE FA+ AVDAAFDGQHGVLFVYGQTGSGKT+T+SNDDPKN G++QQ
Lmex   65   RVFRPTVTQKDVYETFAKNAVDAAFDGQHGVLFVYGQTGSGKTFTISNDDPKNEGVLQQS  124

Tcru   124  LHDVWNRIRNDTEHDYSCSVSYVQLYNEILTDLLDAQKGRVRIQLGPEGCGDVVLVSDSS  183
            + ++W+RI  D  +DYSCSVSYVQLYNEILTDLLD  KG+VRIQ+G EG GD+V+VSD++
Lmex   125  MREIWDRIAKDPGNDYSCSVSYVQLYNEILTDLLDDSKGKVRIQMGLEGRGDIVMVSDAT  184

Tcru   184  GMPIEKKVQNYEETMELFCVGMGRKEMTSTMMNEVSSRSHTIFNFNITRSEKTRTVVVKE  243
            G+P+E++V++Y+ TM  F  G+ RKEM ST MN  SSRSHTIF  N+ ++++  TV V
Lmex   185  GLPVEREVKDYKGTMAFFKAGLTRKEMASTSMNNTSSRSHTIFTLNVCKAQRVGTVTV--  242

Tcru   244  GELNDAPTMALEGRLVICDLAGSERVSKSHAEGKTLDEATHINGSLLVLGKVVAALTDKS  303
            G   + PT+ALEGRLV+CDLAGSERVSK+HAEGKTLDEATHIN SLL LGKVV ALTD +
```

```
Lmex   243  GAETEGPTIALEGRLVLCDLAGSERVSKTHAEGKTLDEATHINRSLLTLGKVVTALTD-N  301


Tcru   304  SQHVPFRESKLTRILQYSLLGNGNTSIVVNCSPSDESTEETLSAIMFGQRAIQIKQDARR  363
            +QH PFRESKLTRILQYSLLGNGNTSI+VN SPSDE+TEE+LS + FGQRA QIKQDA+R
Lmex   302  AQHAPFRESKLTRILQYSLLGNGNTSIIVNISPSDENTEESLSTLFFGQRASQIKQDAKR  361


Tcru   364  HEVLDYKALYLQLMAELDSKNDGTLADALKEERRVYEDRVSALEDRVRILTTENDLLRQE  423
            HEVLDYKALYLQLMA++D+KND TL +AL+EER VYEDR+S+L + +++L  EN +LR E
Lmex   362  HEVLDYKALYLQLMADIDNKNDKTLEEALEEERGVYEDRISSLNEEMKLLNNENAMLRNE  421


Tcru   424  ---------------ITELQRGGDGTATDGAAAAGWQAVSKQMRDKLAERDAEIKVVTEE  468
                           I E    G      G+ + GW   ++++R+ + +RD ++KV++ E
Lmex   422  NKQLRQYVPADRLKLIDETPSSGVPGVNGGSISGGWAKANQELRELIQQRDEKMKVISNE  481


Tcru   469  RFKLAILLAEEKRTAFRLAEKLRATMMRYQMDSKQWTRQQERLTIELAQLKGTDYISLTG  528
            R +LA+++AEEKR  F+LA+KLR+  MRY+++ +Q T++QE LT ELA LKGTDY+S  G
Lmex   482  RVRLALVVAEEKRKCFQLAQKLRSFAMRYKVEREQLTQRQEELTTELASLKGTDYLSAVG  541


Tcru   529  DIKECGSSGGSTNQSVFTDSVSVPPQPSPSTGQDDSYMQEQLEKAYNRIRELNQERLERI  588
                             F  +VS     SP   +D    +   E A  ++R L  ER E +
Lmex   542  ---------------TFEPTVSPASPGSPKFARDGEDFNDA-ESAQAQLRALWAERTELM  585


Tcru   589  VYQSKAEKAIRVLYAEKTALEKQ  611
            +YQ+KA  AIR+L  E+ A +++
Lmex   586  LYQAKAANAIRMLVKEREAAQRK  608
```

CLUSTAL O(1.2.4) multiple sequence alignment

```
Lbra       MSRPQASSKSAPKTISVFCRVRPLVPHE-KSHTCNNITYDPNDNRAITVNRKTTTKAGEK      59
Lpan       MSRPQASSKSAPKTISVFCRVRPLVPHE-KSHTCNNITYDPNDNRAITVNRKTTTKAGEK      59
Cfac       MSRAQTSSKSAPKNISVFCRVRPPVSHE-KNHTFDNITYDARDDRAIMVNRKSGTKMIEK      59
Lenr       MSRAQTTSKTAPKTVSVFCRVRPPVPQE-KGHTFDSISYDEKDNRAIVVNRKSGSRTVEK      59
Ltar       MSRTQANNKSAPKTISVYCRVRPPVPSE-KGHTFQNIAYDDGDDRAIVVSRKSGTKSLEK      59
Lmex       MSRIQKSSKSAPKTISVYCRVRPPVPQE-KGHNFNNIVYDDADNRTITVTRKSGSKSFEK      59
Lama       MSRTQTSSKSAPKTISVYCRVRPPVPQE-KGHSFNNIVYDDADNRTITVTRKSGTKSFEK      59
Lmaj       MSRTQTSSKSVPKNIAVYCRVRPPVPNE-KGHTFQNISYDDSDSRAIAVARKSGTKALEK      59
Ltur       MSRTQTSSKSAPKNIAVYCRVRPPVLNE-KGHTFHNISYDGNDSRAITVTRKSGTKALEK      59
Lara       MSRTQTSSKSAPKHIAVYCRVRPPVLNE-KGHTFQNISYDDSDNRAITVTRKSGTKALEK      59
Lger       MSRTQTSSKSAPKNIAVYCRVRPPVLNE-KGHTFQNISYDESDNRAITVTRKSGTKALEK      59
Ldon       MSRTQTSSKSAPKTIAVYCRVRPPVPNE-KGHTFQNISYDDRDARAITVNRKSGAKSFEK      59
Linf       MSRTQTSSKSAPKTIAVYCRVRPPVPNE-KGHTFQNISYDDRDARAITVNRKSGAKSFEK      59
Laet       MSRAQTSSKSAPKTIAVYCRVRPPVPHE-KGHTFQNINYDDGDSRAIIVNRKSGTKSLEK      59
Ltro       MSRTQTSSKSAPKTIAVYCRVRPPVPHE-KGDTFQNISYDDNDSRAITVNRKSGTKSIEK      59
tbru       -MAKPLSGKAAPKNISVFLRVRPPVPRELKGGTFNNLVCDPSDPQRVTITRGGSARKGTK      59
tcru       -MKGQAGTRTAPKNISVFLRVRPTIARESKA-SCNNLKFDPSDPRRVTVTRKSGSRPISK      58
                   ::.** ::*: **** :  * *  . ..:  *  * : : : *   ::   *

Lbra       KFMFNRVFQPNSTQKEVYEAFAKGAVDAAFDGQHGVLFVYGQTGSGKTFTISNDDPKSEG      119
Lpan       KFMFNRVFQPNSTQKEVYEAFAKNAVDAAFDGQHGVLFVYGQTGSGKTFTISNDDPKNEG      119
Cfac       RYMFNRVFQPNVAQKDVYETFAKGAVEAAFDGQHGVLFVYGQTGSGKTFTISNDDPKNEG      119
Lenr       KYFFNRVFRPTVSQKEVYECFAKNAVEAAFDGQHGVLFVYGQTGSGKTFTISNDDPQNEG      119
Ltar       RFLFNRVFQPNATQREVYDTFAKSAVEAAFDGQQGVLFVYGQTGSGKTFTISSDDPNNEG      119
Lmex       RYFFNRVFRPTVTQKDVYETFAKNAVDAAFDGQHGVLFVYGQTGSGKTFTISNDDPKNEG      119
```

```
Lama      RYFFNRVFRPTVTQKDVYETFAKNAVDAAFDGQHGVLFVYGQTGSGKTFTISNDDPKNEG      119
Lmaj      TYLFNRVFRPTATQKDVYETFAKGAVDAAFDGQHGVLFVYGQTGSGKTFTISNDEPNNEG      119
Ltur      RYLFNRVFRPNATQKDVYETFAKNAVEAAFDGQHGVLFVYGQTGSGKTFTISNDDPNNEG      119
Lara      RYLFNRVFRPNATQKDVYETFAKNAVDAAFDGQHGVLFVYGQTGSGKTFTISNDDPNNEG      119
Lger      RYLFNRVFRPNATQKDVYETFAKNAVDAAFDGQHGVLFVYGQTGSGKTFTISNDDPNNEG      119
Ldon      KYLFNRVFRPNVTQKDVYENFARNAVDAAFDGQHGVLFVYGQTGSGKTFTISNDDPNNEG      119
Linf      KYLFNRVFRPNVTQKDVYENFARNAVDAAFDGQHGVLFVYGQTGSGKTFTISNDDPNNEG      119
Laet      KYFFNRVFRPNVTQKDVYETFARSAVDAAFDGQHGVLFVYGQTGSGKTFTISNDDPNNEG      119
Ltro      RYLFNRVFRPNVTQKDVYETFARNAVEAAFDGQHGVLFVYGQTGSGKTFTISNDDPNNEG      119
tbru      SFLFNRVFDPECTQQTIYNEVARGAVDAAFDGQHGVLFVYGQTGSGKTFTISNNDPEKPG      119
tcru      SFVFNKVFTPTTGQTEVYEDFARGAVDAAFDGQHGVLFVYGQTGSGKTYTMSNDDPKNLG      118
           :.**:** *   *  :*: .*:.**:******:**************:*:*.::*:. *

Lbra      VLQQSMRDIWAKIANDTEHDYSCSVSYVQLYNEILTDLLDEKKGKVRIQLGTEGCGDVVL      179
Lpan      VLQQSMRDIWTKIANDTEHDYSCSVSYVQLYNEILTDLLDEKKGKVRIQLGTEGCGDVVL      179
Cfac      VLQQSMKEIWGKIAADKENDYSCSVSYVQLYNEILTDLLDEQKGRVRIQIGAEGRGDVVM      179
Lenr      VLQQAMKAIWTKIAEDKANDYTCSVSYVQLYNEILTDLLDDQKGKVRIQMGTEGRGDVLM      179
Ltar      VLQQSVKEIWKKIANDPTNDYSCSVSYVQLYNEILTDLLDEQKGKVRIQIGSEGRGDVVM      179
Lmex      VLQQSMREIWDRIAKDPGNDYSCSVSYVQLYNEILTDLLDDSKGKVRIQMGLEGRGDIVM      179
Lama      VLQQSMREIWGRIAKDPGNDYSCSVSYVQLYNEILTDLLDDSKGKVRIQMGLEGRGDIVM      179
Lmaj      VLQRSMRDIWNRIASDTANDYSCSVSYVQLYNEILTDLLDDAKGKVRIQMGSEGRGDVVM      179
Ltur      VLQQSMREIWSKIASDTANDYSCSVSYVQLYNEILTDLLDDAKSKVRIQIGPEGRGDVVM      179
Lara      VLQQSMREIWNKIASDTANDYSCSVSYVQLYNEILTDLLDDAKSKVRIQMGSEGRGDVVM      179
Lger      VLQQSMREIWSKIASDTANDYSCSVSYVQLYNEILTDLLDDAKSKVRIQMGSEGRGDVVM      179
Ldon      VLQQSMREIWNKIASDTANDYSCSVSYVQLYNEILTDLLDDKKSKVRIQMGSEGRGDVVM      179
Linf      VLQQSMREIWNKIASDTANDYSCSVSYVQLYNEILTDLLDDKKSKVRIQMGSEGRGDVVM      179
Laet      VLQQSMREIWDKVASDTANDYSCSVSYVQLYNEILTDLLDDKKGKVRIQMGSEGRGDVVM      179
Ltro      VLQQSMREIWNKVTSDTANDYSCSVSYVQLYNEILTDLLDDKKGKVRIQMGSEGRGDIVM      179
tbru      VLQQSLRDIWDRFQADTEYDYSCTVSYVQLYNEMLTDLLDPQGGRVRIQLGPEGRGDVVL      179
tcru      MMQQGLHDVWNRIRNDTEHDYSCSVSYVQLYNEILTDLLDAQKGRVRIQLGPEGCGDVVL      178
          ::*:.:: :* :.  *   **:*:*********:******   .:****:* ** **:::

Lbra      VSDDSGKGIEREVKDYNGTMNFFKTGLSRKEMASTAMNNTSSRSHTVFTLNISRSTKVTA      239
Lpan      VSDGSGKGIEREVKDYNGTMNFFKTGLSRKEMASTAMNNTSSRSHTVFTLNISRCTKVTA      239
Cfac      VSDATGLPIERPVKDYKGTMNAFKTGMARKEMASTSMNNTSSRSHTVFTLNINKSRKTGA      239
Lenr      VSDATGLGIEREVTDYKTTMSYFKTGLSRKEMASTSMNNTSSRSHTVFTLNINKSKKVGA      239
Ltar      VSDSTGLAIEREVKDMQSTMSFFKAGLTRKEMGSTSMNDRSSRSHTVFTLHIVKSKKVGT      239
Lmex      VSDATGLPVEREVKDYKGTMAFFKAGLTRKEMASTSMNNTSSRSHTIFTLNVCKAQRVGT      239
Lama      VSDATGLPVEREVKDYKGTMAFFKAGLARKEMASTSMNNTSSRSHTIFTLNVCKAQRVGT      239
Lmaj      VSDSTGMAIEREVKDYKSTMACFQVGLARKEMASTSMNSTSSRSHTIFTLNIVKAKKVVA      239
Ltur      VSDSTGMAIEREVKDFKSTMACFKAGLARKEMASTSMNNTSSRSHTIFTLNIVKAKKVVA      239
Lara      VSDSTGMAIEREVKDFKSTMACFKVGLARKEMASTSMNNTSSRSHTIFTLNIVKAKKVVA      239
Lger      VSDSTGVAIEREVKDYKSTMACFKAGLARKEMASTSMNNTSSRSHTIFTLNIVKAKKVVA      239
Ldon      VSDSTGLAVEREVKDYKSTMAYFKAGLARKEMASTSMNNTSSRSHTIFTLNIVKAKKVGA      239
Linf      VSDTTGLAVEREVKDYKSTMAYFKAGLARKEMASTSMNNTSSRSHTIFTLNIVKAKKVGA      239
Laet      VSDSTGMAIEREVKDYKSTMAYFKGGLARKEMASTSMNNTSSRSHTVFTLNIVKAKKVVA      239
Ltro      VSDSTGTAIEREVKDYKSTMAYFKAGLARKEMASTSMNNTSSRSHTVFTLSIVKAKKVVA      239
tbru      VTEASGASIERKVESYEDCLKYFYEGMDRKEMTSTKMNNTSSRSHTVFNFNLTRSAKVKT      239
tcru      VSDSSGMPIEKKVQNYEETMELFCVGMGRKEMTSTMMNEVSSRSHTIFNFNITRSEKTRT      238
          *:: :*  :*: * . :  :  *  *: **** ** **. ******:*.: : :. :. :

Lbra      VTVG--ADSGGATVALDGRLVLCDLAGSERVSKTHAEGKTLDEATHINRSLLTLGKVVAA      297
Lpan      VTVG--GDSGGATVALDGRLVLCDLAGSERVSKTHAEGKTLDEATHINRSLLTLGKVVAA      297
Cfac      VEVG--GG-GGAAVALEGRLVLCDLAGSERVSKTHAEGKTLDEATHINRSLLTLGKVVAA      296
Lenr      VTVG--AESEGPAVALEGRLVLCDLAGSERVSKTHAEGKTLGEATHINRSLLTLGKVVVA      297
Ltar      VTVG--SETEGQVVALEGRLVLCDLAGSERVSKTHAEGKTLDEAMHINRSLLTLGKVVNA      297
Lmex      VTVG--AETEGPTIALEGRLVLCDLAGSERVSKTHAEGKTLDEATHINRSLLTLGKVVTA      297
Lama      VTVG--AETEGPTIALEGRLVLCDLAGSERVSKTHAEGKTLDEATHINRSLLTLGKVVTA      297
Lmaj      VTVG--TEAEGPTIALEGRLVLCDLAGSERVSKTHAEGKTLDEATHINRSLLTLGKVVTA      297
Ltur      VTVG--TETEGPTVALEGRLVLCDLAGSERVCKTRAEGKTLDEATHINRSLLTLGKVVTA      297
Lara      VTVG--TETEGPTVALEGRLVLCDLAGSERVSKTRAEGKTLDEATHINRSLLTLGKVVTA      297
Lger      VTVG--TETEGPTVALEGRLVLCDLAGSERVSKTRAEGKTLDEATHINRSLLTLGKVVTA      297
Ldon      VTVG--AETEGPTVALEGRLVLCDLAGSERVSKTHAEGKTLDEATHINRSLLTLGKVVTA      297
Linf      VTVG--AETEGPTVALEGRLVLCDLAGSERVSKTHAEGKTLDEATHINRSLLTLGKVVTA      297
Laet      VTVG--AETDGPTVALEGRLVLCDLAGSERVSKTHAEGKTLDEATHINRSLLTLGKVVTA      297
Ltro      VTVG--AEADGPTVALEGRLVLCDLAGSERVSKTHAEGKTLDEATHINRSLLTLGKVVTA      297
tbru      VDLSSAKANNEPVIALQGRLVVCDLAGSERASRTNAEGKTLDEATHINGSLLVLGKVVAA      299
tcru      VVVKEGELNDAPTMALEGRLVICDLAGSERVSKSHAEGKTLDEATHINGSLLVLGKVVAA      298
```

```
          * :         .:**:****:********..::.******.** *** ***.***** *

Lbra      LTAN-AQHAPFRESKLTRILQYSLMGNGNTSIVVNVSPSDENTEETFSAILFGQRASQIK      356
Lpan      LTAN-AQHAPFRESKLTRILQYSLMGNGNTSIVVNVSPSDENTEETFSAILFGQRASQIK      356
Cfac      LTEN-AQHAPFRESKLTRILQYSLMGNGNTSLVVNISPSDDNIEESLSAIMFGQRASQIK      355
Lenr      LTEN-AQHAPFRESKLTRILQYSLMGNGNTSLVVNISPSDSNTEESLSAIMFGQRASQIK      356
Ltar      LTDN-AQHAPFRESKLTRILQYSLMGNGNTSIIVNIGPSDSNTEESLSAIVFGQRASQIK      356
Lmex      LTDN-AQHAPFRESKLTRILQYSLLGNGNTSIIVNISPSDENTEESLSTLFFGQRASQIK      356
Lama      LTDN-AQHAPFRESKLTRILQYSLLGNGNTSIIVNISPSDENTEESLSTLFFGQRASQIK      356
Lmaj      LTDN-AQHAPFRESKLTRILQYSLMGNGNTSIIVNISPSDDNTEESLSAILFGQRASQIK      356
Ltur      LTDN-AQHAPFRESKLTRILQYSLMGNGNTSIIVNISPSDDNTEESLSAILFGQRASQIK      356
Lara      LTDN-AQHAPFRESKLTRILQYSLMGNGNTSIIVNISPSDDNTEESLSAILFGQRASQIK      356
Lger      LTDN-AQHAPFRESKLTRILQYSLMGNGNTSIIVNISPSDDNTEESLSAILFGQRASQIK      356
Ldon      LTDN-AQHAPFRESKLTRILQYSLMGNGNTSIIVNISPSDENTEESLSAILFGQRASQIK      356
Linf      LTDN-AQHAPFRESKLTRILQYSLMGNGNTSIIVNISPSDENTEESLSAILFGQRASQIK      356
Laet      LTDN-AQHAPFRESKLTRILQYSLLGNGNTSIIVNISPSDNNTEESLSAIFFGQRASQIK      356
Ltro      LTDK-AQHAPFRESKLTRILQYSLLGNGNTSIIVNISPSDNNTEESLSAIFFGQRASQIK      356
tbru      LTESGSQHAPFRESKLTRILQYSLLGNGNTSIVVNCSPCDDSTEETLGAIMFGQRAIQIK      359
tcru      LTDKSSQHVPFRESKLTRILQYSLLGNGNTSIVVNCSPSDESTEETLSAIMFGQRAIQIK      358
          ** . :**.***************:******::** .*.*.. **::.::.***** ***

Lbra      QDAKRHEVLDYKALYLQLMADLDNKNDKTLEDALEEERGVFDDRINSLNDQVKMLTEENG      416
Lpan      QDAKRHEVLDYKALYLQLMADLDNKNDKTLEDALEEERGVFDDRINSLNDQVKILTEENG      416
Cfac      QDAKRHEVLDYKALYLQLMADLDNKNDKTLEDALDEERGVYEDRISALNDQIKLLTDENT      415
Lenr      QDAKRHEVLDYKALYLQLMADMDNKNDKTLEDALEEERSVYEDRISNLNDQIKLLSNENS      416
Ltar      QDAKRHEVLNYKALYLQLMADLDNKNDRTLEAALEEERAVYEDRVAALVEEMRLLNDENS      416
Lmex      QDAKRHEVLDYKALYLQLMADIDNKNDKTLEEALEEERGVYEDRISSLNEEMKLLNNENA      416
Lama      QDAKRHEVLDYKALYLQLMADIDNKNDKTLEEALEEERGVYEDRISSLNEEMKLLNNENA      416
Lmaj      QDAKRHEVLDYKALYMQLMAELDNKNDKTLEEALEEERGVYEERISALDEEMKLLSDENA      416
Ltur      QDAKRHEVLDYKALYMQLMADLDSKNDKTLEEALEEERGVYEERISALDEEMKLLSDENA      416
Lara      QDAKRHEVLDYKALYMQLMADLDNKNDKTLEEALEEERGVYEERISALDEEMKLLSDENA      416
Lger      QDAKRHEVLDYKALYMQLMADLDNKNDRTLEEALEEERGVYEERISALDEEMKLLSDENA      416
Ldon      QDAKRHEVLDYKALYLQLMADLDNKNDKTLEEALEEERGVYEERISALDEEMKLLSDENA      416
Linf      QDAKRHEVLDYKALYLQLMADLDNKNDKTLEEALEEERGVYEERISALDEEMKLLSDENA      416
Laet      QDAKRHEVLDYKALYLQLMADMDNKNDKTLEEALEEERGVYEERISALDEEMKLLNNENA      416
Ltro      QDAKRHEVLDYKALYLQLMADMDNKNDKTLEEALEEERGVYEERISALDEEMKLLNNENV      416
tbru      QDAKRHEILDYKALYYQLLADLDSKNDRTLETALSEERTAYEDRIRVLEERIKILTSEND      419
tcru      QDARRHEVLDYKALYLQLMAELDSKNDGTLADALKEERRVYEDRVSALEDRVRILTTEND      418
          ***:***:*:***** **:*::*.*** **  **.*** .:::*:  * :.:::*. **

Lbra      MLRNENKQLRQCVPADKLKTIDETPASGVP------------AYEGASGGGNWAKANQEL      464
Lpan      MLRNENKQLRQCVPPDKLKTIDETPASGVP------------AYEGASGGGSWAKANQEL      464
Cfac      MLRKENGQLRAVVPKDKLKLIVQTPGGGAAGAAADGAAGGEEQMSGGGGGGDWAEANRQL      475
Lenr      MLRNENSMLRSAVPPEKLKMIDETPASGVAVP---------DGAAGGGGGGNWAKANQEL      467
Ltar      MLRNENNRLRHYVPPDKLKMIDQIPASGVS------------GAS-EAGSGGWAKANQEL      463
Lmex      MLRNENKQLRQYVPADRLKLIDETPSSGVP------------GVNGGSISGGWAKANQEL      464
Lama      MLRNENKQLRQYVPADRLKLIDETPSSGVP------------GVNGGSISGGWAKANQEL      464
Lmaj      MLRNENKQLRQYVPADRLKLIDETPSSGVS------------GANGEVVSGGWAKVNQDL      464
Ltur      MLRNENKQLRQYVPADRLKLIDETPCSGVS------------GANGGVVSGGWAKVNQDL      464
Lara      MLRNENRQLRQYVPADRLKLIDETPCSGVS------------GANGGVVSGGWAKVNQDL      464
Lger      MLRNENRQLRQYVPADRLKLIDETPFSGVS------------GANGGVVSGGWAKVNQDL      464
Ldon      MLRNENKQLRQYVPADRLKLIDETPSSGVP------------GANGEVASGGWAKANQDL      464
Linf      MLRNENKQLRQYVPADRLKLIDETPSSGVP------------GANGEVASGGWAKANQDL      464
Laet      MLRNENKQLRQYVPTDRLKLIDETPSSGVS------------GANGEVVSGGWAKANQDL      464
Ltro      MLRNENKQLRQYVPADRLKLIDETPSSGVH------------GANGEVVSGGWAKANQDL      464
tbru      MLRRESSQLGGTGPVSGTSTA-------------SG-AAAAAVAMGGDDANDWRSLTMKM      465
tcru      LLRQEITELQRGGDG---------------------------TATDGAAAAGWQAVSKQM      451
          :**.*   *                                        . .*   . .:

Lbra      REVIKARDEKLRTINEERLRLALVVAEEQRKCFQLAQKMRAFALRYKMEREQSSRRQDAL      524
Lpan      REVIKSRDEKLRTINEERLRLALVVAEEQRKCFQLAQKMRAFALRYKMEREQSSRRQDAL      524
Cfac      REMIGLRDAKLRTISDERVRLALLLSEEQRKCFKLAQKMQAFGLKYKMERSQLTHRQDEL      535
Lenr      REMIKLRDEKLRAISDERVRLALVLAEEQRKCFQLAQKMRSFASRYKLERAQAIRRQEEL      527
Ltar      RELVRQRDEKLKVISDERVRLALVVAEEQRKCFQLAQKMRSFAMRYKMERELSTQRQEEL      523
Lmex      RELIQQRDEKMKVISNERVRLALVVAEEKRKCFQLAQKLRSFAMRYKVEREQLTQRQEEL      524
Lama      RELIQQRDEKMKVISNERVRLALVVAEEKRKCFQLAQKLRSFAMRYKVEREQLTQRQEEL      524
Lmaj      RKLVQQRDERLKVISDERVRLALVVAEEKRKCFQLAQKMRSFAMRYKMEREQSTQRQEEL      524
Ltur      RELVQQRDARLKVISEERVRLALVVAEEKRKCFQLAQKMRSFAIRYKMEREQSTQRQEEL      524
```

```
Lara      RELVQQRDERLKVISDERVRLALVVAEEKRKCFQLAQKMRSFAMRYKMEREQSTQRQEEL      524
Lger      RELVQQRDERLKVISDERVRLALVVAEEKRKCFQLAQKMRSFAMRYKMEREQSTQRQEEL      524
Ldon      RELVKQRDDKLKVISDERVRLALVVAEEKRKCFQLAQKMRSFAMRYKMEREQSTQRQEEL      524
Linf      RELVKQRDDKLKVISDERVRLALVVAEEKRKCFQLAQKMRSFAMRYKMEREQSTQRQEEL      524
Laet      RELVKQRDEKLKVISDERVRLALVVAEEKRKCFQLAQKMRSFAMRYKMEREQSTQRQEEL      524
Ltro      RELVRQRDEKLKVISDERVRLALVVAEEKRKCFQLAQKMRSFAMRYKMEREQSTQRQEEL      524
tbru      RRAIEKLDADLKRTDKERVELAQFLALEKNKVNVLAQKLRAESLKHIMENKELTQRVTEL      525
tcru      RDKLAERDAEIKVVTEERFKLAILLAEEKRTAFRLAEKLRATMMRYQMDSKQWTRQQERL      511
          *  :   *  ::   .**..** .:: *:..   **:*:::   :: ::     ::   *

Lbra      TAELASVKGTDYLSALGTFDSSM-------------SPASPRGG------------SDAE      559
Lpan      TAELASVKGTDYLSALGTFDSSM-------SPASPGSPGSPRGG------------SDGE      565
Cfac      AAELAASKGTDYLSAMGAFDMYT-------------SPSSPRPP------------REGE      570
Lenr      CAELAAVKGTDYLSALGNFDAALT-------------AGSARTS------------REAE      562
Ltar      SAELAALKGTEYLSTLGHFDSTAGLGSP----GSPGSPGSPRFP------------RDGE      567
Lmex      TTELASLKGTDYLSAVGTFEPTV----------SPASPGSPKFA------------RDGE      562
Lama      TTELASLKGTDYLSAVGTFDPTV----------SPATPGSPKFA------------RDGE      562
Lmaj      CTELATLKGTDYLSAVGSFDATA-------------SPGSPNYP------------RENE      559
Ltur      SAELATLKGTDYLSAVGNFDATS-------------SPGSPNFP------------RENE      559
Lara      CAELATLKGTDYLSAVGNFDATL-------------SPGSPNFP------------RENE      559
Lger      CAELATLKGTDYLSAVGNFDATS-------------NPGSPNFP------------RENE      559
Ldon      CAELAALKGTDYLSAVGNFDATA----------SPGSPGSPHYP------------REGE      562
Linf      CAELAALKGTDYLSAVGNFDATA----------SPGSPGSPHYP------------REGE      562
Laet      CAELATLKGTDYLSAVGTFDAVA----------SPGSPGSPNCS------------REGE      562
Ltro      CAELATLKGTDYLSAVGTFDAAA----------SPGSPGSPNYS------------REGE      562
tbru      SIDNAKLKGTDYISFQPSAA-CED--------ALPLSLDSPRRGTPSSGLSQSINVGDAY      576
tcru      TIELAQLKGTDYISLTGDIKECGSSGGSTNQSVFTDSVSVPPQPSP------STGQDDSY      565
            : *  ***:*:*                                           :

Lbra      DYNELESARQQIRSLRAERADLIMYQKKAAEAIRVLASERDAALRKAA------------      607
Lpan      DYNELESARQQIRALRAERTDLIMYQKKAAEAIRVLASERDAALRKAA------------      613
Cfac      EFNDLEKAQAQIRAFRAERQELIVYQVKAASAIRMLVKERDAALRKAGQN----------      620
Lenr      DFSDNDRAQALIRALRAERMELIVYQAKAASAIRMLVKERDAALREKSA-----------      611
Ltar      EFNDIERAQAQIRAYRAERMELIVYQAKAANAIRMLVMERDAALRKAA------------      615
Lmex      DFNDAESAQAQLRALWAERTELMLYQAKAANAIRMLVKEREAAQRKAA------------      610
Lama      DFNDAESAQAQLRALWAERTELMLYQAKAANAIRMLVKEREAAQRKAA------------      610
Lmaj      EFNDAESAQAQIRALRAERMELMVYQAKAANAIRKLVKERDAAQRKVA------------      607
Ltur      EFNEVESAQAEIRALRAERRELMVYQAKAANAIRKLVSERDAAQRK--------------      605
Lara      EFNEAESAQAEIRALRAERRELMVYQAKAANAIRKLVKERDAAQRK--------------      605
Lger      EFNEAESAQAEIRALRAERRELMVYQAKAANAIRKLVKERDAAQRK--------------      605
Ldon      EFNDAESAQAQIRALRAERTELMVYQAKAANAIRMLVKERDAAQRKVA------------      610
Linf      EFNDAESAQAQIRALRAERTELMVYQAKAANAIRMLVKERDAAQRKVA------------      610
Laet      DFNDAESAQAQIRALRAERAELMLYQAKAANAIRKLVKERDAAQRKVA------------      610
Ltro      DFNDAESAQAQIRALRAERAELMVYQAKAANAIRRLVEERDAAQRKVA------------      610
tbru      LQEQLDKANRQLRVLNEERVELIVYQMMASKAIRLLHAEKTSLANHLEKLKA--------      628
tcru      MQEQLEKAYNRIRELNQERLERIVYQSKAEKAIRVLYAEKTALEKQLLQQHLVSKEKTTT      625
            .: : *   :*    ** : ::**  * .*** *  *: :  ..

Lbra      -------      607
Lpan      -------      613
Cfac      -------      620
Lenr      -------      611
Ltar      -------      615
Lmex      -------      610
Lama      -------      610
Lmaj      -------      607
Ltur      -------      605
Lara      -------      605
Lger      -------      605
Ldon      -------      610
Linf      -------      610
Laet      -------      610
Ltro      -------      610
tbru      -------      628
tcru      MGSRSSH      632
```

Original images of gels, autoradiographs and blots

Figure 2

M 1 2 3 4

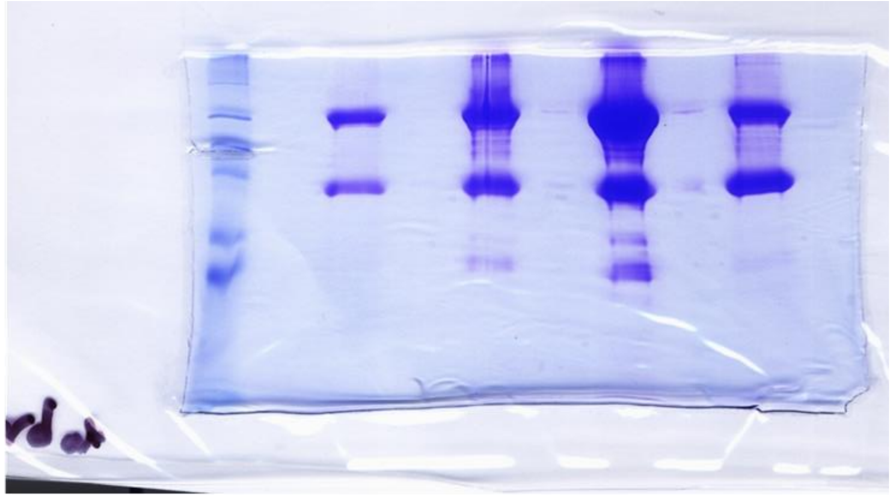

1 2 3 4

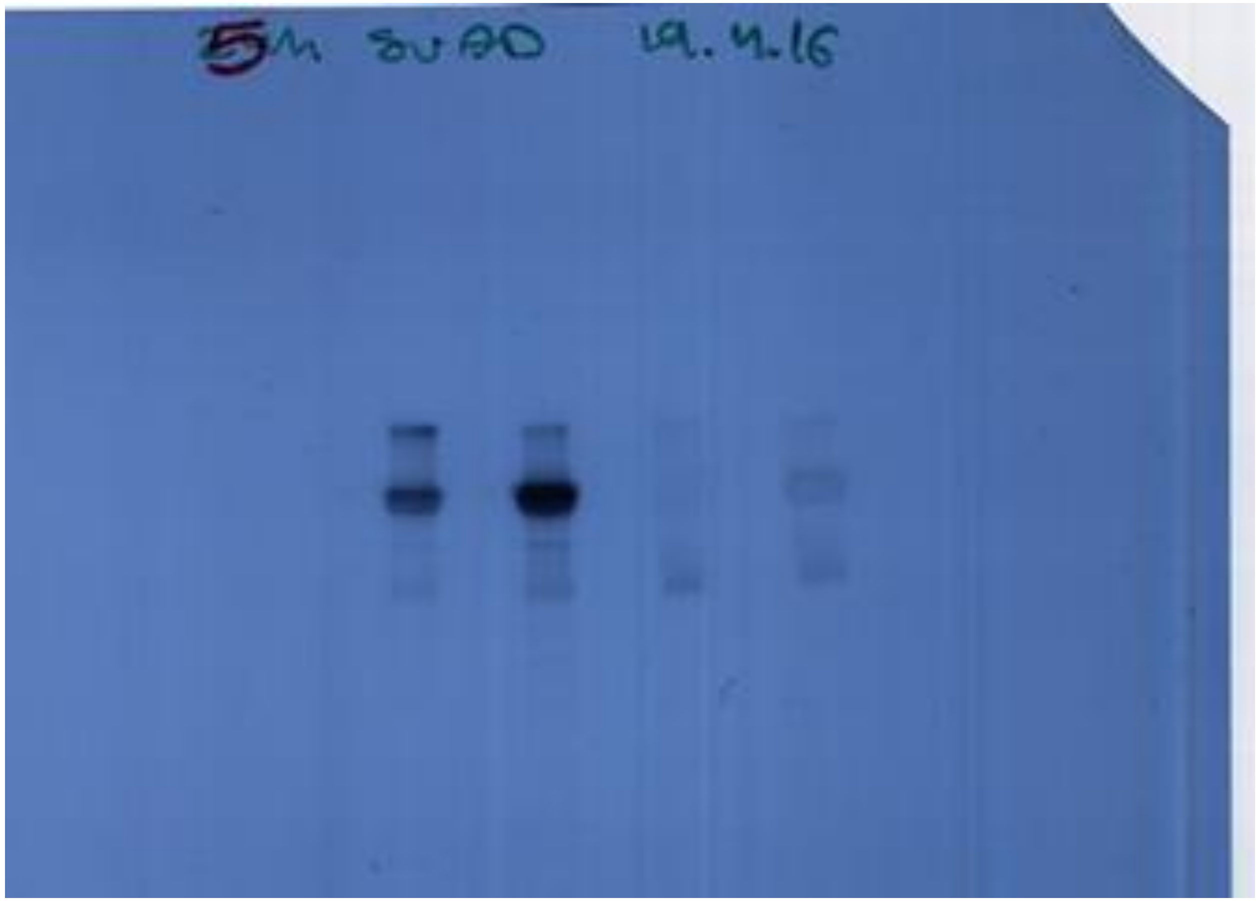

Figure 3

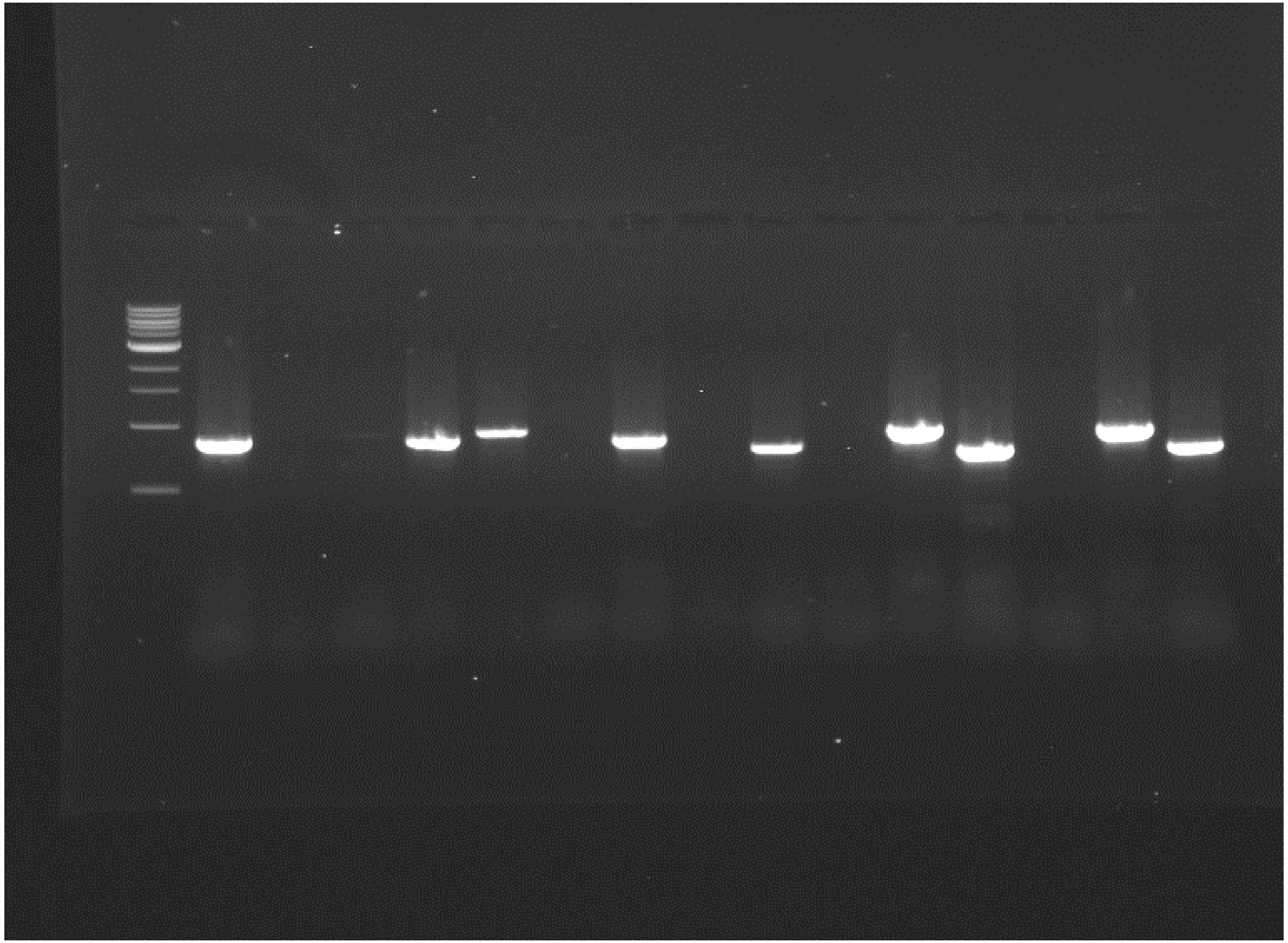

Figure 5E

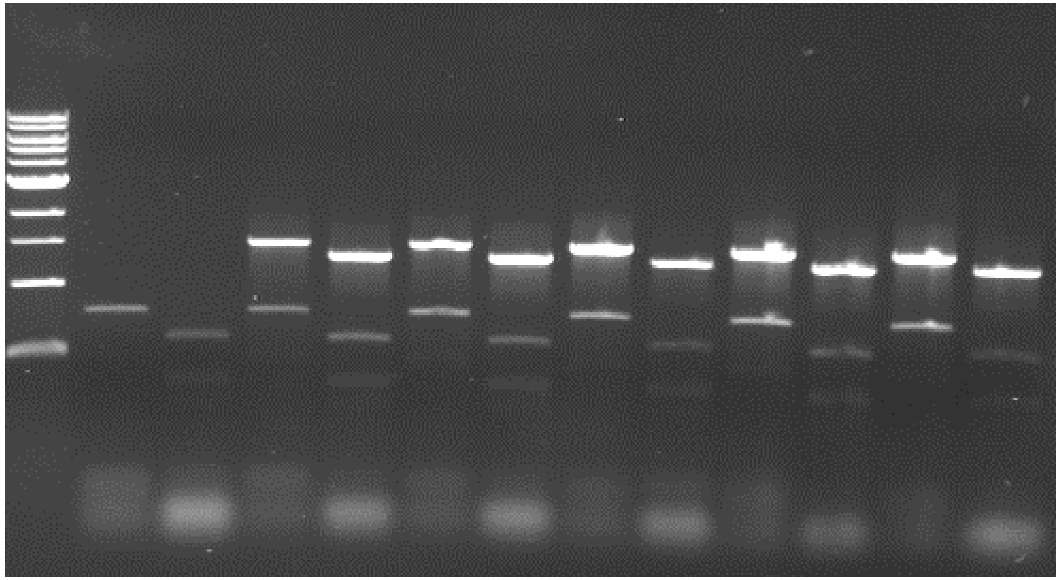

Only lanes 1 – 6 are relevant for the manuscript and show the results of the analysed clones.

Figure 8

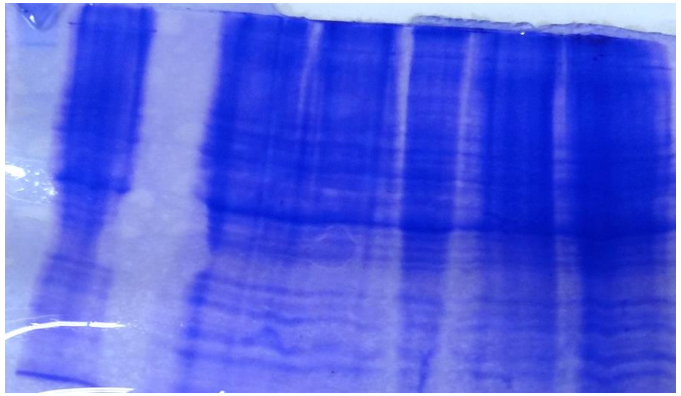

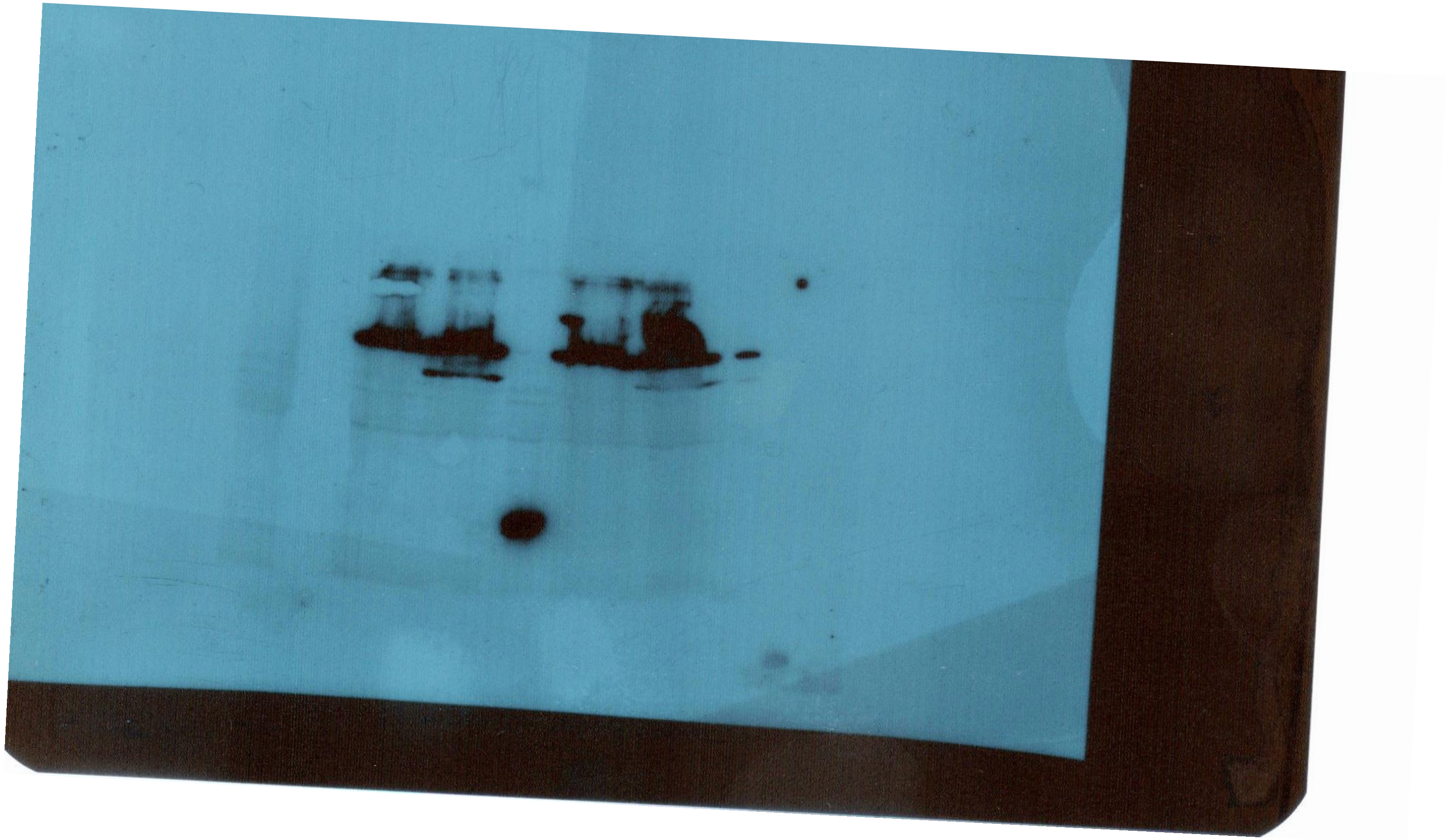

The SDS-PAGE is a loading control for comparable amounts of protein loaded per lane.
The top edge is visible in the right hand corner, the bottom edge is in the left hand corner.
The relevant area for the immunoblot is covered in the gel. The absence of free GFP in lanes 1, 2, 4 and 5 and its presence in lane 3 is clearly shown.
